# Synthesis and *in vitro* antiproliferative and antibacterial activity of new thiazolidine-2,4-dione derivatives

**DOI:** 10.1080/14756366.2017.1387543

**Published:** 2017-11-03

**Authors:** Nazar Trotsko, Agata Przekora, Justyna Zalewska, Grażyna Ginalska, Agata Paneth, Monika Wujec

**Affiliations:** a Department of Organic Chemistry, Faculty of Pharmacy, Medical University of Lublin, Lublin, Poland;; b Department of Biochemistry and Biotechnology, Faculty of Pharmacy, Medical University of Lublin, Lublin, Poland

**Keywords:** Antiproliferative activity, IC_50_, thiazolidinediones, thiosemicarbazones, antibacterial activity

## Abstract

In our present research, we synthesised new thiazolidine-2,4-diones (**12–28**). All the newly synthesised compounds were evaluated for antiproliferative and antibacterial activity. Antiproliferative evaluation was carried out using normal human skin fibroblasts and tumour cell lines: A549, HepG2, and MCF-7. The IC_50_ values were determined for tested compounds revealing antiproliferative activity. Moreover, safety index (SI) was calculated. Among all tested derivatives, the compound **18** revealed the highest antiproliferative activity against human lung, breast, and liver cancer cells. More importantly, the derivative **18** showed meaningfully lower IC_50_ values when compared to the reference substance, irinotecan, and relatively high SI values. Moreover, newly synthesised compounds were screened for the bacteria growth inhibition *in vitro.* According to our screening results, most active compound was the derivative **18** against Gram-positive bacteria. Therefore, it may be implied that the novel compound **18** appears to be a very promising agent for anticancer treatment.

## Graphical abstract

**Figure:**
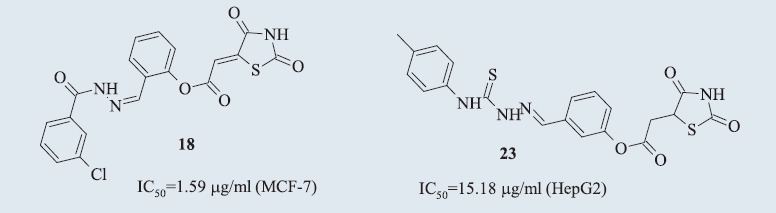

## Introduction

Cancer is one of the most serious health problems in the world. It is the second cause of death after heart diseases worldwide. According to the World Cancer Report 2014, 8.2 million people died from cancer in 2012^1^.

Lung, liver, stomach, and bowel cancer are the most common causes of human deaths worldwide, accounting for nearly a half of all cancer deaths. The five most common types of disease diagnosed in 2012 were lung, prostate, colorectal, stomach, and liver cancer among men; and breast, colorectal, lung, cervix, and stomach cancer among women^1^.

Despite enormous efforts aimed at the implementation of new treatment strategies of chemotherapeutic agents, treatment results in most cases are unsatisfactory^2^. Therefore, there is an urgent need to find new classes of substances with selective action against tumour cells.

Heterocyclic compounds play an important role in cancer therapy. Among them, the derivatives of thiazolidine-2,4-dione are found^3–6^. Researchers’ interest in the derivatives of thiazolidine-2,4-dione has increased recently, the main reason being a wide spectrum of biological properties shown by these derivatives. It has been confirmed by numerous reviews on the activity and mechanisms of action of thiazolidine-2,4-diones^2^ ^,^ ^7^ ^,^ ^8^.

Thiazolidine-2,4-diones are known as antidiabetic drugs and include rosiglitazone, pioglitazone, and darglitazone. Moreover, thiazolidine-2,4-dione derivatives possess biological activities such as aldose reductase inhibitory^9^, antibacterial^6^ ^,^ ^10–13^, antifungal^10^, antitubercular^14^, and anti-inflammatory activity^15^, etc.

Regulation of both cell proliferation and pathways of apoptosis connected with cell death is important in understanding various diseases including malignancies^16^. Therefore, identification of regulators of the cell cycle and apoptosis stimulators is an attractive strategy to explore potential anticancer agents^17^.

There are several mechanisms of anticancer activity of thiazolidine-2,4-dione derivatives already discussed in the literature. The most known ones are induction of apoptosis, cell differentiation, and cell cycle arrest^8^. The thiazolidine-2,4-dione derivatives showing anticancer activity are mainly derivatives modified in the position 5 of the thiazolidine-2,4-dione ring.

Patil et al. reported that 10 new derivatives of 5-benzylidenethiazolidine-2,4-dione had displayed a variable degree of antiproliferative activity against seven tumour cell lines. 2-{4-[(2,4-dioxo-1,3-thiazolidin-5-ylidene)methyl]phenoxy}-N-[3-(trifluoromethyl)phenyl]acetamide showed the most significant effect against breast cancer MCF-7 and leukaemia K562 cell lines^3^.

New 5-substituted pyrazoline thiazolidine-4-one was tested for anticancer activity according to the NCI protocol. The most active compounds were the derivatives of 2-(2,4-dioxo-1,3-thiazolidin-5-ylidene)acetic acid, namely 5-{2-[5-(2-hydroxyphenyl)-3-(4-methoxyphenyl)-4,5-dihydro-1H-pyrazol-1-yl]-2-oxoethylidene}-1,3-thiazolidine-2,4-dione and 5-{2-[5-(2-hydroxyphenyl)-3-(naphthalen-2-yl)-4,5-dihydro-1H-pyrazol-1-yl]-2-oxoethylidene}-1,3-thiazolidine-2,4-dione. These derivatives displayed the highest inhibitory activity against leukaemia cells (HL-60 (TB)) and CNS cancer (SF-295)^5^.

A number of derivatives of 2,4-dioxothiazolidine-5-acetic acid with the ring of 5-substituted-2-amino-1,3,4-thiadiazole showed cytotoxic effects *in vitro* against four human tumour cell lines (cervical carcinoma – HeLa, colorectal cancer – HT29, lung cancer – A549, and breast cancer – MCF-7). Among the 14 derivatives, 2-(2,4-dioxo-1,3-thiazolidin-5-yl)-N-[5-(3,4,5-trimethoxyphenyl)-1,3,4-thiadiazol-2-yl]acetamide showed significant inhibitory activity against the tested cell lines^6^.

Havrylyuk et al. described 2,4-dioxothiazolidine-5-acetic derivatives with benzoxazole or benzothiazole substituents that showed *in vitro* cytotoxic activity against some tumour cell lines: leukaemia (HL-60(TB), K-562, MOLT-4), breast cancer (MDA-MB-231/ATCC), CNS cancer (SF-268), melanoma (LOX IMVI), and prostate cancer, all tested according to the NCI protocol. Among the tested compounds, three were the most active: 3-methoxy-4-(2,4-thiazolidinedione-5-acetoxy)benzylidenehydrazone of benzoxazole-2-thioacetic acid, 3-methoxy-4-(2,4-thiazolidinedione-5-acetoxy)benzylidenehydrazone of benzothiazole-2-thioacetic acid, and 4-(2,4-thiazolidinedione-5-acetoxy)benzylidenehydrazone of benzothiazole-2-thioacetic acid^4^.

The aim of the present research was to synthesise new thiazolidine-2,4-dione derivatives and to evaluate *in vitro* their potential as anticancer and antibacterial agents. As thiosemicarbazone^18–20^ and acylhydrazone derivatives^20–24^ present anticancer activity, which is similar to the activity of the above mentioned thiazolidine-2,4-diones, it was assumed that the structure modification of the thiazolidine-2,4-dione ring in position 5 by thiosemicarbazide and hydrazide derivatives can extend the biological activity of the new compounds.

## Experimental

### Chemistry

#### Materials and methods

Melting points were determined using Fisher-Johns apparatus (Fisher Scientific, Schwerte, Germany) and were not corrected. The ^1^H NMR and ^13 ^C NMR spectra were recorded by a Bruker Avance 300 MHz instrument using DMSO-d_6_ as solvent and TMS as an internal standard. Chemical shifts were expressed as *δ* (ppm). MS using atmospheric pressure chemical ionisation (APCI) was recorded on a Bruker MicroTOF II mass spectrometer. APCI settings were as follows: vaporiser temperature, 350 °C; drying gas temperature, 180 °C; drying gas flow, 4 l/min; and nebuliser pressure, 2 bar. The purity of the compounds was checked by TLC on plates with silica gel Si 60 F_254_, produced by Merck Co. (Darmstadt, Germany). Elemental analyses were performed by AMZ 851 CHX analyser and the results were within ±0.4% of the theoretical value.

#### General procedure for the synthesis of hydrazones (12–21) or thiosemicarbazones (22–28)

Anhydrous ethanol (5–10 ml) was added to the mixture of 0.001 mol hydrazide (3-chlorobenzhydrazide, 2,4-dichlorobenzhydrazide), thiosemicarbazide or appropriate 4-substituted thiosemicarbazide, and 0.001 mol corresponding compounds^5–11^. Then, the mixture was heated under reflux till the dissolving of the substrates. The reflux lasted 5–15 min but in cases of compounds **13**, **14**, **17**, **28** reflux lasted 1.5 h. After that, the mixture was cooled and precipitate was filtered off, dried, and crystallised from butanol or acetic acid.

##### 3-{[2-(3-Chlorobenzoyl)hydrazinylidene]methyl}phenyl (2,4-dioxo-1,3-thiazolidin-5-yl)acetate (12)

Yield 87%, m.p. 244–245 °C, ^1^H NMR *δ* ppm (DMSO-d_6_): 3.45–3.47 m (2H, CH–CH_2_); 4.86–4.90 m (1H, C**H**–CH_2_); 7.20–7.23 m, 7.51–7.70 m, 7.89 d, 7.97 s (8H, Ar, *J* = 7.5 Hz); 8.45 s (1H, CH=N); 12.03 s (1H, NHCO); 12.16 s (1H, NH, thiazolidine), ^13 ^C NMR *δ* ppm (DMSO-d_6_): 36.4 (CH_2_CH), 46.8 (CH_2_ CH), 119.9, 123.9, 126.0, 127.0, 127.8, 130.7, 131.0, 132.2, 133.8, 135.8, 136.4, 147.6 (12 × C_ar._), 151.0 (CH=N), 162.3, 169.6 (2 × C=O), 172.7, 175.9 (2 × C=O, thiazolidine). MS (APCI) [M]^+^ *m/z* = 432.0441. Anal. calc. for C_19_H_14_ClN_3_O_5_S (431.85) (%): C 52.84; H 3.27; N 9.73. Found: C 52.80; H 3.24; N 9.70.

##### 3-{[2-(2,4-Dichlorobenzoyl)hydrazinylidene]methyl}phenyl (2,4-dioxo-1,3-thiazolidin-5-yl)acetate (13)

Yield 95%, m.p. 198–200 °C, ^1^H NMR *δ* ppm (DMSO-d_6_): 3.44–3.47 m (2H, CH–CH_2_); 4.85–4.90 m (1H, C**H**–CH_2_); 7.20–7.24 m, 7.52–7.66 m, 7.79 d (7H, Ar, *J* = 1.8 Hz); 8.27 s (1H, CH=N); 12.06 s (1H, NHCO); 12.15 s (1H, NH, thiazolidine), ^13 ^C NMR *δ* ppm (DMSO-d_6_): 36.4 (CH_2_CH), 46.9 (CH_2_ CH), 120.1, 123.6, 123.9, 126.0, 128.0, 129.1, 129.8, 130.7, 131.2, 132.2, 136.1, 147.6 (12 × C_ar._), 151.0 (CH=N), 162.2, 169.6 (2 × C=O), 172.7, 176.0 (2 × C=O, thiazolidine). MS (APCI) [M]^+^ *m/z* = 466.0046. Anal. calc. for C_19_H_13_Cl_2_N_3_O_5_S (466.29) (%): C 48.94; H 2.81; N 9.01. Found: C 48.90; H 2.77; N 8.99.

##### 4-{[2-(3-Chlorobenzoyl)hydrazinylidene]methyl}phenyl (2,4-dioxo-1,3-thiazolidin-5-yl)acetate (14)

Yield 83%, m.p. 215–216 °C, ^1^H NMR *δ* ppm (DMSO-d_6_): 3.44–3.47 m (2H, CH–CH_2_); 4.85–4.89 m (1H, C**H**–CH_2_); 7.25 d, 7.81 d (4H, 4-O–C_6_H_4_, *J* = 8.7 Hz); 7.55–7.70 m, 7.89 d, 7.97 s (4H, Ar, *J* = 7.5 Hz); 8.46 s (1H, CH=N); 11.98 s (1H, NHCO); 12.16 s (1H, NH, thiazolidine), ^13 ^C NMR *δ* ppm (DMSO-d_6_): 36.4 (CH_2_CH), 46.8 (CH_2_ CH), 122.7, 127.0, 127.8, 128.9, 131.0, 132.1, 132.6, 133.8, 135.8, 147.8 (12 × C_ar._), 151.9 (CH=N), 162.2, 169.4 (2 × C=O), 172.7, 176.0 (2 × C=O, thiazolidine). MS (APCI) [M]^+^ *m/z* = 432.0420. Anal. calc. for C_19_H_14_ClN_3_O_5_S (431.85) (%): C 52.84; H 3.27; N 9.73. Found: C 52.79; H 3.23; N 9.72.

##### 4-{[2-(2,4-Dichlorobenzoyl)hydrazinylidene]methyl}phenyl (2,4-dioxo-1,3-thiazolidin-5-yl)acetate (15)

Yield 61%, m.p. 196–198 °C, ^1^H NMR *δ* ppm (DMSO-d_6_): 3.44–3.47 m (2H, CH–CH_2_); 4.85–4.89 m (1H, C**H**–CH_2_); 7.25 d (2H, 4-O–C_6_H_4_, *J* = 8.7 Hz); 7.52–7.66 m, (2H, Ar); 7.78–7.82 m (3H, Ar); 8.28 s (1H, CH=N); 12.00 s (1H, NHCO); 12.16 s (1H, NH, thiazolidine), ^13 ^C NMR *δ* ppm (DMSO-d_6_): 36.4 (CH_2_CH), 46.8 (CH_2_ CH), 122.7, 127.7, 128.0, 129.0, 131.2, 132.2, 132.4, 134.5, 135.7, 147.7 (12 × C_ar._), 151.9 (CH=N), 162.1, 169.4 (2 × C=O), 172.7, 175.9 (2 × C=O, thiazolidine). MS (APCI) [M]^+^ *m/z* = 465.9945. Anal. calc. for C_19_H_13_Cl_2_N_3_O_5_S (466.29) (%): C 48.94; H 2.81; N 9.01. Found: C 48.92; H 2.75; N 9.00.

##### 4-{[2-(3-Chlorobenzoyl)hydrazinylidene]methyl}-2-methoxyphenyl (2,4-dioxo-1,3-thiazolidin-5-yl)acetate (16)

Yield 92%, m.p. 220–222 °C, ^1^H NMR *δ* ppm (DMSO-d_6_): 3.42–3.44 m (2H, CH–CH_2_); 3.85 s (3H, OCH_3_); 4.84–4.88 m (1H, C**H**–CH_2_); 7.21 d, 7.31–7.34 m, 7.48–7.70 m, 7.87–7.90 m, 7.97 s (7H, Ar, *J* = 8.1 Hz); 8.44 s (1H, CH=N); 12.00 s (1H, NHCO); 12.14 s (1H, NH, thiazolidine), ^13 ^C NMR *δ* ppm (DMSO-d_6_): 36.1 (CH_2_CH), 46.9 (CH_2_ CH), 56.4 (OCH_3_), 110.5, 121.1, 123.6, 127.0, 127.8, 131.0, 132.1, 133.8, 133.9, 135.9, 141.0, 148.1 (12 × C_ar._), 151.5 (CH=N), 162.2, 168.8 (2 × C=O), 172.7, 175.8 (2 × C=O, thiazolidine). MS (APCI) [M]^+^ *m/z* = 462.0488. Anal. calc. for C_20_H_16_ClN_3_O_6_S (461.88) (%): C 52.01; H 3.49; N 9.10. Found: C 52.00; H 3.47; N 9.06.

##### 4-{[2-(2,4-Dichlorobenzoyl)hydrazinylidene]methyl}-2-methoxyphenyl (2,4-dioxo-1,3-thiazolidin-5-yl)acetate (17)

Yield 87%, m.p. 186–188 °C, ^1^H NMR *δ* ppm (DMSO-d_6_): 3.41–3.44 m (2H, CH–CH_2_); 3.84 s (3H, OCH_3_); 4.84–4.87 m (1H, C**H**–CH_2_); 7.20 d, 7.32 dd, 7.48–7.58 m, 7.64 d, 7.78 d (6H, Ar, *J*_1_=8.4 Hz, *J*_2_ = 1.8 Hz); 8.25 s (1H, CH=N); 12.01 s (1H, NHCO); 12.16 s (1H, NH, thiazolidine), ^13 ^C NMR *δ* ppm (DMSO-d_6_): 36.0 (CH_2_CH), 46.9 (CH_2_ CH), 56.4 (OCH_3_), 110.7, 121.1, 123.6, 128.0, 129.8, 131.2, 132.2, 133.6, 134.5, 135.7, 141.0, 148.0 (12 × C_ar._), 151.5 (CH=N), 162.2, 168.8 (2 × C=O), 172.7, 175.8 (2 × C=O, thiazolidine). MS (APCI) [M]^+^ m/*m/z* = 496.0087. Anal. calc. for C_20_H_15_Cl_2_N_3_O_6_S (496.32) (%): C 48.40; H 3.05; N 8.47. Found: C 48.37; H 3.03; N 8.44.

##### 2-{[2-(3-Chlorobenzoyl)hydrazinylidene]methyl}phenyl (2,4-dioxo-1,3-thiazolidin-5-ylidene)acetate (18)

Yield 88%, m.p. 243–245 °C, ^1^H NMR *δ* ppm (DMSO-d_6_): 7.16 s (1H, CH=); 7.34–7.46 m, 7.52–7.68 m, 7.84–7.99 m (8H, Ar); 8.51 s (1H, CH=N); 11.94 s (1H, NHCO); 12.95 s (1H, NH, thiazolidine), ^13 ^C NMR *δ* ppm (DMSO-d_6_): 116.5 (CH = C), 123.5, 126.6, 127.0, 127.5, 127.8, 131.0, 131.8, 132.2, 133.8, 134.3, 135.7, 143.1 (12 × C_ar._), 145.7 (CH=C), 148.9 (CH=N), 162.1, 164.3 (2 × C=O), 166.6, 169.3 (2 × C=O, thiazolidine). MS (APCI) [M]^+^ *m/z* = 430.0207. Anal. calc. for C_19_H_12_ClN_3_O_5_S (429.83) (%): C 53.09; H 2.81; N 9.78. Found: C 53.06; H 2.82; N 9.79.

##### 3-{[2-(3-Chlorobenzoyl)hydrazinylidene]methyl}phenyl (2,4-dioxo-1,3-thiazolidin-5-ylidene)acetate (19)

Yield 91%, m.p. 282–283 °C, ^1^H NMR *δ* ppm (DMSO-d_6_): 7.08 s (1H, CH=); 7.31–7.34 m, 7.54–7.70 m, 7.88–7.97 m (8H, Ar); 8.47 s (1H, CH=N); 12.05 s (1H, NHCO); 12.98 s (1H, NH, thiazolidine), ^13 ^C NMR *δ* ppm (DMSO-d_6_): 116.6 (CH = C), 120.0, 123.7, 124.0, 126.0, 127.0, 127.9, 130.7, 131.0, 132.2, 133.8, 135.7, 136.4 (12 × C_ar._), 147.5 (CH=C), 150.8 (CH=N), 162.3, 164.3 (2 × C=O), 166.9, 169.6 (2 × C=O, thiazolidine). MS (APCI) [M]^+^ *m/z* = 430.0212. Anal. calc. for C_19_H_12_ClN_3_O_5_S (429.83) (%): C 53.09; H 2.81; N 9.78. Found: C 53.05; H 2.77; N 9.74.

##### 4-{[2-(3-Chlorobenzoyl)hydrazinylidene]methyl}phenyl (2,4-dioxo-1,3-thiazolidin-5-ylidene)acetate (20)

Yield 93%, m.p. 278–279 °C, ^1^H NMR *δ* ppm (DMSO-d_6_): 7.08 s (1H, CH=); 7.37 d, 7.55–7.69 m, 7.82–7.91 m, 7.97 s (8H, Ar, *J* = 8.4 Hz); 8.48 s (1H, CH=N); 12.00 s (1H, NHCO); 12.98 s (1H, NH, thiazolidine), ^13 ^C NMR *δ* ppm (DMSO-d_6_): 116.7 (CH = C), 122.6, 127.0, 127.8, 128.9, 131.0, 132.1, 132.9, 133.8, 135.8, 147.7 (12 × C_ar._), 145.4 (CH=C), 151.7 (CH=N), 162.2, 164.1 (2 × C=O), 166.5, 169.4 (2 × C=O, thiazolidine). MS (APCI) [M]^+^ *m/z* = 430.0210. Anal. calc. for C_19_H_12_ClN_3_O_5_S (429.83) (%): C 53.09; H 2.81; N 9.78. Found: C 53.13; H 2.84; N 9.76.

##### 4-{[2-(3-Chlorobenzoyl)hydrazinylidene]methyl}-2-methoxyphenyl (2,4-dioxo-1,3-thiazolidin-5-ylidene)acetate (21)

Yield 93%, m.p. 246–248 °C, ^1^H NMR *δ* ppm (DMSO-d_6_): 3.86 s (3H, OCH_3_); 7.09 s (1H, CH=); 7.30–7.38 m, 7.52–7.70 m, 7.88–7.90 m, 7.97 s (7H, Ar); 8.47 s (1H, CH=N); 12.02 s (1H, NHCO); 12.98 s (1H, NH, thiazolidine), ^13 ^C NMR *δ* ppm (DMSO-d_6_): 56.5 (OCH_3_), 115.8 (CH = C), 110.5, 121.1, 123.6, 127.0, 127.8, 131.0, 132.1, 133.8, 134.2, 135.8, 140.6, 148.0 (12 × C_ar._), 146.2 (CH=C), 151.4 (CH=N), 162.2, 163.7 (2 × C=O), 166.6, 169.4 (2 × C=O, thiazolidine). MS (APCI) [M]^+^ *m/z* = 460.0306. Anal. calc. for C_20_H_14_ClN_3_O_6_S (459.86) (%): C 52.24; H 3.07; N 9.14. Found: C 52.22; H 3.02; N 9.10.

##### 3-{[2-(Phenylcarbamothioyl)hydrazinylidene]methyl}phenyl (2,4-dioxo-1,3-thiazolidin-5-yl)acetate (22)

Yield 86%, m.p. 209–211 °C, ^1^H NMR *δ* ppm (DMSO-d_6_): 3.42–3.45 m (2H, CH–C**H_2_**); 4.84–4.88 m (1H, C**H**–CH_2_); 7.17–7.25 m, 7.36–7.56 m, 7.74–7.78 m (9H, Ar); 8.16 s (1H, CH=N); 10.17 s, 11.89 s (NHCSNH); 12.16 s (1H, NH, thiazolidine), ^13 ^C NMR *δ* ppm (DMSO-d_6_): 36.4 (CH_2_CH), 46.8 (CH_2_ CH), 120.3, 123.8, 126.0, 126.4, 126.7, 128.6, 130.4, 136.3, 139.5, 142.2 (12 × C_ar._), 151.0 (CH=N), 169.6 (C=O), 172.7, 175.9 (2 × C=O, thiazolidine), 176.7 (C=S). MS (APCI), fragment ion (*m/z*): 355.0727; 336.0097; 277.0273; 179.0266; 157.9907; 136.0225; 94.0657. Anal. calc. for C_19_H_16_N_4_O_4_S_2_ (428.48) (%): C 53.26; H 3.76; N 13.08. Found: C 53.29; H 3.77; N 13.09.

##### 3-[{2-[(4-Methylphenyl)carbamothioyl]hydrazinylidene}methyl]phenyl (2,4-dioxo-1,3-thiazolidin-5-yl)acetate (23)

Yield 80%, m.p. 194–196 °C, ^1^H NMR *δ* ppm (DMSO-d_6_): 2.32 s (3H, CH_3_); 3.42–3.45 m (2H, CH–C**H_2_**); 4.83–4.87 m (1H, C**H**–CH_2_); 7.18 d, 7.39–7.51 m, 7.73–7.77 m (8H, Ar, *J* = 8.4 Hz); 8.15 s (1H, CH=N); 10.10 s, 11.84 s (NHCSNH); 12.16 s (1H, NH, thiazolidine), ^13 ^C NMR *δ* ppm (DMSO-d_6_): 21.1 (CH_3_), 36.4 (CH_2_CH), 46.8 (CH_2_ CH), 120.2, 123.7, 126.4, 126.6, 129.0, 130.4, 135.1, 136.3, 136.9, 142.0 (12 × C_ar._), 151.0 (CH=N), 169.6 (C=O), 172.7, 175.9 (2 × C=O, thiazolidine), 176.8 (C=S). MS (APCI) [M]^+^ *m/z* = 443.0791. Anal. calc. for C_20_H_18_N_4_O_4_S_2_ (442.51) (%): C 54.29; H 4.10; N 12.66. Found: C 54.23; H 3.95; N 12.63.

##### 4-[(2-Carbamothioylhydrazinylidene)methyl]phenyl (2,4-dioxo-1,3-thiazolidin-5-yl)acetate (24)

Yield 66%, m.p. 208–210 °C, ^1^H NMR *δ* ppm (DMSO-d_6_): 3.42–3.45 m (2H, CH–C**H_2_**); 4.84–4.88 m (1H, C**H**–CH_2_); 7.17 d, 7.88 d (4H, Ar, *J* = 8.7 Hz); 8.05 s (2H, NH_2_); 8.22 s (1H, CH=N); 11.46 s (NHCS); 12.16 s (1H, NH, thiazolidine), ^13 ^C NMR *δ* ppm (DMSO-d_6_): 36.4 (CH_2_CH), 46.8 (CH_2_ CH), 122.4, 129.0, 132.7, 141.6 (6 × C_ar._), 151.5 (CH=N), 169.4 (C=O), 172.7, 175.9 (2 × C=O, thiazolidine), 178.5 (C=S). MS (APCI) [M]^+^ *m/z* = 353.0346. Anal. calc. for C_13_H_12_N_4_O_4_S_2_ (352.39) (%): C 44.31; H 3.43; N 15.90. Found: C 44.27; H 3.38; N 15.86.

##### 4-{[2-(Phenylcarbamothioyl)hydrazinylidene]methyl}phenyl (2,4-dioxo-1,3-thiazolidin-5-yl)acetate (25)

Yield 84%, m.p. 159–162 °C, ^1^H NMR *δ* ppm (DMSO-d_6_): 3.43–3.46 m (2H, CH–C**H_2_**); 4.85–4.89 m (1H, C**H**–CH_2_); 7.19–7.24 m, 7.38 t, 7.56 d (7H, Ar, *J* = 7.5 Hz); 7.99 d (2H, 4-O–C_6_H_4_, *J* = 8.7 Hz); 8.17 s (1H, CH=N); 10.15 s, 11.86 s (NHCSNH); 12.16 s (1H, NH, thiazolidine), ^13 ^C NMR *δ* ppm (DMSO-d_6_): 36.5 (CH_2_CH), 46.8 (CH_2_ CH), 122.4, 125.9, 126.5, 128.5, 129.4, 132.5, 139.5, 142.3 (12 × C_ar._), 151.7 (CH=N), 169.4 (C=O), 172.7, 175.9 (2 × C=O, thiazolidine), 176.5 (C=S). MS (APCI), fragment ion (*m/z*): 338.3411; 284.2929; 257.2465; 149.0230. Anal. calc. for C_19_H_16_N_4_O_4_S_2_ (428.48) (%): C 53.26; H 3.76; N 13.08. Found: C 53.22; H 3.77; N 13.08.

##### 2-Methoxy-4-{[2-(phenylcarbamothioyl)hydrazinylidene]methyl}phenyl (2,4-dioxo-1,3-thiazolidin-5-yl)acetate (26)

Yield 88%, m.p. 182–184 °C, ^1^H NMR *δ* ppm (DMSO-d_6_): 3.40–3.43 m (2H, CH–C**H_2_**); 3.85 s (3H, OCH_3_); 4.83–4.87 m (1H, C**H**–CH_2_); 7.15–7.25 m, 7.36–7.47 m, 7.54–7.58 m, 7.67 d (8H, Ar, *J* = 1.8 Hz); 8.14 s (1H, CH=N); 10.12 s, 11.90 s (NHCSNH); 12.13 s (1H, NH, thiazolidine), ^13 ^C NMR *δ* ppm (DMSO-d_6_): 36.1 (CH_2_CH), 46.9 (CH_2_ CH), 56.7 (OCH_3_), 111.5, 121.4, 123.3, 126.0, 126.7, 128.6, 133.7, 139.6, 140.8, 142.6 (12 × C_ar._), 151.4 (CH=N), 168.8 (C=O), 172.7, 175.8 (2 × C=O, thiazolidine), 176.6 (C=S). MS (APCI) [M]^+^ *m/z* = 458.0958. Anal. calc. for C_20_H_18_N_4_O_5_S_2_ (458.51) (%): C 52.39; H 3.96; N 12.22. Found: C 52.40; H 3.95; N 12.21.

##### 4-{[2-(Phenylcarbamothioyl)hydrazinylidene]methyl}phenyl (2,4-dioxo-1,3-thiazolidin-5-ylidene)acetate (27)

Yield 84%, m.p. 199–202 °C, ^1^H NMR *δ* ppm (DMSO-d_6_): 7.06 s (1H, CH=); 7.19–7.41 m, 7.56 d (7H, Ar, *J* = 8.1 Hz); 8.02 d (2H, 4-O–C_6_H_4_, *J* = 8.7 Hz); 8.18 s (1H, CH=N); 10.16 s, 11.88 s (NHCSNH); 12.72 s (1H, NH, thiazolidine), ^13 ^C NMR *δ* ppm (DMSO-d_6_): 116.6 (CH = C), 122.3, 125.9, 126.5, 128.5, 129.4, 132.8, 139.5, 142.2 (12 × C_ar._), 145.6 (CH=C), 151.6 (CH=N), 164.2 (C=O), 166.8, 169.5 (2 × C=O, thiazolidine), 176.6 (C=S). MS (APCI), fragment ion (*m/z*): 353.0611; 333.9962; 275.0140; 155.9762; 136.0231; 94.0658. Anal. calc. for C_19_H_14_N_4_O_4_S_2_ (426.47) (%): C 53.51; H 3.31; N 13.14. Found: C 53.47; H 3.25; N 13.11.

##### 2-Methoxy-4-{[2-(phenylcarbamothioyl)hydrazinylidene]methyl}phenyl (2,4-dioxo-1,3-thiazolidin-5-ylidene)acetate (28)

Yield 93%, m.p. 186–187 °C, ^1^H NMR *δ* ppm (DMSO-d_6_): 3.86 s (3H, OCH_3_); 7.08 s (1H, CH=); 7.20–7.28 m, 7.39 t, 7.48–7.58 m, 7.71 d (8H, Ar, *J_1_*=7.5 Hz, *J_2_*=1.5 Hz); 8.17 s (1H, CH=N); 10.14 s, 11.92 s (NHCSNH); 12.94 s (1H, NH, thiazolidine), ^13 ^C NMR *δ* ppm (DMSO-d_6_): 56.7 (OCH_3_), 115.9 (CH = C), 111.5, 121.4, 123.4, 126.0, 126.7, 128.6, 134.1, 139.6, 140.5, 142.5 (12 × C_ar._), 146.0 (CH=C), 151.4 (CH=N), 163.7(C=O), 166.5, 169.4 (2 × C=O, thiazolidine), 176.6 (C=S). MS (APCI), fragment ion (*m/z*): 383.0639; 302.0948; 150.0544; 136.0216; 94.0655. Anal. calc. for C_20_H_16_N_4_O_5_S_2_ (456.49) (%): C 52.62; H 3.53; N 12.27. Found: C 52.63; H 3.54; N 12.30.

### Bioassays

#### Antiproliferative activity in vitro

The experiment was carried out using normal human skin fibroblasts (BJ) and tumour cell lines: human lung carcinoma (A549), human hepatocellular carcinoma (HepG2), and human breast adenocarcinoma (MCF-7). All cell lines were obtained from ATCC (American Type Culture Collection, England, UK) and cultured in the appropriate, recommended by ATCC, culture medium, additionally supplemented with 10% foetal bovine serum and antibiotics: 100 U/ml penicillin and 100 µg/ml streptomycin. The cells were maintained at 37 °C in a humidified atmosphere of 5% CO_2_ and 95% air. Antiproliferative activity of the new compounds was assessed by, commonly used for this purpose, colorimetric MTT assay. Cells with active mitochondrial dehydrogenases reduced intracellularly the tetrazolium dye into purple formazan, which is proportional to the number of viable cells. The cells were seeded in 96-multiwell plates in 100 μl of the appropriate complete culture medium at a concentration of 7 × 10^3^ cells/well in the case of BJ cells (normal fibroblasts), 1 × 10^4^ cells/well in the case of A549 cells (lung cancer), 1.5 × 10^4^ cells/well in the case of HepG2 cells (liver cancer), and 2 × 10^4^ cells/well in the case of MCF-7 cells (breast cancer) so as to obtain 60–70% confluent culture after 24 h of incubation at 37 °C – the cells are then in the log (logarithmic) phase, during a period of active proliferation, but still have an unoccupied space to divide. Afterwards, the culture medium was discarded and replaced with 100 µl of different concentrations of new tested compounds and reference antiproliferative agent: irinotecan. The stock solutions of the compounds were prepared in DMSO, and solvent control was being tested concurrently to exclude toxicity attributed to DMSO. Ten serial two-fold dilutions using culture medium were performed to prepare different concentrations of the tested compounds, and the highest tested concentration was 200 µg/ml. Untreated cells (maintained in the complete culture medium) served as a negative control. After exposing cells for 48 h to the tested derivatives, the MTT assay was performed as described earlier^25^. Three independent experiments (*n* = 3) were performed in a quadruplicate. The obtained data were analysed using GraphPad Prism 5, version 5.03 software (La Jolla, CA). The IC_50_ values were determined for tested compounds revealing antiproliferative activity. Moreover, safety index (SI) was calculated using the following formula:

$$\text{SI}={\text{IC}}_{\text{5}0}\;\text{normal cells }\left(\text{BJ}\right)/{\text{IC}}_{\text{5}0}\;\text{cancer cells}$$

#### Microbiological tests

Newly synthesised compounds’ antibacterial activities were tested *in vitro* against six aerobic bacteria reference strains: three Gram-positive (*Staphylococcus aureus* ATCC 25923, methicillin-resistant *S. aureus* MIKROBANK 14.001 reference strain from National Medicines Institute in Warsaw, and *Staphylococcus epidermidis* ATCC 12228) and three Gram-negative (*Escherichia coli* ATCC 25922, *Proteus mirabilis* ATCC 12453, and *Pseudomonas aeruginosa* ATCC 9027).

Bacterial cell suspensions equivalent to 0.5 McFarland (1.5 × 10^8^ CFU/ml) were prepared in 0.85% sterile saline. Tested compounds’ stock solutions (50 mg/ml) were dissolved in dimethyl sulphoxide (DMSO). DMSO final concentration did not inhibit bacteria growth.

Bacteria strain susceptibility to tested agents was determined by broth microdilution method. Broth microdilution method was carried out in accordance with Clinical and Laboratory Standards Institute recommendations and was used as reference method^26^. Mueller–Hinton broth containing two-fold dilutions of the tested substances (at concentrations ranging from 3.91 to 1000 µg/ml) was used to determine their minimal inhibitory concentration (MIC) which is the lowest concentration of an antibacterial that will inhibit the visible growth of a microorganism after 18 h of incubation). Mueller–Hinton broth is recommended as the medium of choice for susceptibility testing of commonly isolated, rapidly growing aerobic, or facultative organisms. Gentamicin (KRKA, Slovenia), an aminoglycoside antibiotic active against a wide range of human bacterial infections, mostly Gram-negative bacteria including *Pseudomonas*, *Proteus*, *Serratia*, and the Gram-positive *Staphylococcus*, was used as a control antibacterial agent (at concentration from 0.12 µg to 1000 µg).

Tested compounds two-fold dilutions in Mueller–Hinton broth without bacteria were blank control wells (incubated on equal conditions). Polystyrene trays containing 96 wells were filled with serial two-fold Mueller–Hinton broth dilutions of tested agents. Next, the broth was inoculated with 20 μl of 0.5 McFarland microbial suspension (previously diluted 1:10 by Mueller–Hinton broth). Total volume per each well was 200 μl. After incubation (at 35 °C for 18 h), bacterial cultures’ optical density was measured by microplate reader (BioTek Synergy H4 Hybrid Reader) at 600 nm. The MIC values were determined by the comparison of broth bacterial culture OD_600_ containing tested concentrations of examined substances to blank control wells.

## Results and discussions

### Chemistry

**Figure 1.**
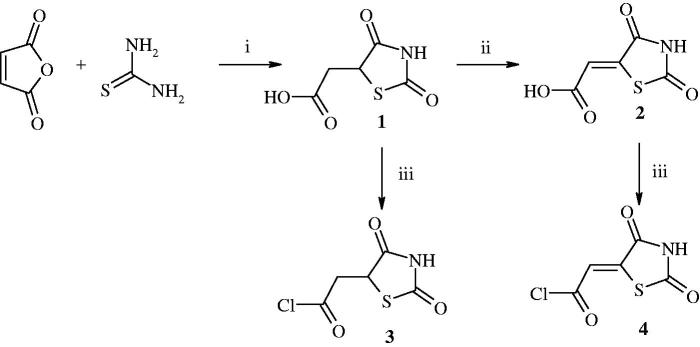
Synthesis of 2-(2,4-dioxothiazolidin-5-yl)acetyl chloride and 2-(2,4-dioxothiazolidin-5-ylidene)acetyl chloride. Reagent and conditions: (i) HCl, reflux; (ii) Br_2_, CH_3_COOH, reflux; (iii) SOCl_2_, DMF, 1,4-dioxane, reflux 1 h.

2-(2,4-Dioxothiazolidin-5-yl)acetic chloride^3^ and 2-(2,4-dioxothiazolidin-5-ylidene)acetic chloride^4^ were used as a starting material for the synthesis of target compounds. Those acetic chlorides^3^ ^,^ ^4^ were synthesised by the reaction of 2-(2,4-dioxothiazolidin-5-yl)acetic acid or 2-(2,4-dioxothiazolidin-5-ylidene)acetic acid with thionyl chloride in anhydrous 1,4-dioxane medium. In turn, 2-(2,4-dioxothiazolidin-5-yl)acetic acid^1^ was prepared from thiourea and maleic anhydride in the presence of concentrated hydrochloric acid^27^. 2-(2,4-dioxothiazolidin-5-ylidene)acetic acid^2^ was obtained by the reaction of compound **1** with bromine in the presence of acetic acid^28^. The reactions are shown in Figure 1.

**Figure 2.**
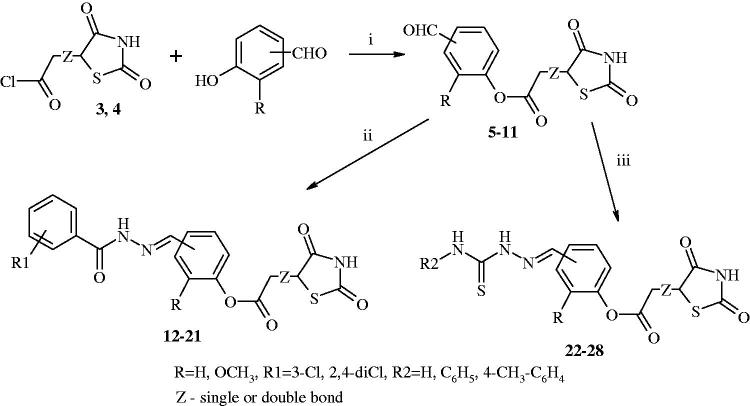
Synthesis of target compounds (**12–28**) 2-(2,4-dioxothiazolidin-5-yl)acetic and 2-(2,4-dioxothiazolidin-5-ylidene)acetic acid derivatives. Reagent and conditions: (i) pyridine, 1,4-dioxane, rt, after 2 h acidified of solution of hydrochloric acid; (ii) 3-chlorobenzhydrazide or 2,4-dichlorobenzhydrazide, anhydrous ethanol, reflux; (iii) corresponding 4-substituted thiosemicarbazide derivatives, anhydrous ethanol, reflux.

The next step of the synthesis was obtaining corresponding formylphenyl 2-(2,4-dioxothiazolidin-5-yl)acetate and formylphenyl 2-(2,4-dioxothiazolidin-5-ylidene)acetate^5–11^. Compounds **5–11** were prepared from acetic acid chlorides^3^ ^,^ ^4^ and a series of hydroxybenzaldehydes (namely salicylaldehyde, 3-hydroxybenzaldehyde, 4-hydroxybenzaldehyde, and vanillin). The reaction was provided in anhydrous 1,4-dioxane medium in the presence of anhydrous pyridine by previously described method^4^ ^,^ ^29^. Then, compounds **5–11** reacted with benzhydrazide derivatives (3-chlorobenzhydrazide and 2,4-dichlorobenzhydrazide), and thiosemicarbazide or 4-substituted-3-thiosemicarbazide derivatives (4-phenyl-3-thiosemicarbazide and 4-(4-methylphenyl)-3-thiosemicarbazide) in the presence of anhydrous ethanol. The result of this reaction was obtaining target compounds: hydrazones^12–21^ and thiosemicarbazones^22–28^. The synthesis of these compounds^12–28^ was achieved through an efficient synthetic route outlined in Figure 2.

The structure of the new derivatives was confirmed by an elemental analysis, ^1^H NMR and ^13 ^C NMR and MS spectra. In ^1^H NMR spectra, the protons of CH=N group of all the newly synthesised compounds showed a singlet signal at *δ* ∼ 8.14–8.51 ppm. The protons of NHCSNH group resonated as two singlets in the range of 10.10–10.17 ppm and 11.84–11.92 ppm correspondingly. The protons of NH group of the thiazolidine ring appeared in the 12.13–12.16 ppm region for compounds (**12**–**17** and **22**–**26**) and 12.72–12.98 ppm region for compounds as singlets^18–21^ ^,^ ^27^ ^,^ ^28^.

The presence of all carbon atoms for compounds^12–28^ was confirmed by ^13 ^C NMR spectra. For compounds **12–17** and **22**–**26** that are the derivatives of 2-(2,4-dioxothiazolidin-5-yl)acetic acid, carbon signal of two C=O group of the thiazolidine ring appeared in the 172.7 and 175.8–176.0 ppm regions. Signals of two C=O group of the thiazolidine ring for the 2-(2,4-dioxothiazolidin-5-ylilidene)acetic acid derivatives **18**–**21**, **27,** and **28** were visible at *δ*∼ 166.5–166.8 ppm and 169.3–169.6 ppm ranges, respectively. The detailed results of ^1^H NMR and ^13 ^C NMR spectra and MS are presented in the experimental part.

Molecular weight of compounds **12**–**21**, **23**, **24**, and **26** were confirmed by the mass spectra. In cases of compounds **22**, **25**, **27**, and **28,** a molecular ion was not observed in the mass spectra. The fragmentation was presented in the experimental part and ways of fragmentation were depicted in the supplementary material.

### Biology

#### Antiproliferative evaluation

Among all the new compounds tested, only **16**, **18**, **20**, **21**, **22**, and **23** derivatives revealed antiproliferative activity against tumour cell lines (Table 1). Compound **16** exhibited slight anticancer activity only against human breast adenocarcinoma cells (MCF-7). However, IC_50_ (concentration causing inhibition of cell proliferation by 50%) of compound **16** determined using normal human skin fibroblasts (BJ) was higher than IC_50_ of compound **16** estimated using MCF-7 cells, and the SI was relatively high and >1. Note that the higher SI (SI > 1) is, the safer compound is (less cytotoxic against normal cells). Also, agents revealing SI < 1 are considered unsafe and highly cytotoxic. The slight modification of the structure of compound **16** (changing a single bond in the position 5 of the thiazolidine-2,4-dione to the double bond) resulted in a new compound **21** that revealed broader antiproliferative activity, also against human hepatocellular carcinoma cells (HepG2). However, the SI value of compound **21** was low, indicating its high cytotoxicity against normal cells. Compound **20**, an analogue of the derivative **21** characterised by the lack of a methoxy group in a 4-formylphenyl, showed antiproliferative activity only against HepG2 cells. However, similar to compound **21**, the derivative **20** revealed low SI value. Compound **18**, characterised by the change in the configuration of formylphenyl (from 4-formyl-phenyl to 2-formyl-phenyl), appeared to be the most promising antiproliferative agent among all tested compounds. Compound **18** exhibited high antiproliferative activity against all tumour cell lines used in the experiment (lung, breast, and liver cancer) and high SI values (>1). Moreover, the derivative **18** showed meaningfully lower IC_50_ values when compared to the reference agent, irinotecan, which is an inhibitor of topoisomerase I and is commonly used in cancer treatment. Compound **18** revealed the highest antiproliferative activity against human breast adenocarcinoma cells (MCF-7). In the case of MCF-7 cells, the IC_50_ value of compound **18** was 13-fold lower (1.59 µg/ml) than IC_50_ value of irinotecan (21.08 µg/ml), whereas the SI value of compound **18** was three-fold higher when compared to the reference substance. In the case of human hepatocellular carcinoma (HepG2) and human lung carcinoma (A549) cells, the IC_50_ values of compound **18** were also very low: 3.86 µg/ml (four-fold lower compared to irinotecan) and 8.08 µg/ml (five-fold lower compared to irinotecan), respectively. Comparing the activity of compound **18** to the activity of structurally similar 2,4-dioxothiazolidine-5-acetic acid derivatives synthesised by Alegaon et al.^6^, it was observed that compound **18** showed higher activity against MCF-7 and A549 tumour cells. In their research, IC_50_ of tumour cell lines MCF-7 and A549 for the most active derivative 2-(2,4-dioxo-1,3-thiazolidin-5-yl)-N-[5-(3,4,5-trimethoxyphenyl)-1,3,4-thiadiazol-2-yl]acetamide was 15.28 and 12.7 µg/ml, respectively.

**Table 1.** Antiproliferative activity of novel thiazolidine-2,4-dione derivatives and irinotecan used as a reference substance.

	A549		HepG2		MCF-7		BJ
Cell line compound	IC_50_ [µg/ml] ± SEM	SI [IC_50_BJ/IC_50_]	IC_50_ [µg/ml] ± SEM	SI [IC_50_BJ/IC_50_]	IC_50_ [µg/ml] ± SEM	SI [IC_50_BJ/IC_50_]	IC_50_[µg/ml] ± SEM
**16**					117.2 ± 1.23	1.39	163.3 ± 1.34
**18**	8.08 ± 1.05	1.24	3.86 ± 1.07	2.60	1.59 ± 1.06	6.32	10.05 ± 1.18
**20**			26.96 ± 1.02	0.42			11.35 ± 1.05
**21**			48.39 ± 1.02	0.65	124 ± 1.63	0.25	31.42 ± 1.08
**22**	57.27 ± 1.19	1.43	48.33 ± 1.07	1.69	72.02 ± 1.10	1.13	81.62 ± 1.11
**23**	31.31 ± 1.66	0.80	15.18 ± 1.25	1.66	34.18 ± 1.12	0.74	25.16 ± 1.52
Irinotecan – reference drug	39.69 ± 1.20	0.97	14.79 ± 1.24	2.62	21.08 ± 1.23	1.84	38.69 ± 1.37

The derivatives of 3-formylphenyl 2-(2,4-dioxo-1,3-thiazolidin-5-yl)acetate with the 4-phenyl-3-thiosemicarbazide (compound **22**) and 4-(4-methylphenyl)-3-thiosemicarbazide structure (compound **23**) also exhibited antiproliferative activity against all three tumour cell lines (A549, HepG2, and MCF-7), but only IC_50_ of compound **23**, determined using A549 cells (lung cancer), was lower compared to the reference substance. However, the derivative **23** showed antiproliferative activity against liver cancer cells (HepG2) comparable to irinotecan and with relatively high SI value (1.66), indicating that this new compound has a great potential to be used as an anticancer drug. The IC_50_ values of compound **22**, determined using all three cell lines, were relatively high. Nevertheless, the SI values of compound **22** were >1, indicating its high safety.

#### Antibacterial evaluation

Antibacterial activity of the newly synthesised compounds^12–28^ *in vitro* was screened for the bacteria growth inhibition using broth microdilution method (Table 2). Reference strains of Gram-negative and Gram-positive bacterial species were used. According to our screening results, some of the tested compounds showed potential antibacterial activity against Gram-positive bacteria. Compounds **12, 13, 15, 17,** and **25** exhibited similar MIC values for *S. aureus* ATCC 25923, methicillin-resistant *S. aureus* MIKROBANK 14.001, and *S. epidermidis* ATCC 12228 strains. The MIC values ranged from 250 µg/ml to 500 µg/ml. Compound **27** inhibited all the tested staphylococci reference strains in the concentration of 125 µg/ml. Compound **23** showed antibacterial activity in the concentration of 125 µg/ml for *S. aureus* ATCC 25923, methicillin-resistant *S. aureus* MIKROBANK 14.001 strains and exhibited better antibacterial activity against *S. epidermidis* ATCC 12228 strain (MIC value was 62.5 µg/ml). The same MIC value (62.5 µg/ml) was estimated for compound **18** towards *S. aureus* ATCC 25923, while MIC value for methicillin-resistant *S. aureus* MIKROBANK 14.001 and *S. epidermidis* ATCC 12228 strains was 125 µg/ml which indicates a lower antibacterial activity of compound **18** in comparison with the activity in case of *S. aureus* ATCC 25923.

**Table 2.** Newly synthesised compounds’ influence on the Gram-positive bacterial strains growth – MIC (μg/ml) values.

	Minimal inhibitory concentration MIC (μg/ml)		
Compound	*Staphylococcus aureus* 25923	*Staphylococcus aureus* MIKROBANK 14.001	*Staphylococcus epidermidis* 12228
**12**	500	500	500
**13**	500	500	500
**15**	500	500	500
**17**	250	250	500
**18**	62.5	125	125
**23**	125	125	62.5
**25**	250	250	250
**27**	125	125	125
**28**	125	125	125

The new compounds have no inhibitory effect on Gram-negative bacterial strains, with the exception of compound **18** which inhibited *E. coli* ATCC 25922 growth in the concentration of 125 µg/ml (the value is not placed in the Table 2). MIC values obtained for gentamicin, an antibiotic active against Gram-negative bacteria and Gram-positive *Staphylococcus* spp., were from 0.12 to 2.0 µg/ml, based on our results.

Relying on the obtained results, it may be assumed that some of the modifications introduced to the thiazolidine-2,4-dione may result in the synthesis of new compounds with a broad biological activity spectrum *inter alia* anticancer and antibacterial properties. Such outcome was observed especially in the case of the change in the configuration of formylphenyl (from 4-formyl-phenyl to 2-formyl-phenyl) in compound **18** and introduction of the 4-(4-methylphenyl)-3-thiosemicarbazide structure into compound **23**. The above-mentioned compounds revealed anticancer as well as antibacterial activity. However, they showed relatively high MIC values; thus, their application just as antibacterial agents is limited. However, it should be noted that compounds **18** and **23** proved to possess very promising anticancer properties, so the extension of their activity towards antibacterial properties may be an additional advantage as cancer patients have a reduced immunity and are particularly sensitive to bacterial infections.

## Conclusion

Conducted research resulted in the synthesis of new thiazolidine-2,4-dione derivatives exhibiting antiproliferative activity. Among all tested derivatives, compound **18**, characterised by the change in the configuration of formylphenyl from 4-formylphenyl to 2-formylphenyl, revealed the highest antiproliferative activity against human lung, breast, and liver cancer cells. More importantly, the derivative **18** showed meaningfully lower IC_50_ values when compared to the reference substance, irinotecan, and relatively high SI values. Moreover, compound **18** is characterised by antibacterial activity against some Gram-positive and Gram-negative strains. It is a very important quality as cancer patients have reduced immunity and are particularly sensitive to all kinds of infections, bacterial infections in particular. Therefore, it may be implied that the novel compound **18** appears to be a very promising agent for anticancer treatment.

## Supplementary Material

IENZ_1387543_Supplementary_Material.pdf
