# Supplementary material for: Synthesis and in vitro antiproliferative and antibacterial activity of new thiazolidine-2,4-dione derivatives
Source: J Enzyme Inhib Med Chem. 2017 Nov 3;33(1):17–24. doi: 10.1080/14756366.2017.1387543 (PMC6010082; doi:10.1080/14756366.2017.1387543)
Supplement: IENZ_1387543_Supplementary_Material.pdf [file IENZ_A_1387543_SM7296.pdf]

Supplementary material for:

Synthesis and *in vitro* antiproliferative and antibacterial activity of new thiazolidine-2,4-dione derivatives

Nazar Trotsko[a], Agata Przekora[b], Justyna Zalewska[b], Grażyna Ginalska[b], Agata Paneth[a], Monika Wujec[a]

[a]*Department of Organic Chemistry, Faculty of Pharmacy, Medical University of Lublin, Chodźki 4A, 20-093 Lublin, Poland*

[b]*Department of Biochemistry and Biotechnology, Faculty of Pharmacy, Medical University of Lublin, Chodźki 1, 20-093 Lublin, Poland*

Contents:

1. ^{1}H NMR spectra for compounds (**12-28**) ……2-18
2. ^{13}C NMR spectra for compounds (**12-28**)……19-35
3. MS spectra for compounds (**12-28**)…………..36-52

^{1}H NMR spectra for compounds (12-28)

	ppm	Hz	Intensity
1	3.45	1035.8	162.0
2	2.50	751.7	785.7
3	12.03	3612.4	159.5
4	8.45	2537.3	188.4
5	7.97	2392.0	145.9
6	7.90	2371.5	78.1
7	7.87	2363.8	90.7
8	7.70	2311.6	55.9
9	7.67	2303.5	94.1
10	7.64	2294.0	65.9
11	7.61	2284.1	134.3
12	7.58	2276.0	143.9
13	7.56	2269.8	197.7
14	7.53	2261.7	124.1
15	7.51	2253.9	59.4
16	7.23	2171.0	74.5
17	7.20	2161.8	64.7
18	4.90	1471.1	63.0
19	4.88	1465.7	111.3
20	4.88	1464.5	88.3
21	4.86	1459.0	69.5
22	3.47	1043.1	120.9
23	3.47	1040.5	127.8
24	3.34	1002.0	2664.0
25	12.16	3650.9	65.8

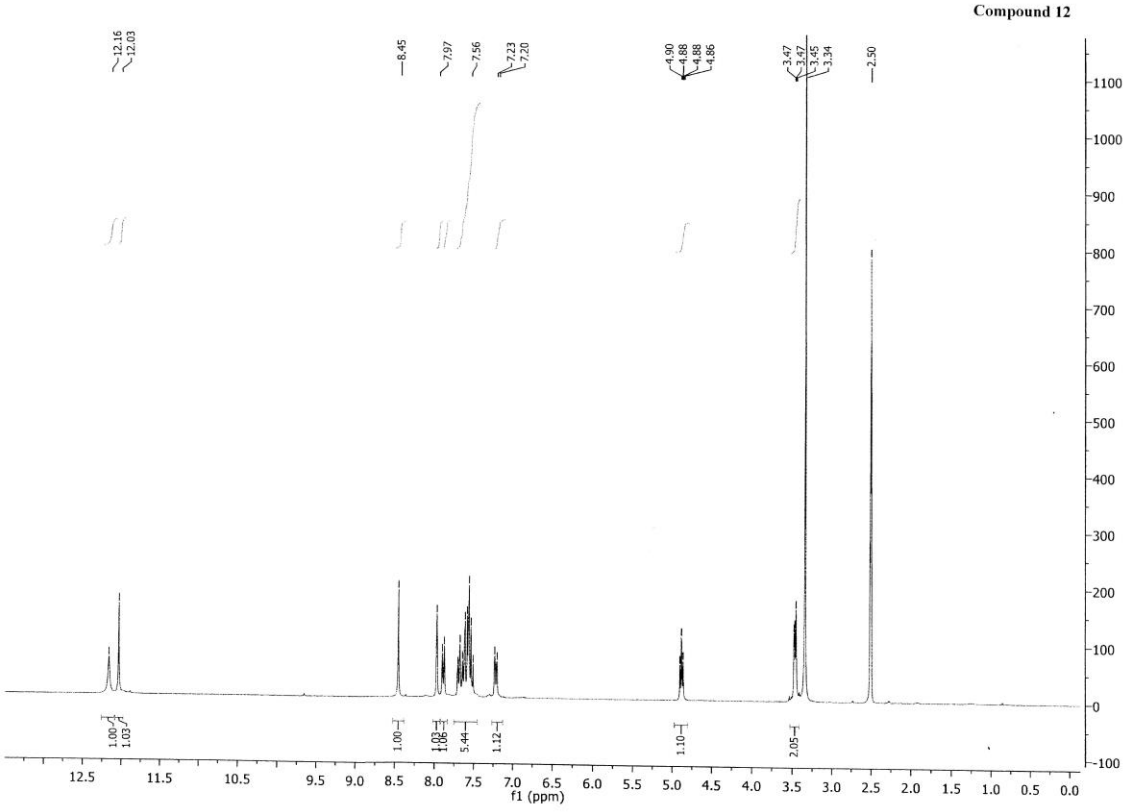

	ppm	Hz	Intensity
1	7.79	2336.9	184.7
2	3.44	1034.1	155.0
3	3.34	1002.1	1766.1
4	12.06	3618.9	170.5
5	8.27	2483.8	198.1
6	7.66	2298.2	106.2
7	12.15	3647.1	140.4
8	7.59	2278.0	146.7
9	7.56	2269.8	179.9
10	7.56	2267.9	184.4
11	7.53	2259.3	186.7
12	7.52	2256.8	178.5
13	7.24	2172.7	61.4
14	7.20	2161.5	47.1
15	4.90	1469.6	62.7
16	4.88	1464.1	119.8
17	4.85	1457.3	91.1
18	3.47	1041.3	117.8
19	3.46	1039.2	118.8
20	2.51	752.1	722.3
21	7.63	2290.0	228.6
22	7.79	2338.8	181.3

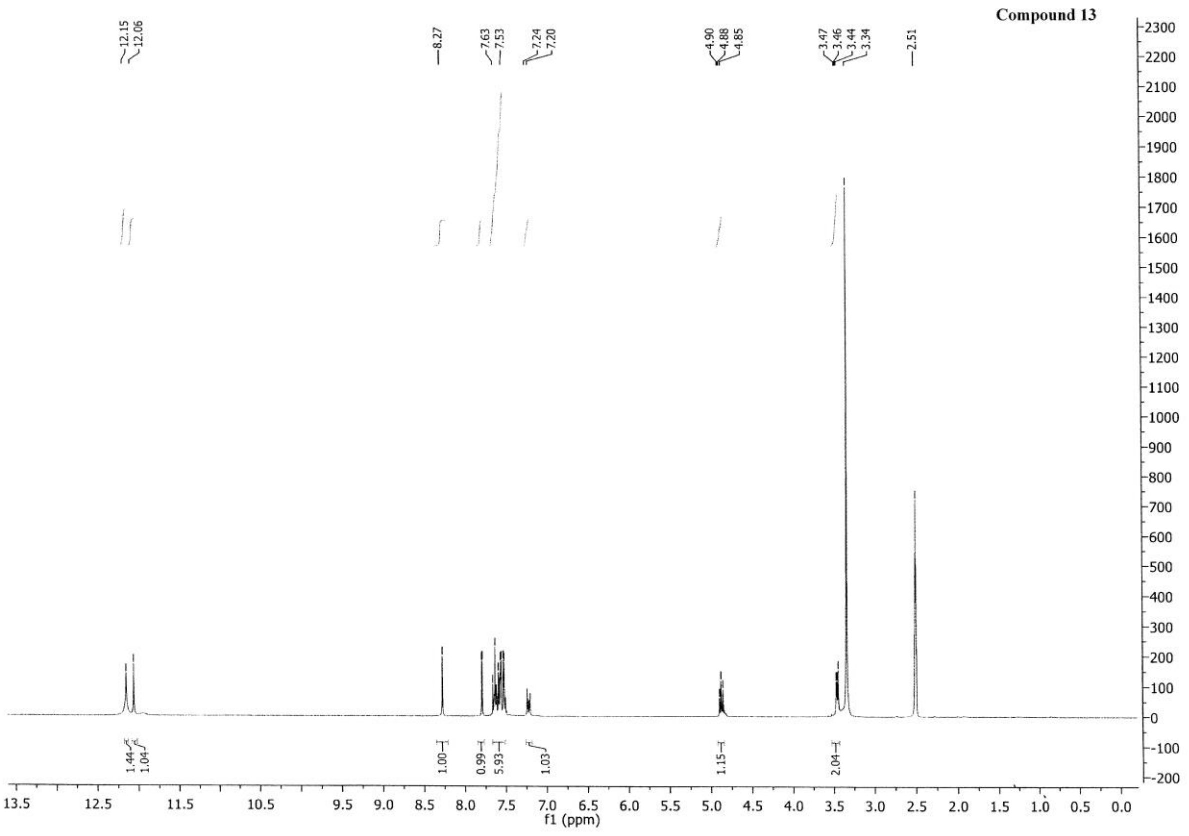

	ppm	Hz	Intensity
1	3.44	1032.3	305.6
2	2.51	752.1	1734.0
3	11.98	3595.8	389.5
4	8.46	2539.9	428.2
5	7.97	2392.5	328.7
6	7.90	2371.8	175.4
7	7.88	2364.1	206.2
8	7.83	2349.8	412.5
9	7.80	2341.2	454.8
10	7.70	2311.2	128.5
11	7.67	2303.1	210.2
12	7.61	2283.5	225.1
13	7.58	2275.7	290.4
14	7.55	2267.8	121.5
15	7.27	2181.7	463.7
16	7.24	2173.2	433.9
17	4.89	1468.3	151.5
18	4.87	1463.0	249.8
19	4.87	1461.5	201.9
20	4.85	1456.2	167.7
21	3.47	1041.1	247.1
22	3.46	1037.2	289.6
23	3.44	1034.1	316.0
24	3.34	1001.3	4134.0
25	12.16	3650.4	151.0

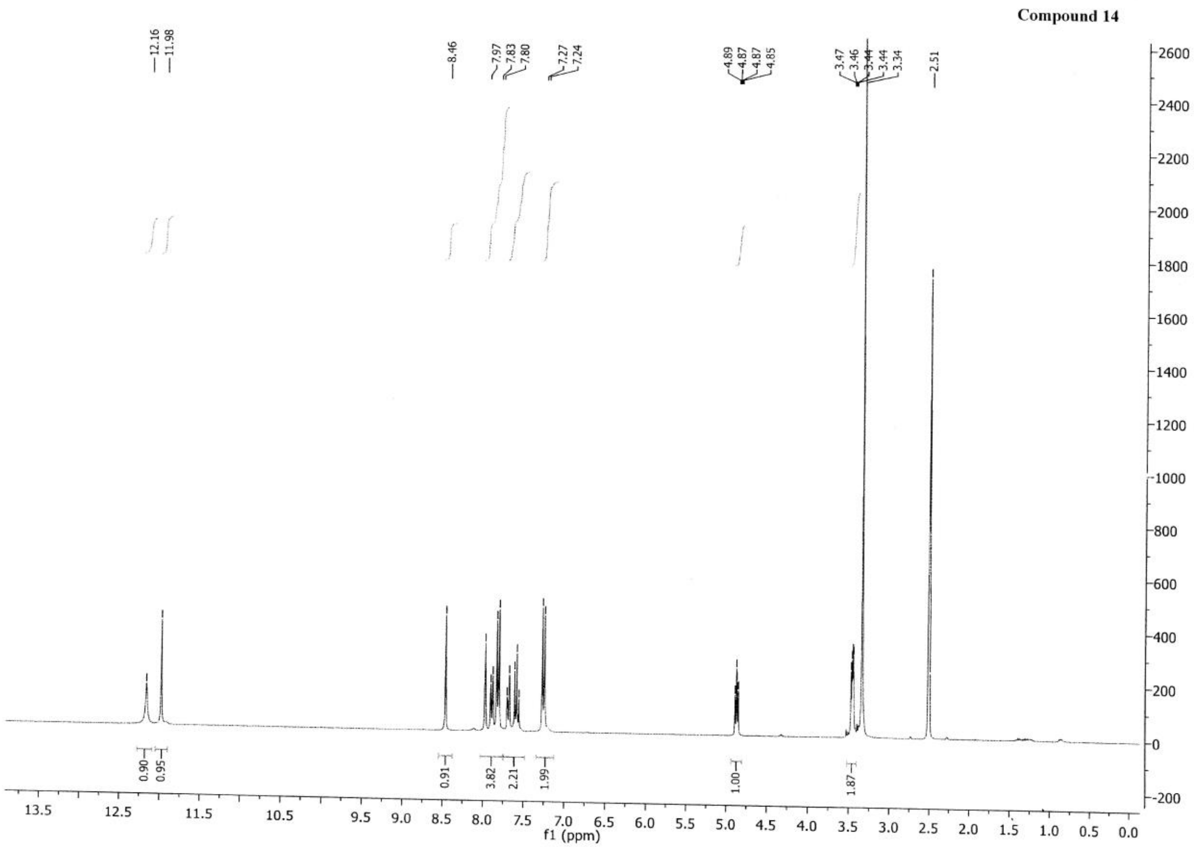

	ppm	Hz	Intensity
1	3.44	1032.4	359.2
2	2.51	752.2	830.1
3	12.00	3602.5	558.5
4	8.28	2485.9	588.4
5	7.82	2348.0	599.3
6	7.79	2339.3	684.9
7	7.78	2336.8	652.7
8	7.78	2334.8	589.9
9	7.66	2298.7	323.1
10	7.63	2290.4	633.3
11	7.58	2276.7	405.9
12	7.58	2274.8	360.9
13	7.56	2268.5	219.7
14	7.23	2171.6	627.6
15	4.86	1459.2	178.4
16	3.47	1041.9	299.4
17	7.55	2266.6	232.2
18	7.26	2180.3	675.0
19	4.89	1469.1	193.8
20	4.88	1463.9	336.1
21	4.87	1462.1	244.6
22	3.46	1037.5	347.5
23	3.45	1034.9	356.8
24	3.35	1005.3	1385.4
25	12.16	3649.1	264.0

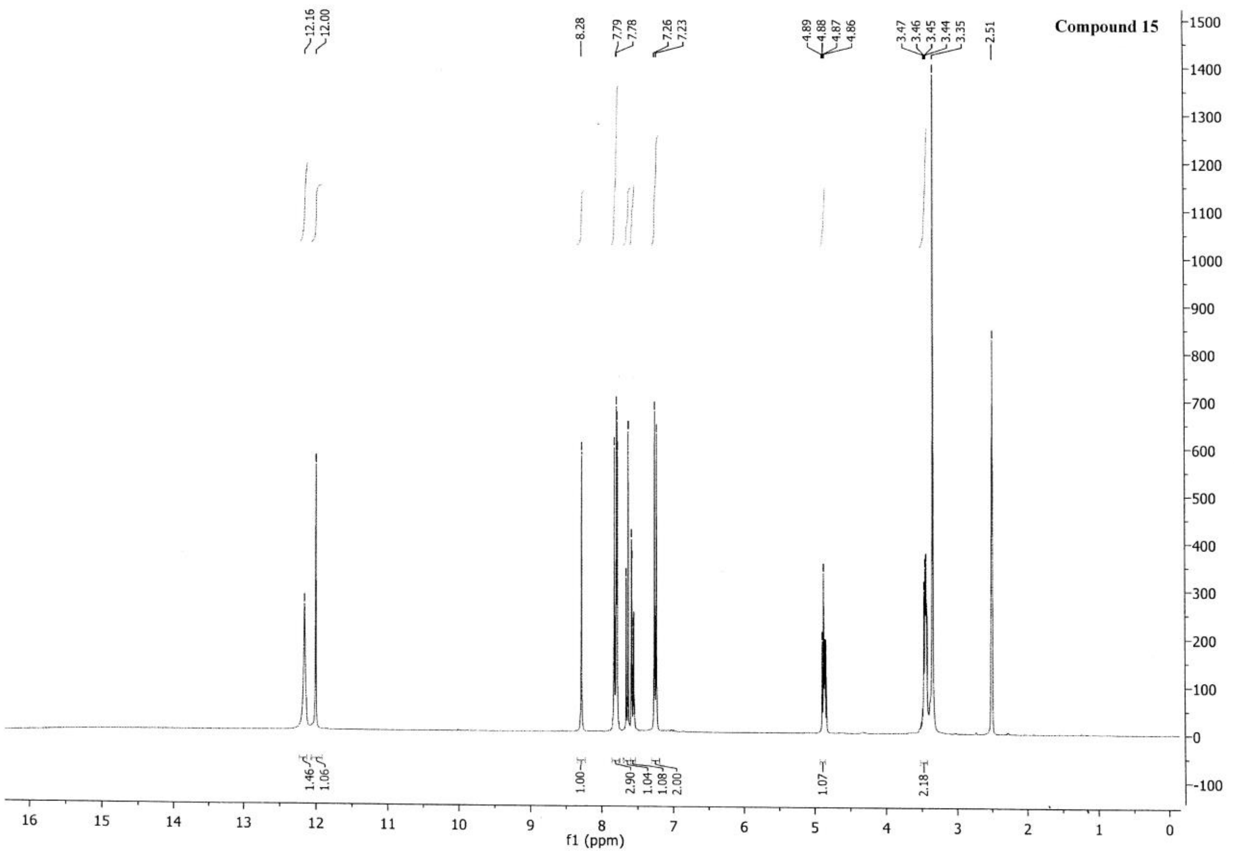

	ppm	Hz	Intensity
1	3.34	1003.1	894.7
2	4.84	1451.7	101.9
3	12.00	3601.5	272.3
4	8.44	2534.5	263.2
5	7.97	2391.1	203.4
6	7.90	2370.8	112.5
7	7.87	2363.0	125.1
8	7.70	2311.4	76.0
9	7.67	2302.6	124.0
10	7.61	2283.6	132.6
11	7.58	2275.8	172.7
12	7.56	2267.9	74.1
13	7.48	2245.8	202.8
14	7.34	2204.5	87.2
15	7.34	2203.0	83.5
16	7.32	2196.2	135.1
17	7.31	2194.8	131.6
18	7.23	2169.0	219.5
19	3.42	1026.7	254.7
20	4.88	1463.7	92.7
21	4.86	1458.3	169.4
22	12.14	3644.0	69.6
23	3.85	1156.1	913.6
24	3.44	1033.6	175.6
25	3.44	1031.8	189.0
26	2.50	751.7	636.2
27	7.20	2160.9	151.4
28	4.85	1457.2	132.6

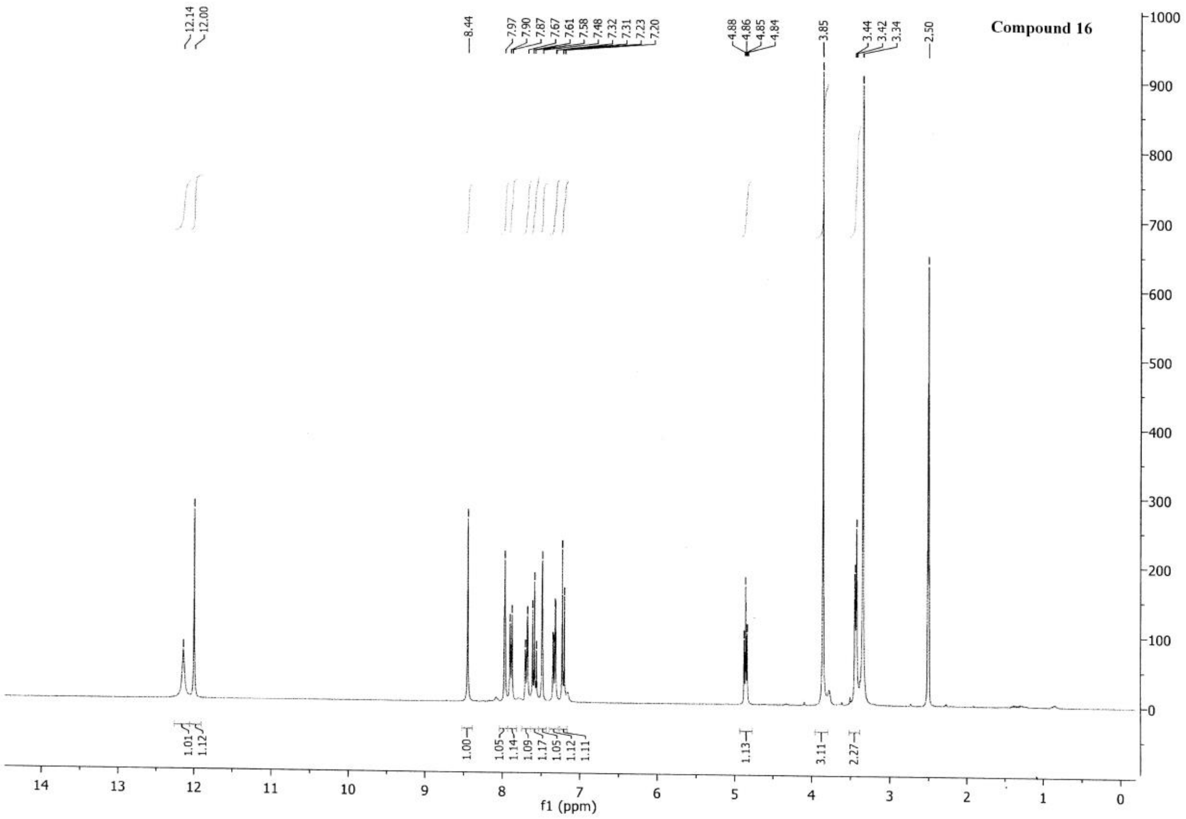

	ppm	Hz	Intensity
1	3.35	1005.3	884.7
2	4.80	1439.7	25.2
3	12.01	3606.0	110.8
4	8.25	2477.8	122.3
5	7.78	2336.0	120.3
6	7.78	2334.1	122.6
7	7.65	2296.3	64.6
8	7.62	2288.1	133.4
9	7.58	2275.9	87.8
10	7.55	2265.7	47.3
11	7.52	2257.0	149.1
12	7.49	2247.7	92.5
13	7.33	2200.2	37.3
14	7.30	2191.9	56.4
15	7.21	2165.1	115.6
16	3.39	1016.7	78.7
17	4.87	1461.6	39.0
18	4.85	1456.3	76.9
19	12.16	3650.9	67.8
20	3.84	1152.9	456.7
21	3.44	1031.6	70.7
22	3.43	1029.4	75.5
23	3.41	1024.5	101.3
24	2.50	750.4	285.3
25	7.19	2157.0	81.7
26	4.82	1446.2	49.2

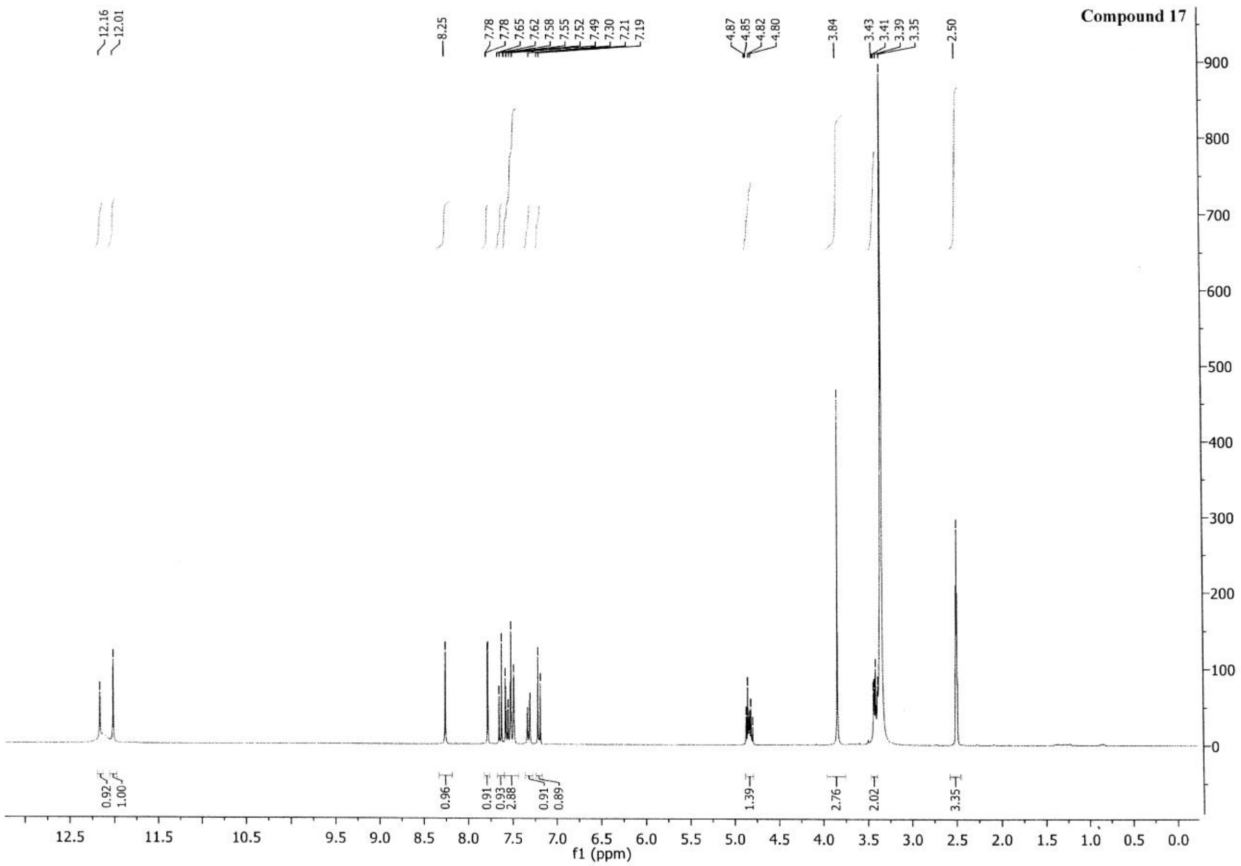

	ppm	Hz	Intensity
1	7.34	2204.8	132.5
2	3.38	1015.6	61.4
3	11.94	3585.3	183.5
4	8.51	2555.4	234.0
5	7.99	2398.2	101.2
6	7.96	2390.8	109.8
7	7.92	2378.4	186.4
8	7.86	2359.8	183.1
9	7.84	2351.9	140.6
10	7.68	2306.0	111.8
11	7.66	2299.1	365.1
12	7.36	2210.6	229.6
13	7.16	2150.2	302.9
14	7.59	2278.1	134.4
15	2.50	751.7	556.5
16	7.52	2258.0	92.5
17	7.56	2270.3	191.1
18	7.41	2225.5	57.5
19	7.25	2176.6	30.0
20	7.46	2240.3	85.3
21	7.44	2232.8	120.8
22	12.95	3887.7	8.7

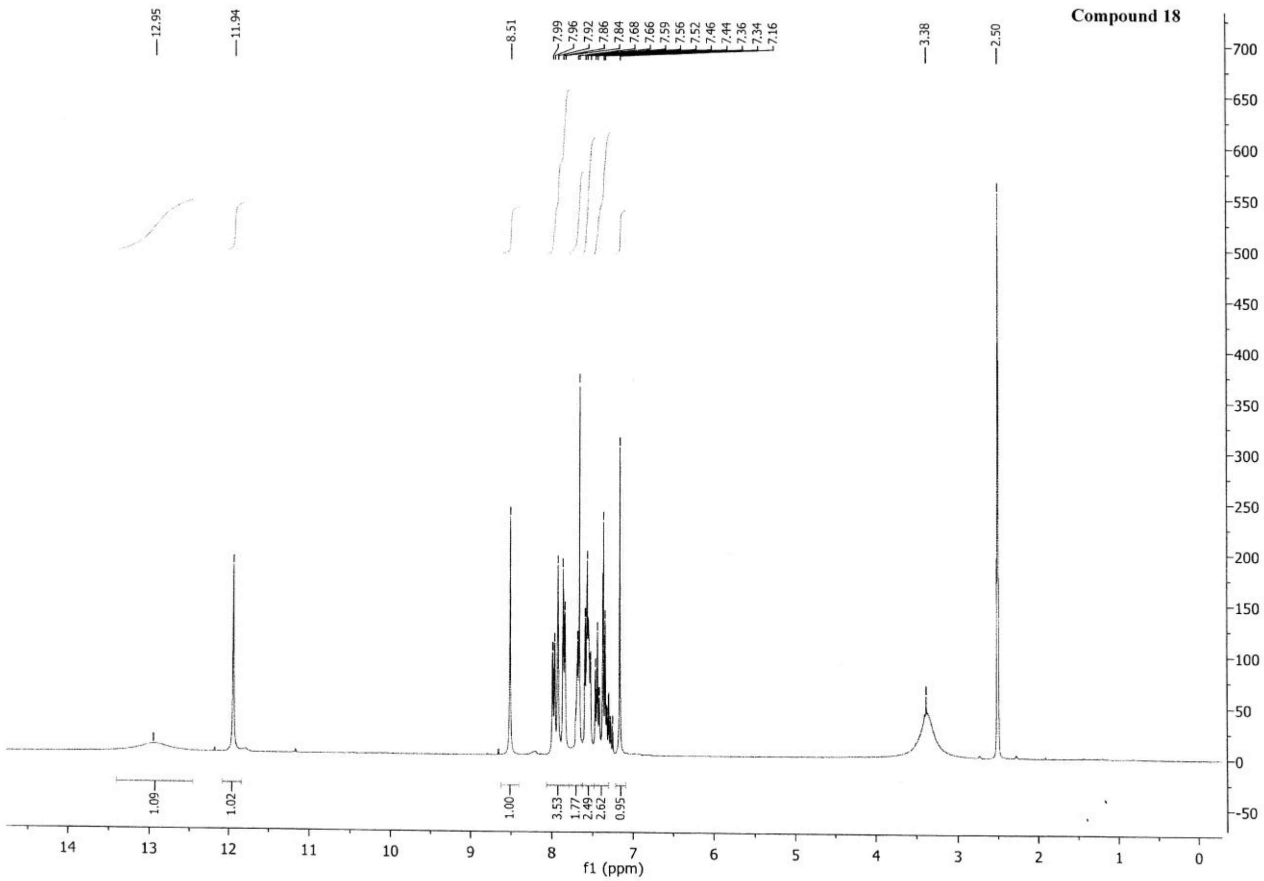

	ppm	Hz	Intensity
1	7.08	2124.3	789.8
2	2.51	752.1	2012.6
3	12.05	3617.7	332.4
4	8.47	2541.3	397.1
5	7.97	2392.9	306.1
6	7.90	2372.4	172.7
7	7.88	2364.7	201.7
8	7.70	2310.4	208.9
9	7.61	2284.1	216.0
10	7.58	2276.3	305.3
11	7.65	2295.9	295.7
12	7.56	2270.6	278.4
13	7.67	2302.5	337.7
14	7.59	2278.3	207.4
15	7.56	2268.7	175.0
16	7.54	2262.8	138.4
17	7.34	2204.8	164.4
18	7.31	2195.5	135.9
19	3.34	1003.1	423.6
20	12.98	3895.7	9.9

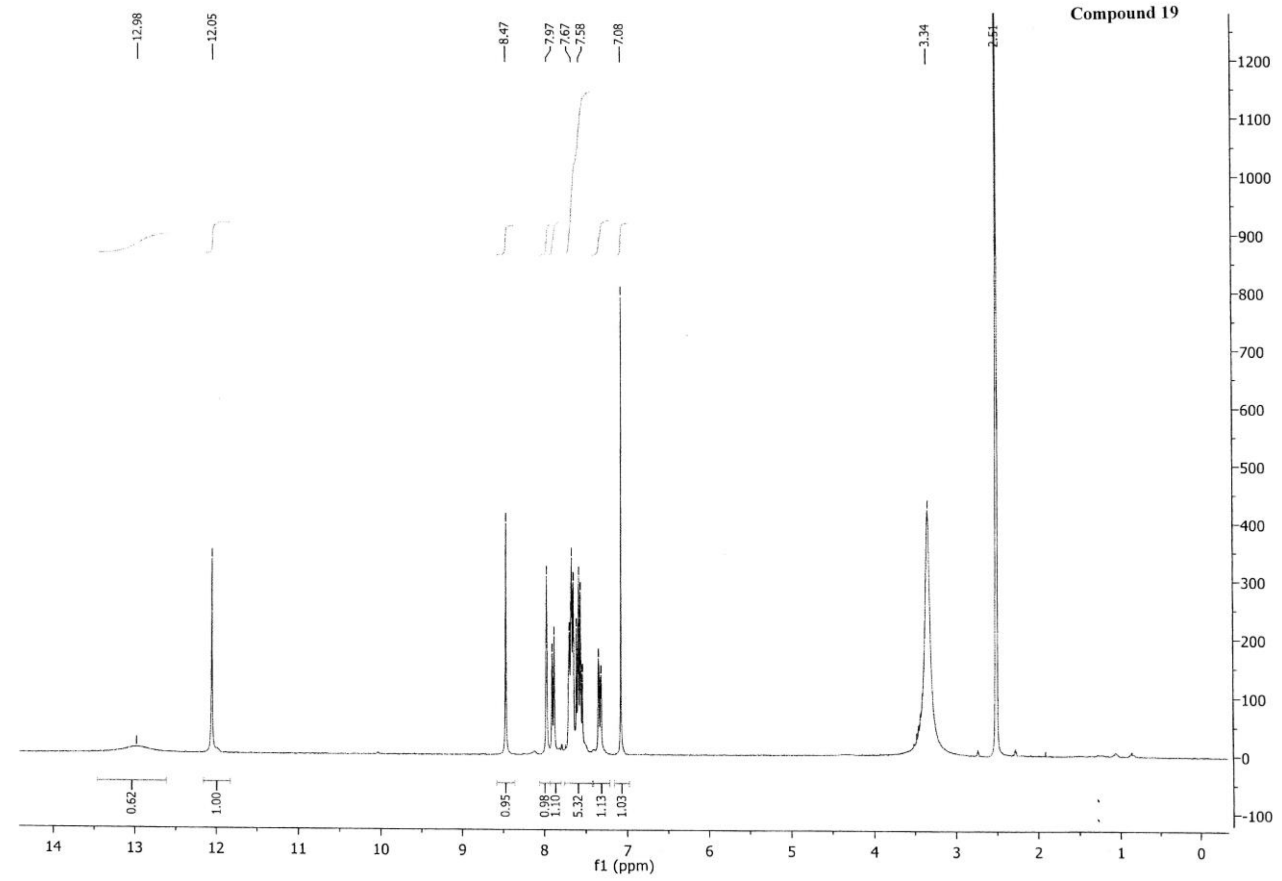

	ppm	Hz	Intensity
1	7.08	2123.9	695.1
2	2.51	752.0	470.1
3	12.00	3601.3	287.3
4	8.48	2545.1	324.0
5	7.97	2393.5	251.5
6	7.91	2373.0	135.2
7	7.88	2365.3	163.2
8	7.85	2357.0	313.1
9	7.82	2348.3	347.1
10	7.69	2309.6	95.4
11	7.67	2301.5	159.5
12	7.60	2282.8	175.7
13	7.58	2275.0	220.7
14	7.55	2267.1	95.9
15	7.38	2214.0	358.9
16	7.35	2205.4	333.1
17	3.37	1010.8	37.6
18	12.98	3896.9	9.3

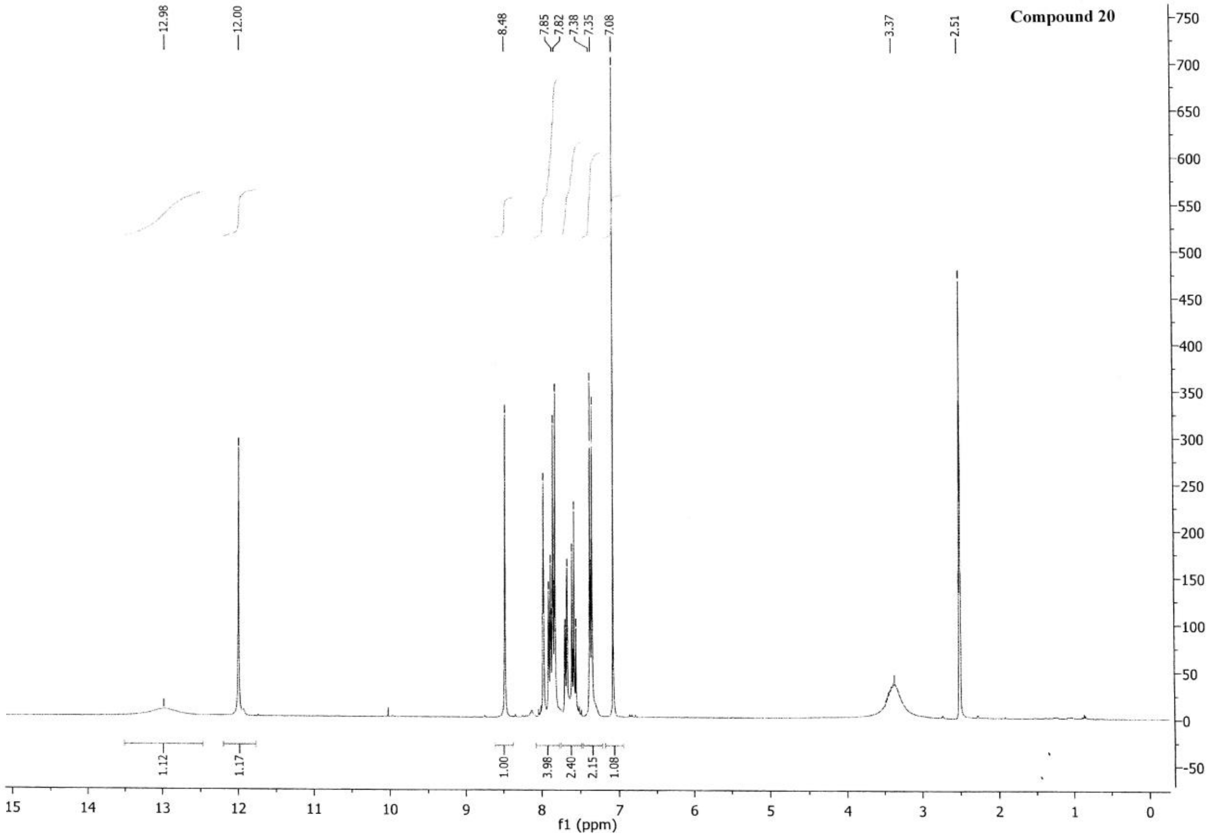

	ppm	Hz	Intensity
1	7.30	2192.1	177.2
2	3.86	1159.7	1848.6
3	12.02	3608.0	573.2
4	8.47	2541.2	554.4
5	7.97	2392.9	430.3
6	7.90	2372.7	237.9
7	7.88	2365.0	266.6
8	7.70	2312.4	163.4
9	7.68	2304.3	271.2
10	7.61	2284.9	274.9
11	7.59	2277.1	355.1
12	7.56	2269.2	159.0
13	7.52	2258.2	451.1
14	7.38	2214.6	119.3
15	7.35	2206.2	390.0
16	7.09	2127.6	1029.7
17	2.51	752.1	1323.8
18	3.36	1009.3	192.3
19	7.33	2200.3	575.1
20	12.98	3897.8	14.1

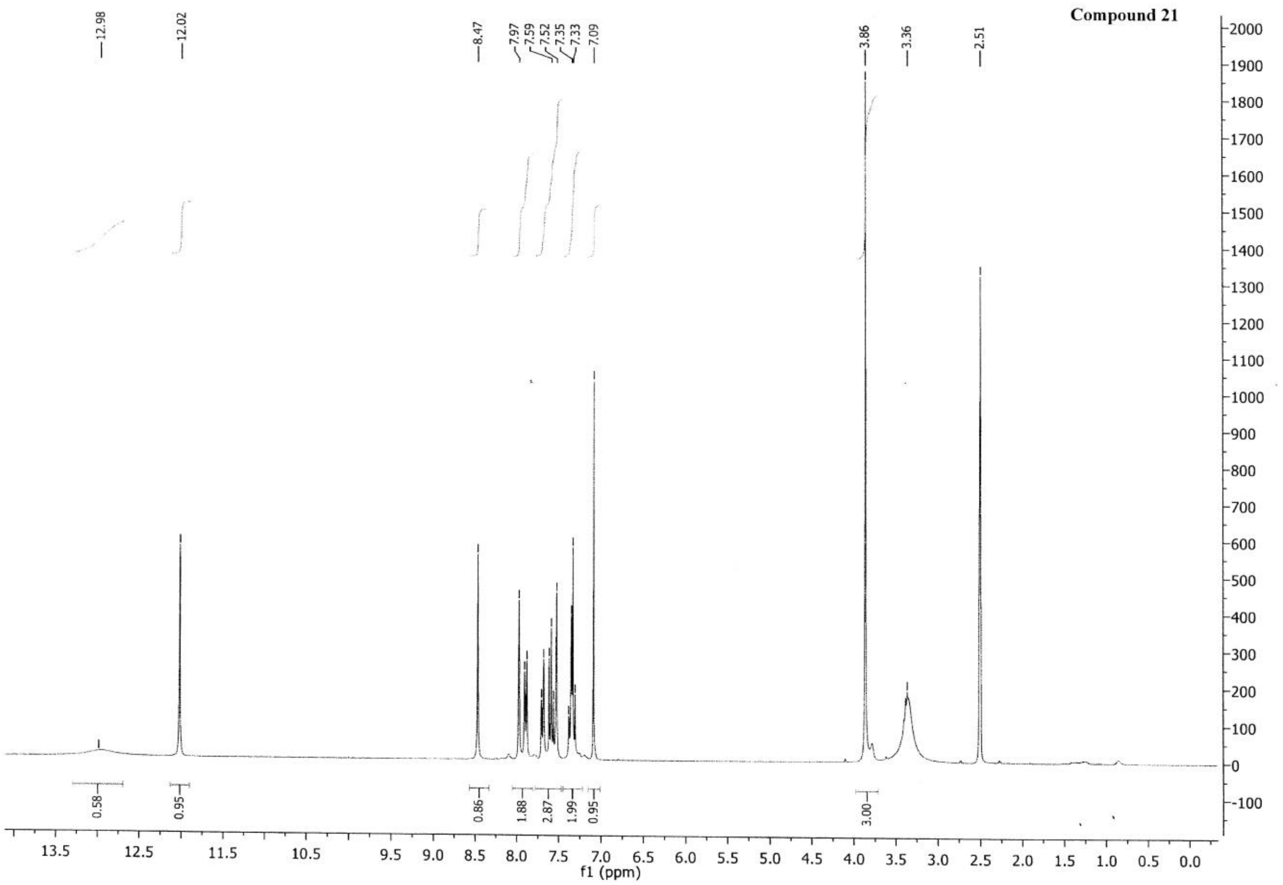

	ppm	Hz	Intensity
1	3.35	1004.8	829.9
2	8.16	2450.3	420.3
3	3.42	1028.1	265.7
4	12.16	3650.3	92.9
5	7.78	2334.7	277.4
6	7.77	2332.8	371.3
7	7.74	2324.0	189.0
8	7.56	2270.1	317.9
9	7.54	2262.6	398.2
10	7.52	2256.0	159.8
11	7.49	2248.1	262.8
12	7.46	2240.3	140.1
13	7.41	2224.4	237.6
14	7.39	2216.8	405.7
15	7.36	2208.8	247.1
16	7.25	2176.2	153.6
17	7.23	2168.8	225.1
18	7.20	2162.5	204.5
19	7.20	2160.3	182.0
20	4.88	1463.7	140.2
21	4.85	1456.9	167.2
22	7.17	2152.2	110.9
23	4.84	1451.6	147.0
24	7.18	2155.4	122.2
25	4.86	1458.4	263.3
26	3.45	1036.4	229.8
27	3.44	1033.2	237.9
28	3.43	1029.5	263.3
29	2.51	752.1	421.9
30	11.89	3570.3	346.7
31	10.17	3053.3	333.1

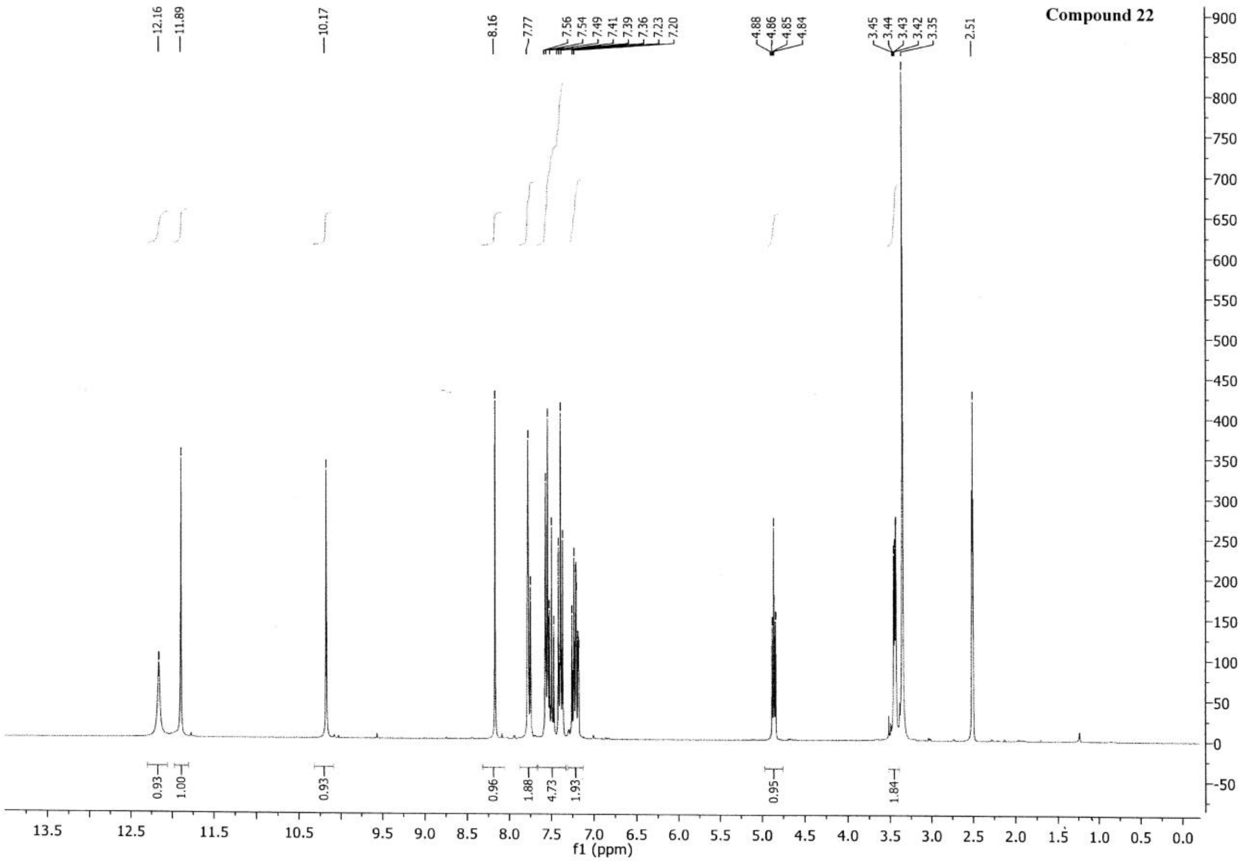

	ppm	Hz	Intensity
1	3.35	1004.6	700.2
2	2.32	696.1	1151.6
3	11.84	3554.6	366.1
4	10.10	3031.7	342.7
5	8.15	2446.5	434.8
6	7.77	2332.5	294.2
7	7.76	2330.5	390.8
8	7.73	2321.8	195.8
9	7.51	2254.5	160.7
10	7.48	2246.5	279.2
11	7.46	2238.7	150.4
12	7.42	2226.8	408.9
13	7.39	2218.5	522.2
14	4.87	1463.1	155.9
15	4.85	1456.3	178.3
16	7.17	2151.5	461.3
17	4.83	1451.1	160.7
18	7.19	2159.7	564.7
19	4.86	1457.9	304.8
20	3.45	1035.7	240.8
21	3.44	1032.5	249.7
22	3.43	1028.8	273.6
23	3.42	1027.3	271.5
24	2.50	751.9	458.7
25	12.16	3651.3	72.3

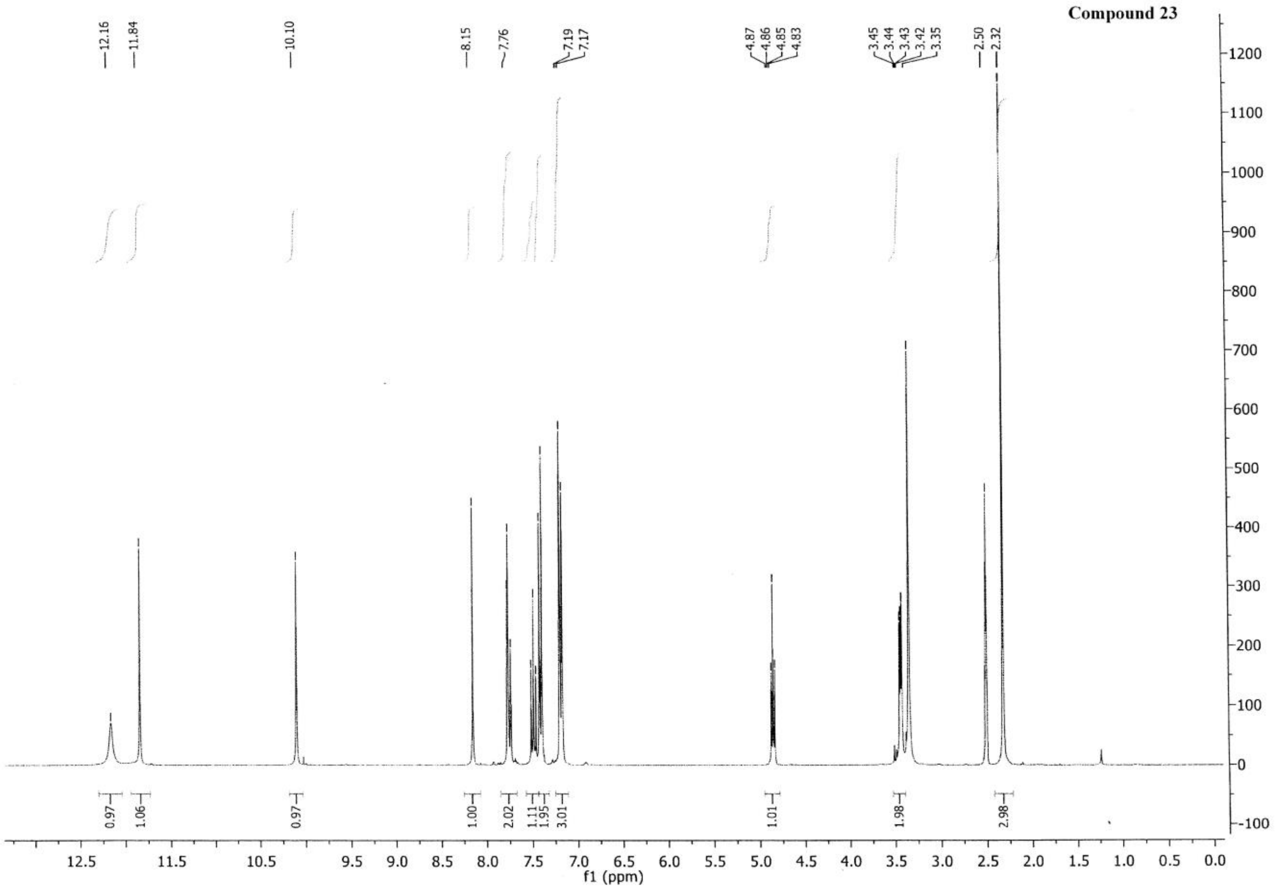

	ppm	Hz	Intensity
1	3.42	1025.8	555.7
2	8.05	2415.9	1166.7
3	2.51	752.3	1233.6
4	7.89	2368.4	990.0
5	3.34	1002.5	2108.2
6	8.22	2468.2	303.8
7	7.86	2359.7	1063.7
8	7.19	2157.5	1104.4
9	7.16	2148.8	1052.6
10	4.88	1465.4	313.9
11	4.86	1460.2	540.0
12	4.84	1453.3	337.5
13	3.45	1035.1	506.4
14	3.43	1030.9	575.2
15	11.46	3440.7	667.3
16	12.16	3649.6	228.2

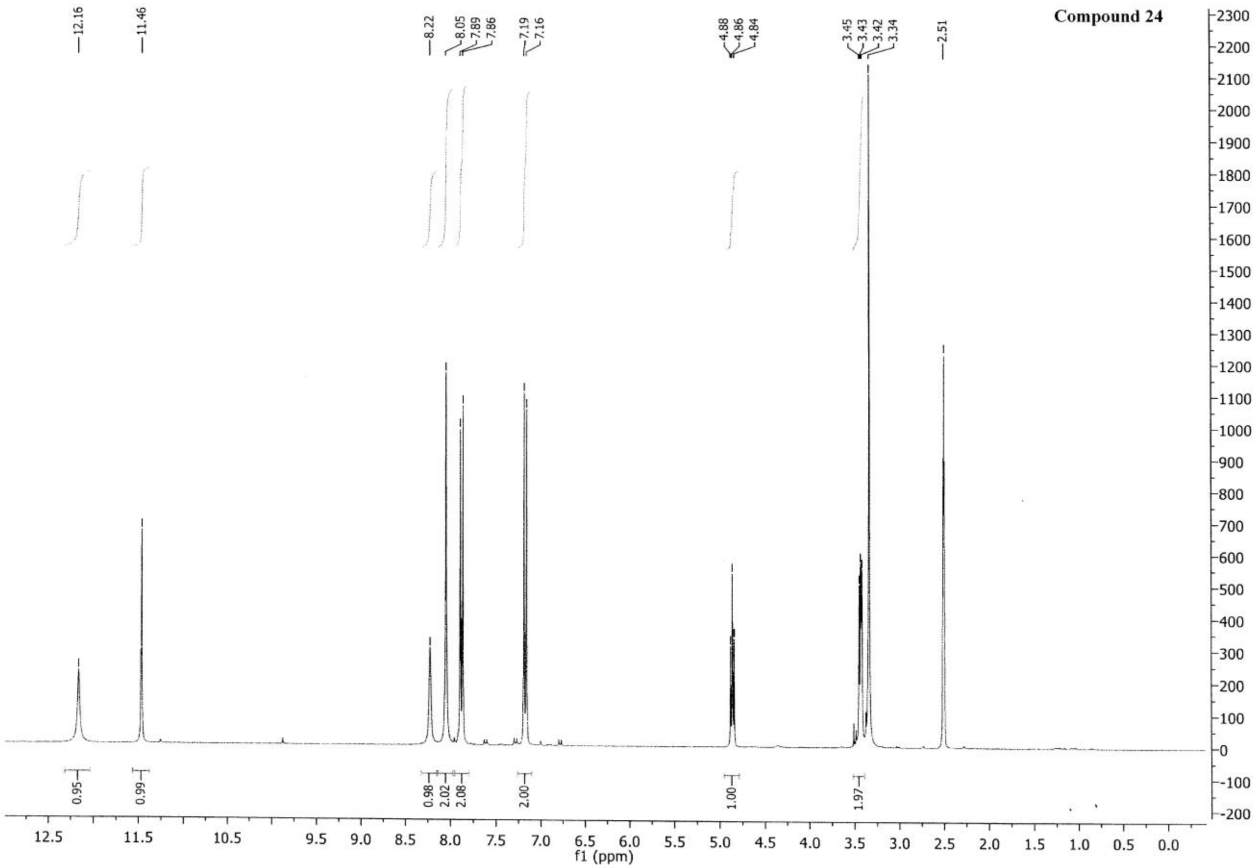

	ppm	Hz	Intensity
1	4.86	1460.0	185.4
2	2.50	751.9	369.0
3	11.86	3561.1	372.8
4	10.15	3046.2	307.6
5	8.17	2451.6	414.1
6	8.00	2402.3	470.2
7	7.97	2393.5	496.8
8	7.22	2167.0	703.1
9	7.24	2173.8	177.7
10	3.44	1033.5	256.4
11	7.58	2275.4	255.6
12	7.38	2215.5	436.8
13	7.35	2207.5	274.7
14	7.55	2266.7	409.1
15	3.35	1005.2	621.5
16	3.43	1028.3	250.8
17	7.41	2223.1	258.8
18	3.46	1037.6	232.0
19	7.58	2274.1	342.4
20	4.87	1461.8	300.6
21	7.19	2158.3	557.9
22	3.43	1030.6	260.8
23	4.85	1454.8	167.0
24	4.89	1467.0	155.6
25	12.16	3651.3	100.7

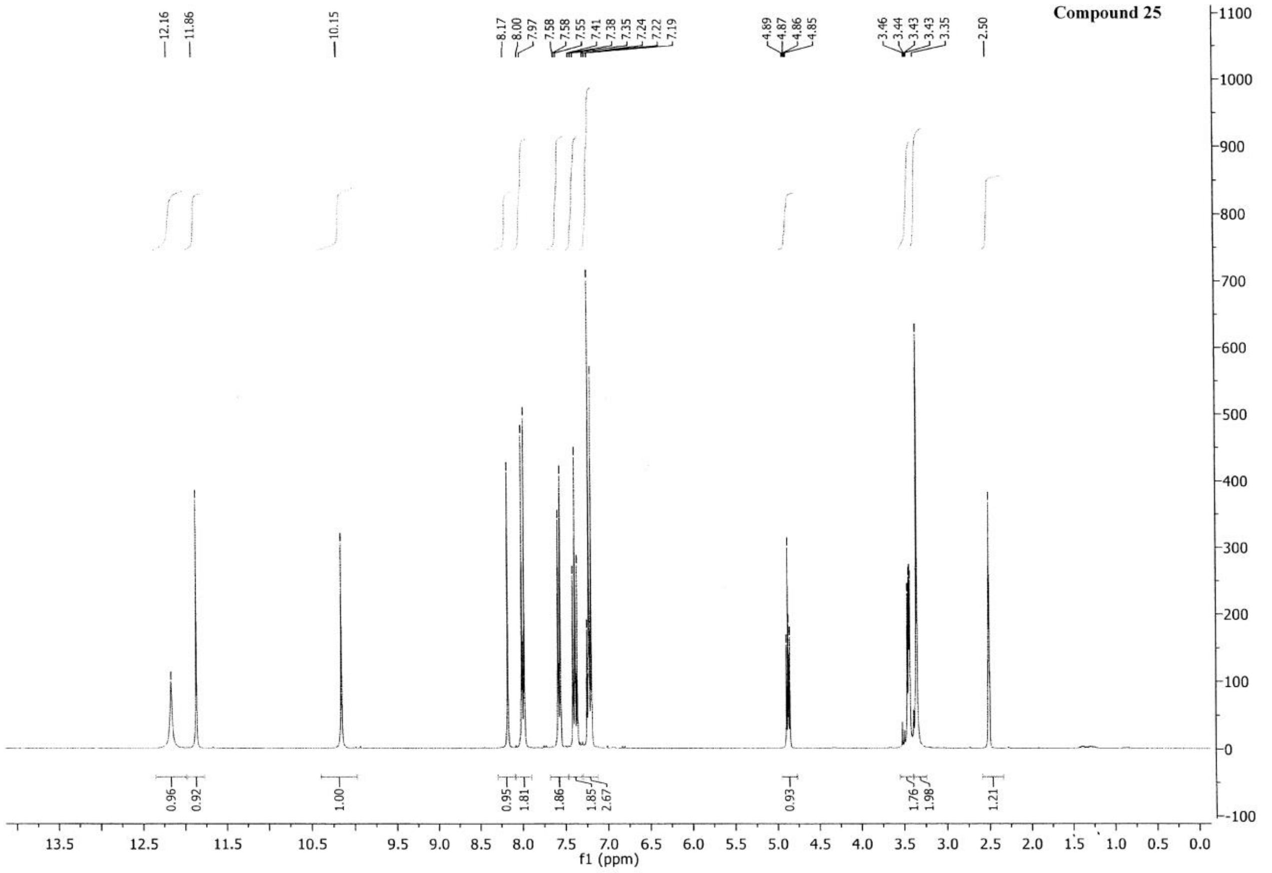

	ppm	Hz	Intensity
1	3.40	1021.8	223.7
2	2.50	751.7	702.2
3	11.90	3570.8	214.6
4	10.12	3037.9	184.2
5	8.14	2444.7	248.6
6	7.67	2303.5	190.5
7	7.67	2301.7	192.4
8	7.57	2272.7	191.6
9	7.55	2265.3	236.7
10	7.54	2264.6	222.5
11	7.47	2243.7	101.2
12	7.47	2242.0	94.2
13	7.45	2235.4	116.2
14	7.44	2233.7	112.0
15	7.41	2224.7	147.0
16	7.39	2217.2	251.5
17	7.36	2209.1	154.3
18	7.25	2176.7	94.3
19	7.23	2169.4	133.6
20	7.20	2162.0	53.6
21	7.17	2153.2	244.2
22	7.15	2145.0	218.2
23	4.87	1461.2	86.1
24	4.85	1455.6	185.7
25	4.83	1449.1	92.3
26	3.85	1155.4	1035.8
27	3.43	1028.6	161.1
28	3.34	1001.9	1898.0
29	12.13	3642.2	48.8

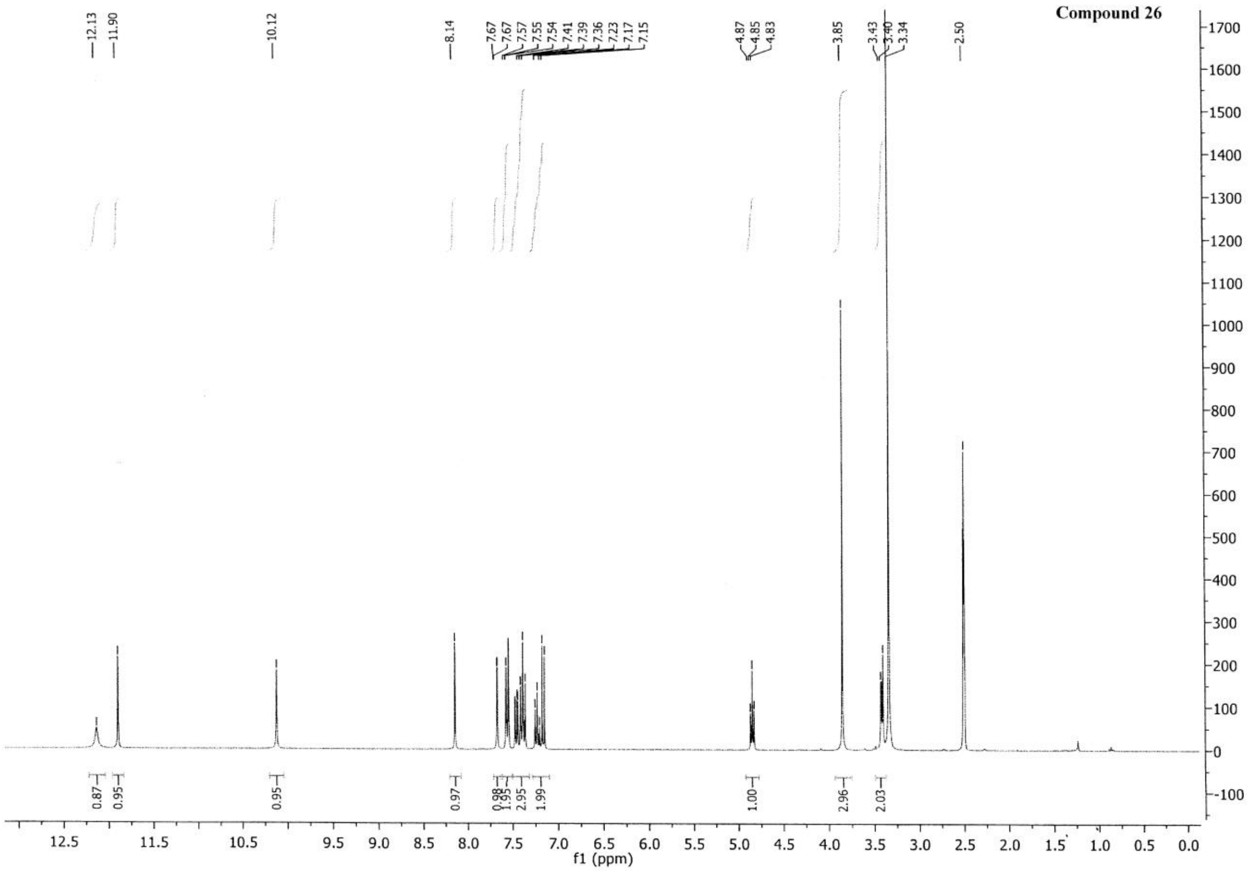

	ppm	Hz	Intensity
1	7.06	2120.0	757.8
2	2.50	751.8	850.6
3	11.88	3564.9	252.7
4	10.16	3050.8	202.7
5	8.18	2454.8	280.2
6	8.03	2410.5	306.5
7	8.00	2401.7	330.2
8	7.57	2273.4	234.2
9	7.55	2265.3	265.8
10	7.41	2223.0	170.1
11	7.38	2215.5	291.5
12	7.35	2207.4	189.0
13	7.33	2200.3	364.6
14	7.30	2191.6	356.7
15	7.24	2173.9	108.4
16	7.22	2166.5	157.3
17	7.19	2159.2	63.6
18	3.34	1003.6	224.8
19	12.72	3819.2	2.8

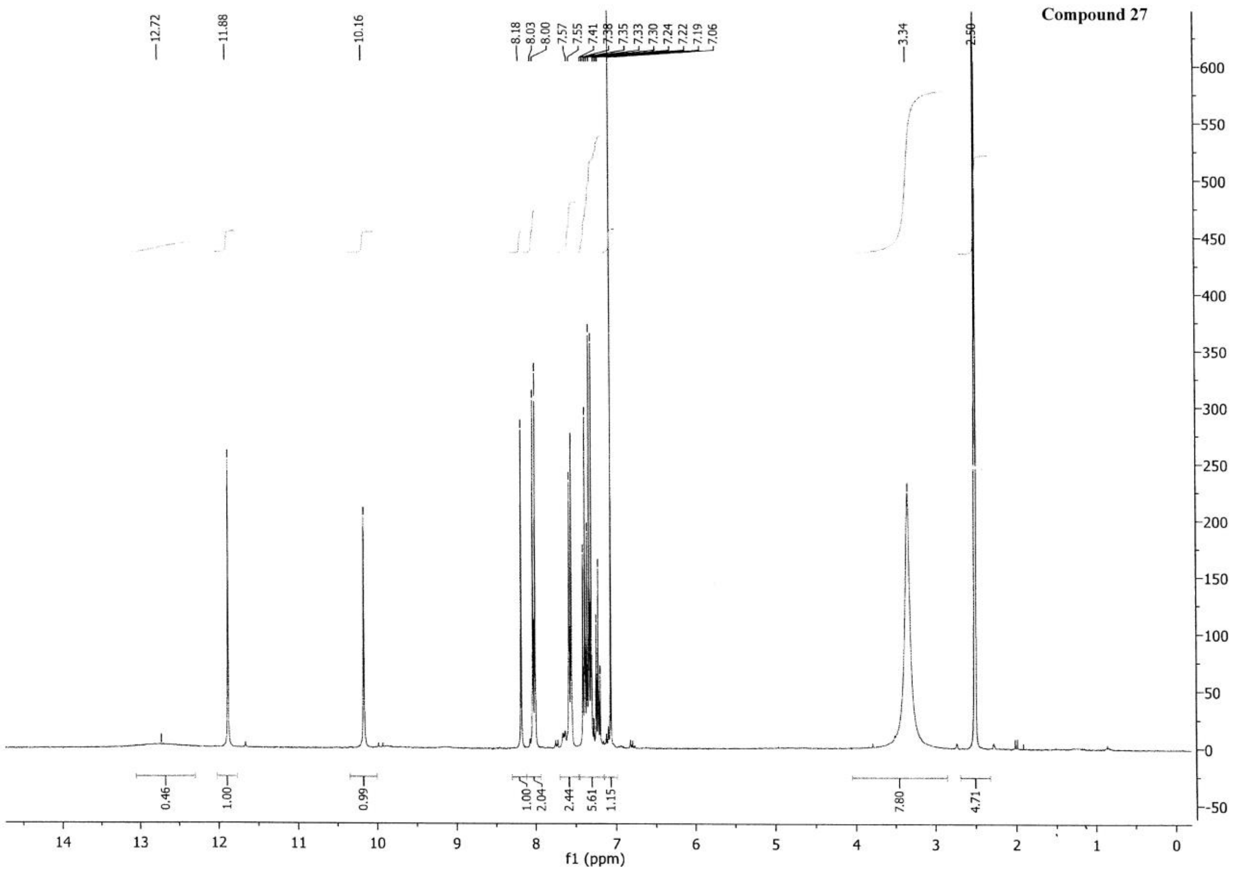

	ppm	Hz	Intensity
1	3.86	1158.9	1647.6
2	2.51	752.1	660.1
3	11.92	3577.5	355.1
4	10.14	3042.9	299.7
5	8.17	2451.0	402.6
6	7.71	2313.9	310.7
7	7.70	2312.2	312.5
8	7.58	2274.4	319.0
9	7.55	2266.9	393.1
10	7.51	2254.7	150.4
11	7.48	2246.4	182.5
12	7.41	2225.6	241.2
13	7.39	2218.1	413.6
14	7.36	2210.0	254.8
15	7.28	2186.6	404.4
16	7.26	2178.4	435.0
17	7.23	2170.1	222.0
18	7.20	2162.7	85.5
19	7.08	2125.5	1218.2
20	3.35	1004.9	90.1
21	12.94	3883.3	6.9

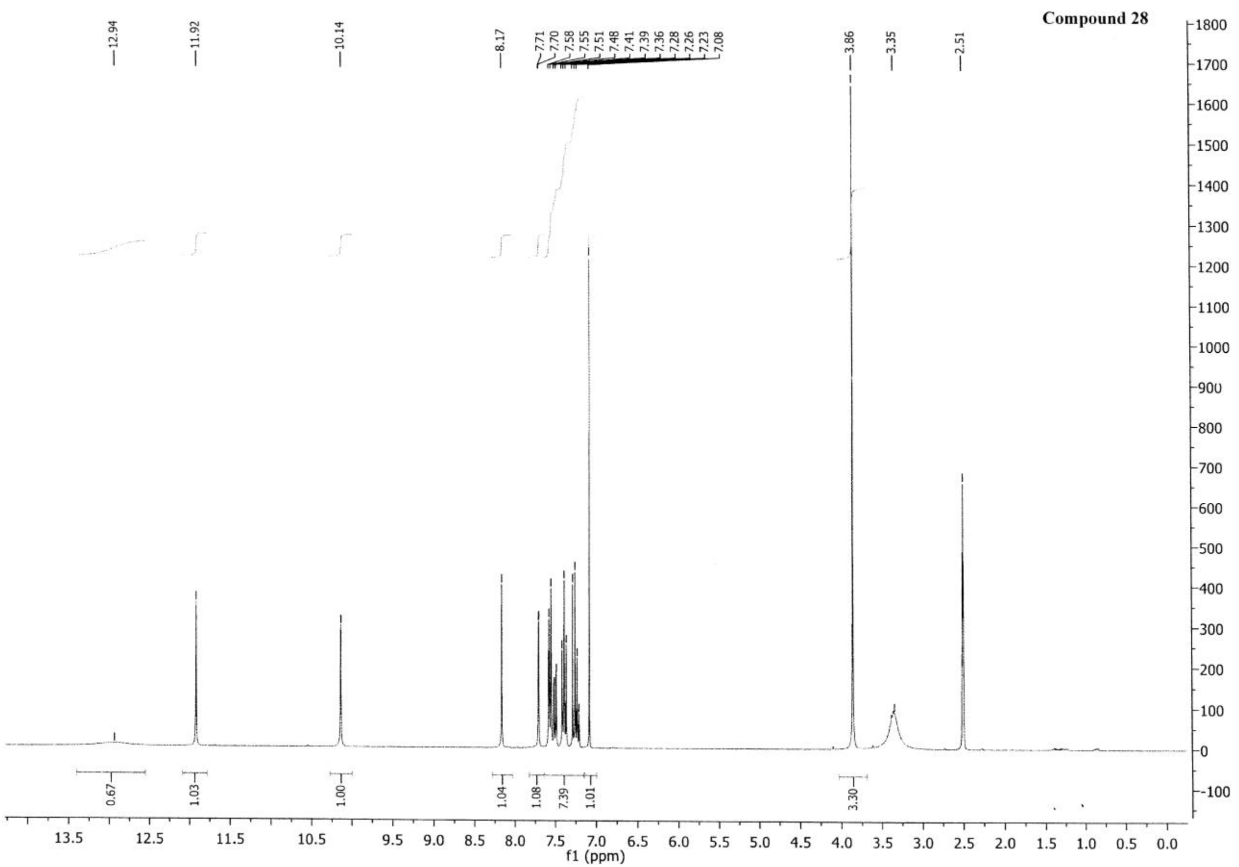

^{13}C NMR spectra for compounds (12-28)

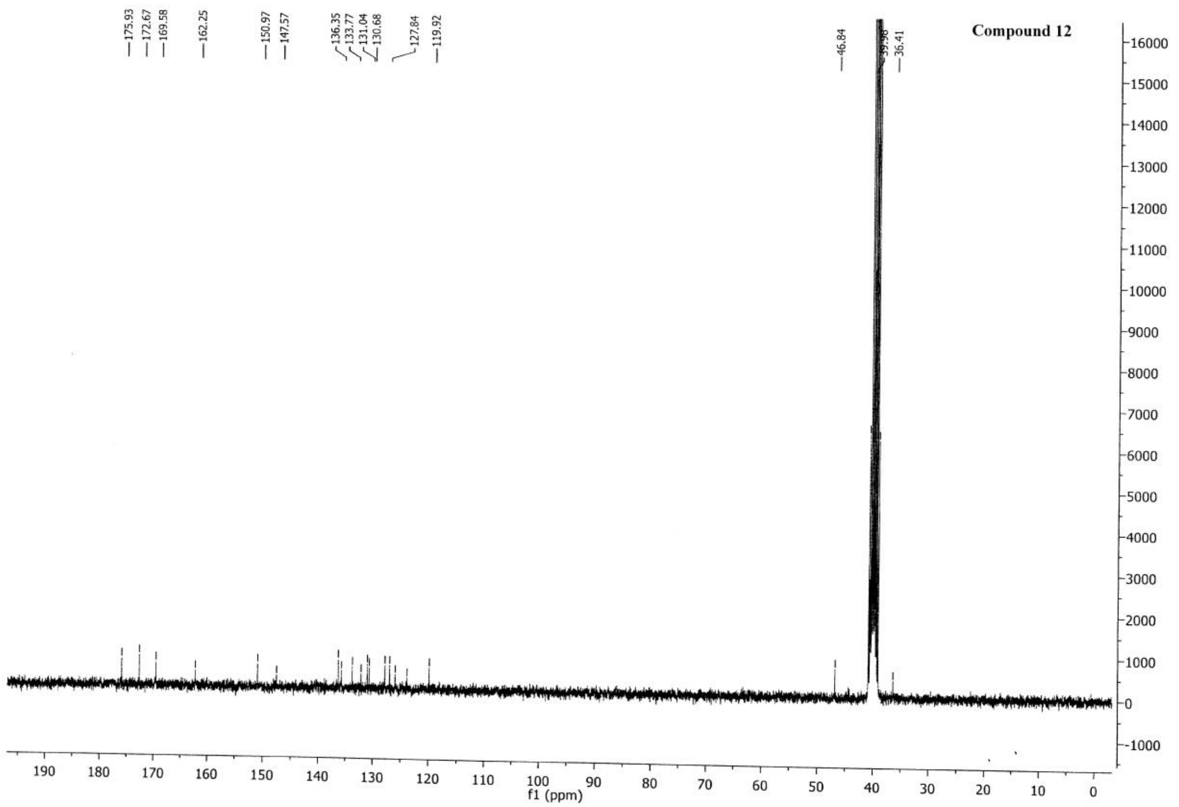

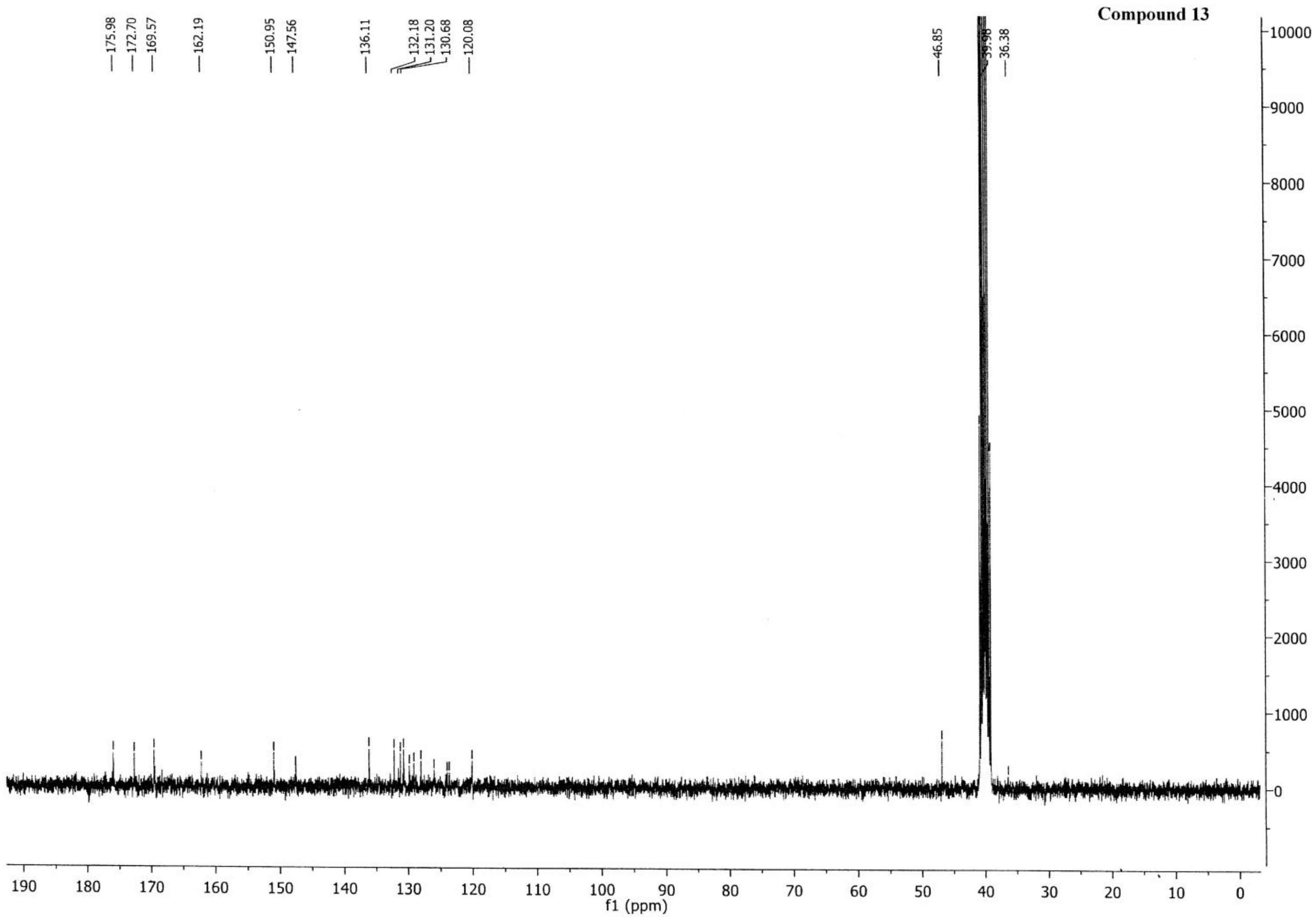

Compound 13
175.98
172.70
169.57
162.19
150.95
147.56
136.11
132.18
131.20
130.68
120.08
46.85
39.98
36.38
10000
9000
8000
7000
6000
5000
4000
3000
2000
1000
0
190
180
170
160
150
140
130
120
110
100
90
80
70
60
50
40
30
20
10
0
f1 (ppm)

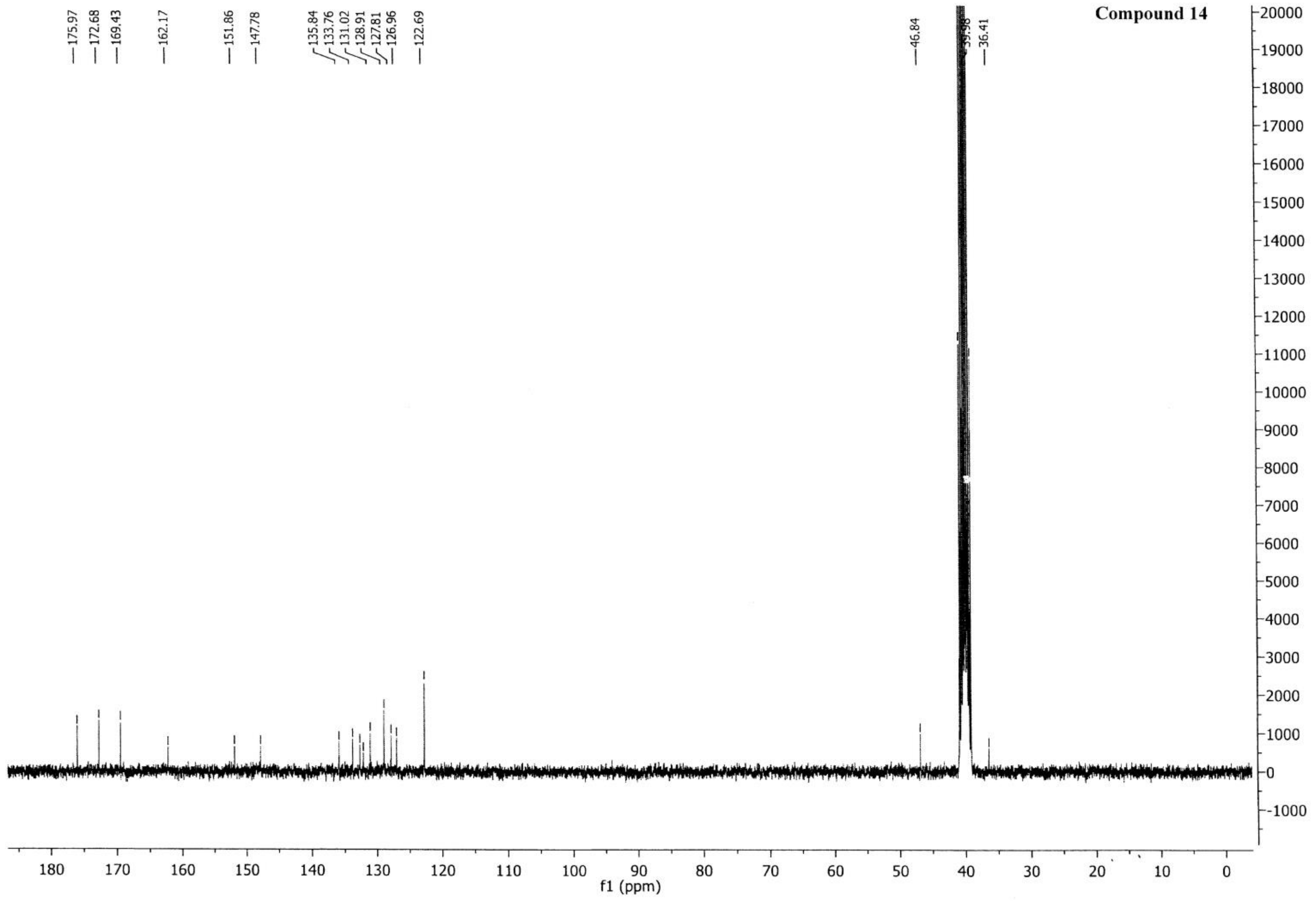
Compound 14
175.97
172.68
169.43
162.17
151.86
147.78
135.84
133.76
131.02
128.91
127.81
126.96
122.69
46.84
39.98
36.41
20000
19000
18000
17000
16000
15000
14000
13000
12000
11000
10000
9000
8000
7000
6000
5000
4000
3000
2000
1000
0
-1000
180
170
160
150
140
130
120
110
100
90
80
70
60
50
40
30
20
10
0
f1 (ppm)

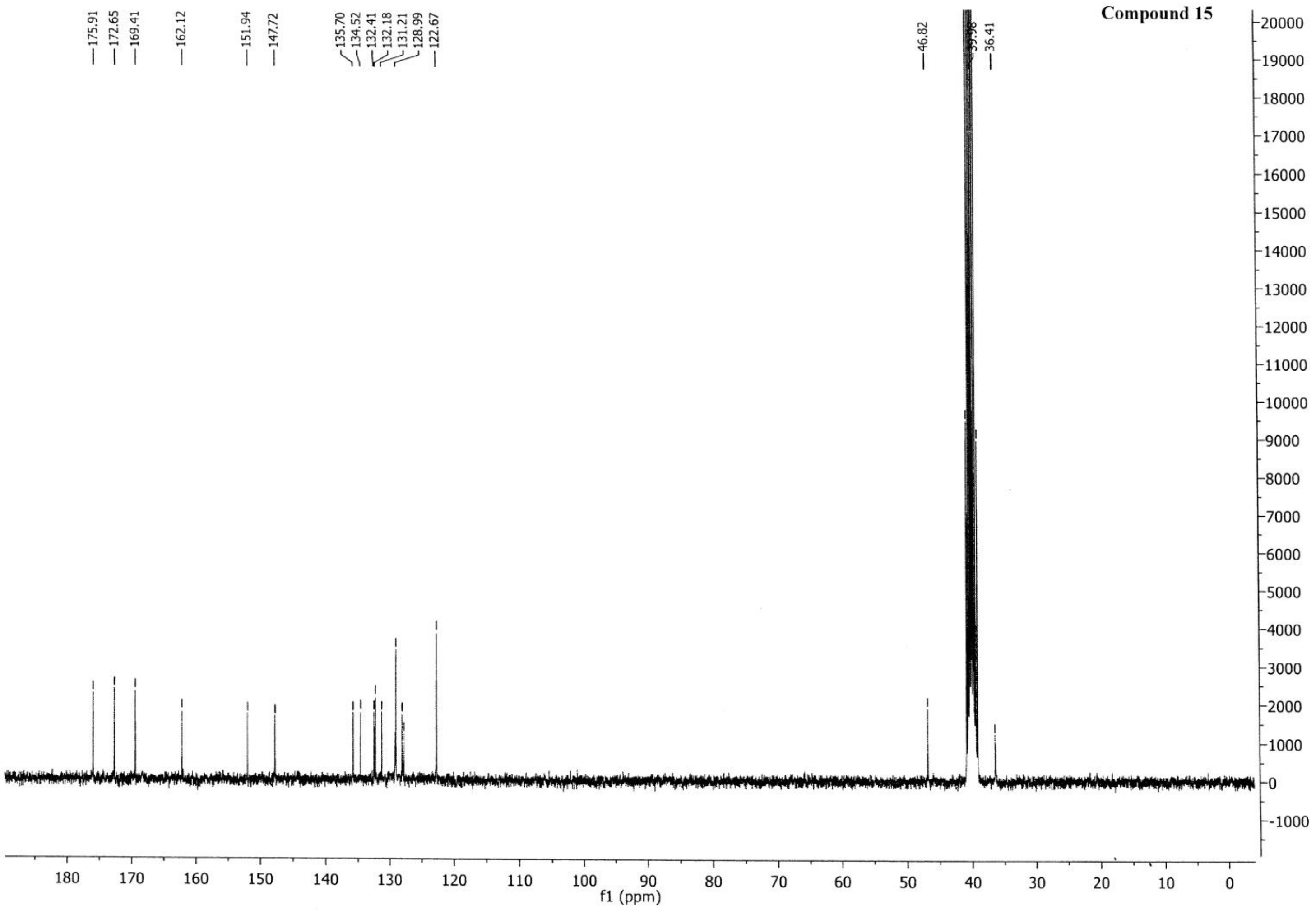
Compound 15
175.91
172.65
169.41
162.12
151.94
147.72
135.70
134.52
132.41
132.18
131.21
128.99
122.67
46.82
36.41
f1 (ppm)

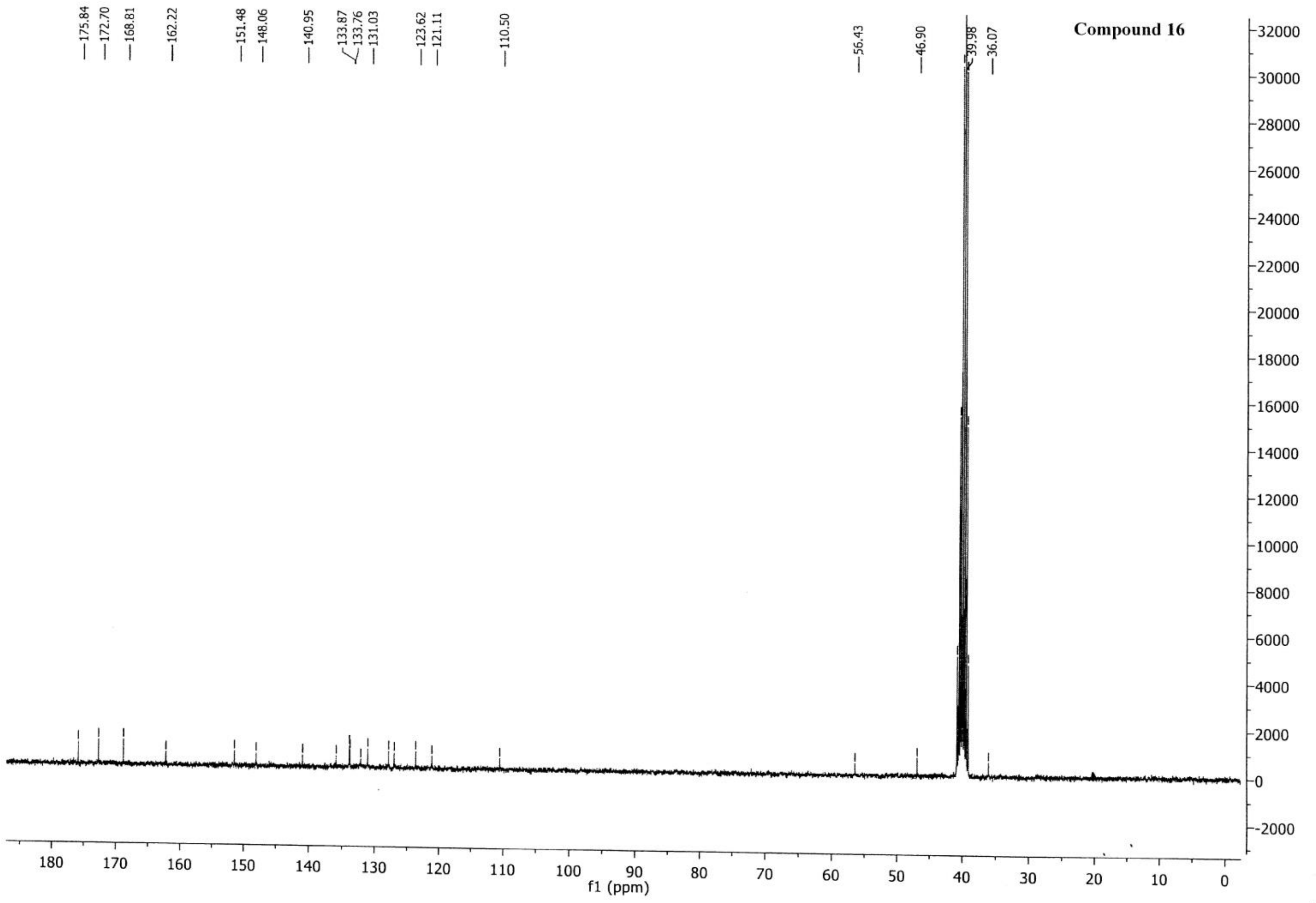

175.84
172.70
168.81
162.22
151.48
148.06
140.95
133.87
133.76
131.03
123.62
121.11
110.50
56.43
46.90
39.98
36.07
Compound 16
f1 (ppm)

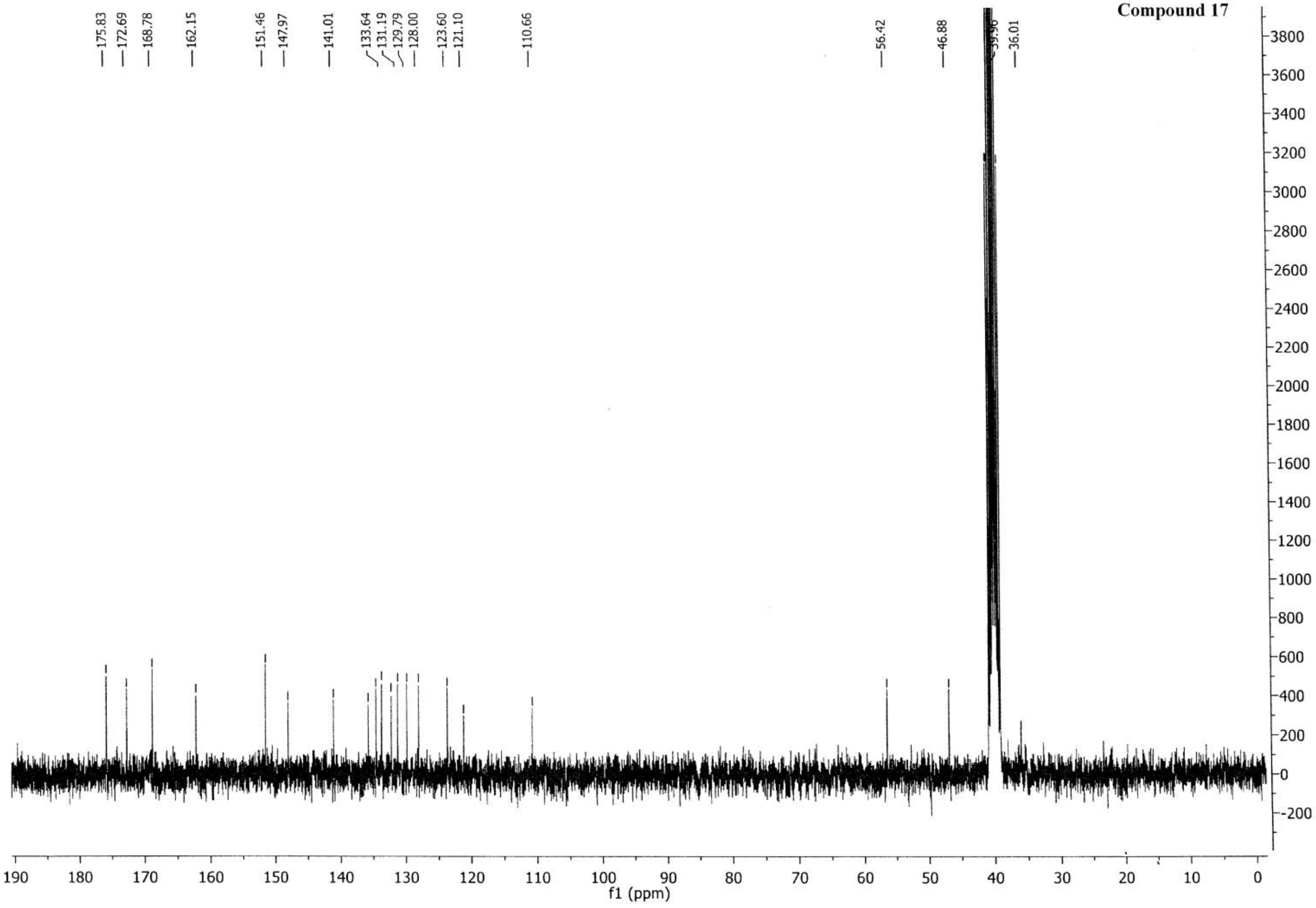
Compound 17
175.83
172.69
168.78
162.15
151.46
147.97
141.01
133.64
131.19
129.79
128.00
123.60
121.10
110.66
56.42
46.88
36.01
f1 (ppm)

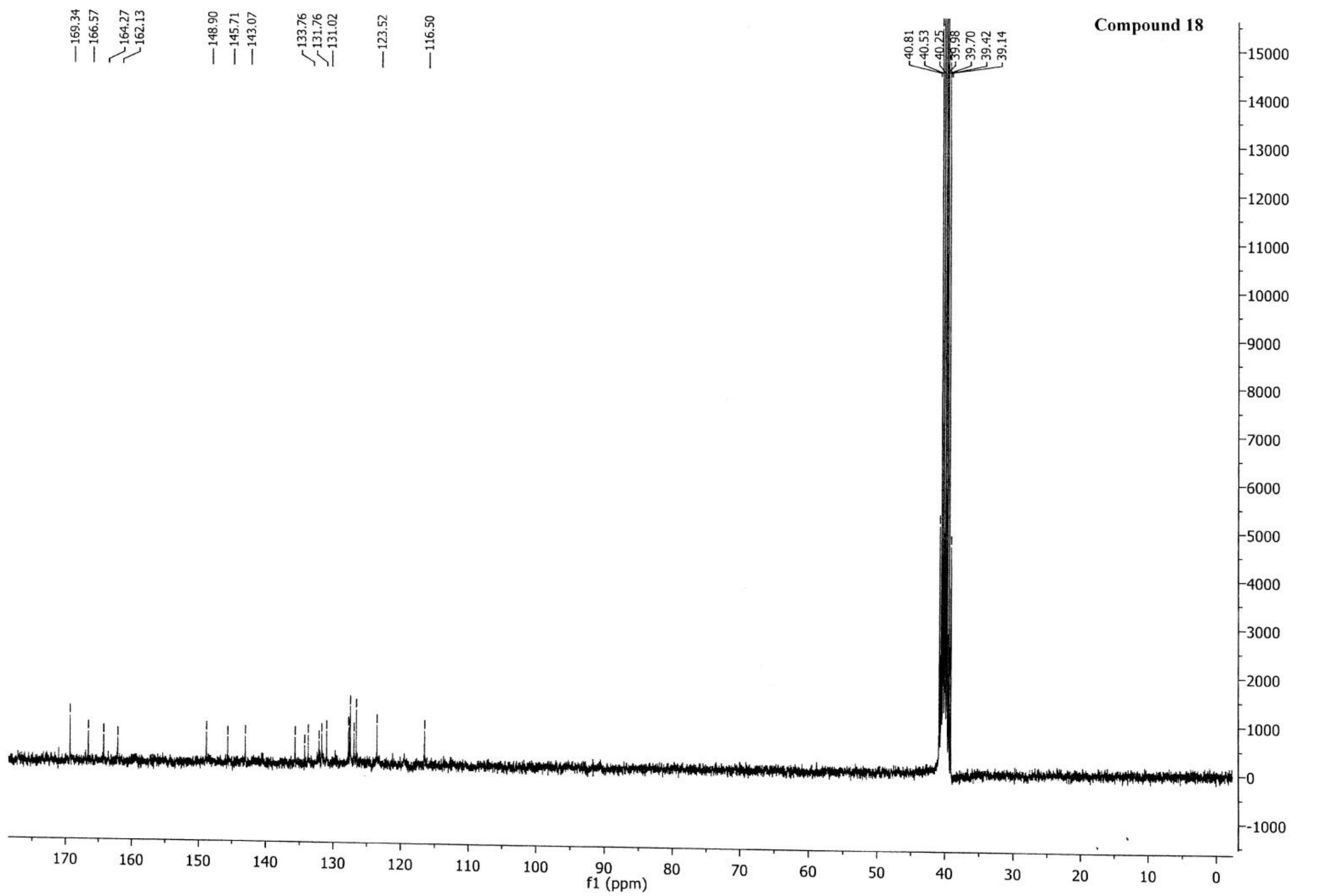

Compound 18
169.34
166.57
164.27
162.13
148.90
145.71
143.07
133.76
131.76
131.02
123.52
116.50
40.81
40.53
40.25
39.98
39.70
39.42
39.14
f1 (ppm)

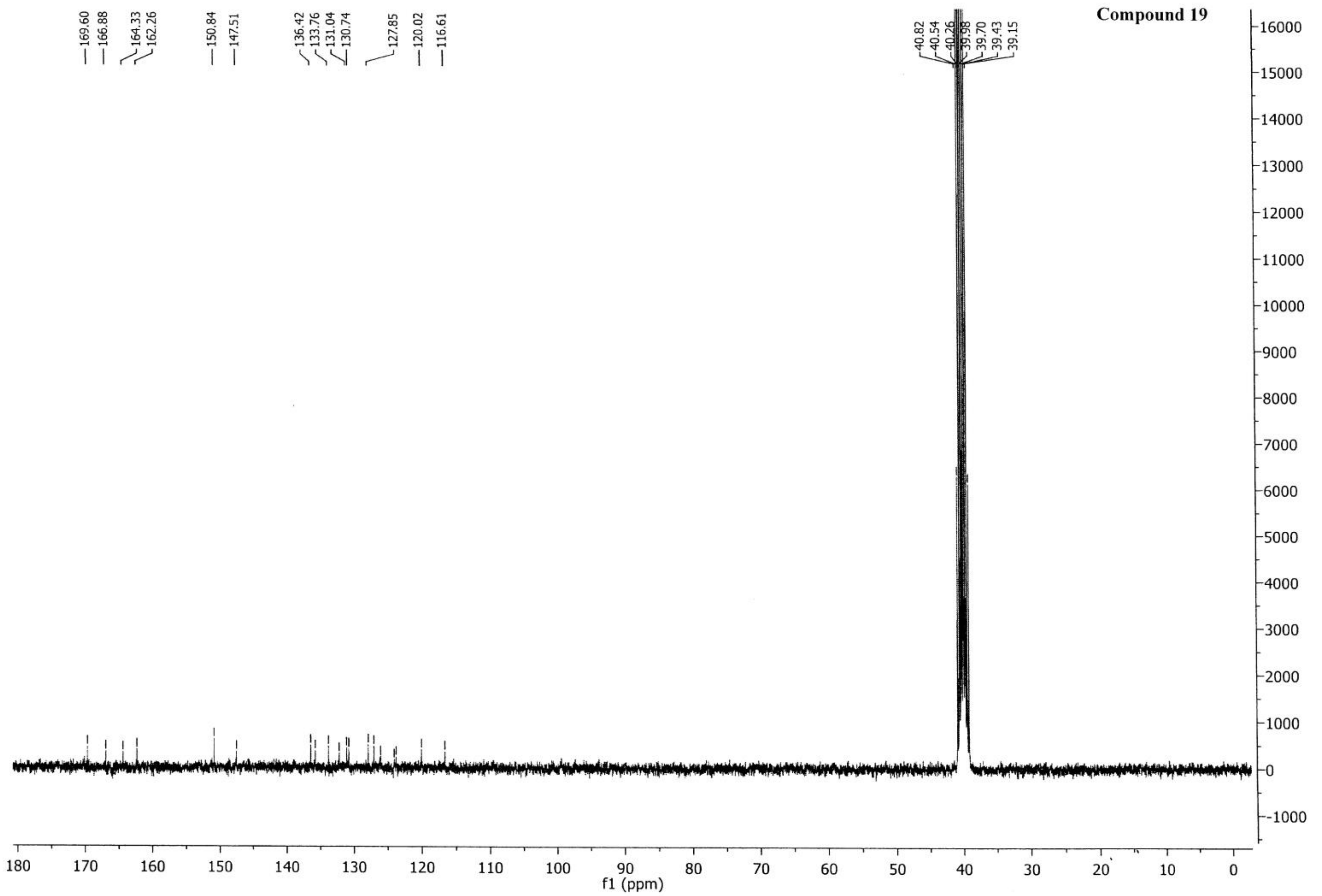
Compound 19
169.60
166.88
164.33
162.26
150.84
147.51
136.42
133.76
131.04
130.74
127.85
120.02
116.61
40.82
40.54
40.26
39.98
39.70
39.43
39.15
f1 (ppm)

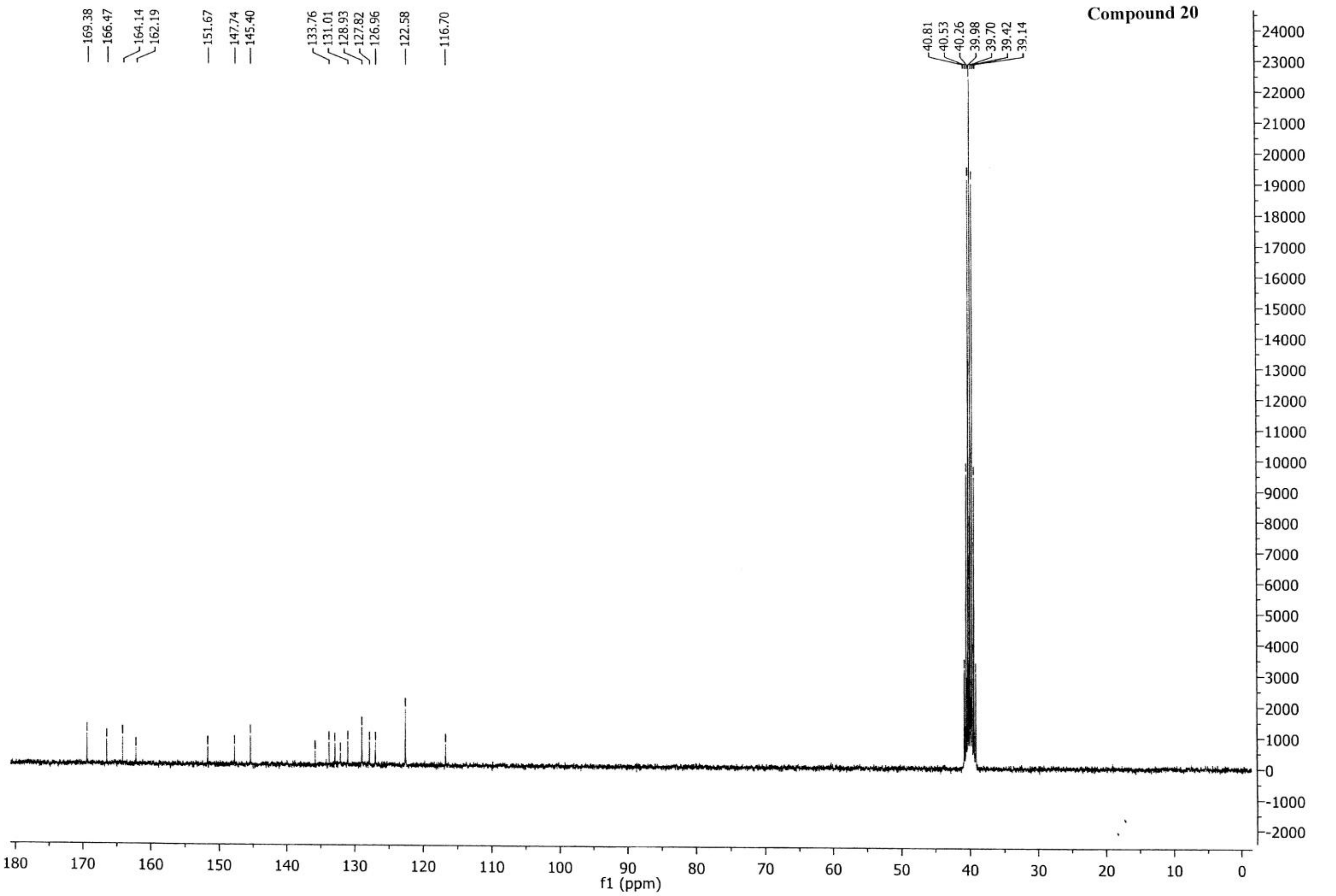

Compound 20
169.38
166.47
164.14
162.19
151.67
147.74
145.40
133.76
131.01
128.93
127.82
126.96
122.58
116.70
40.81
40.53
40.26
39.98
39.70
39.42
39.14
f1 (ppm)

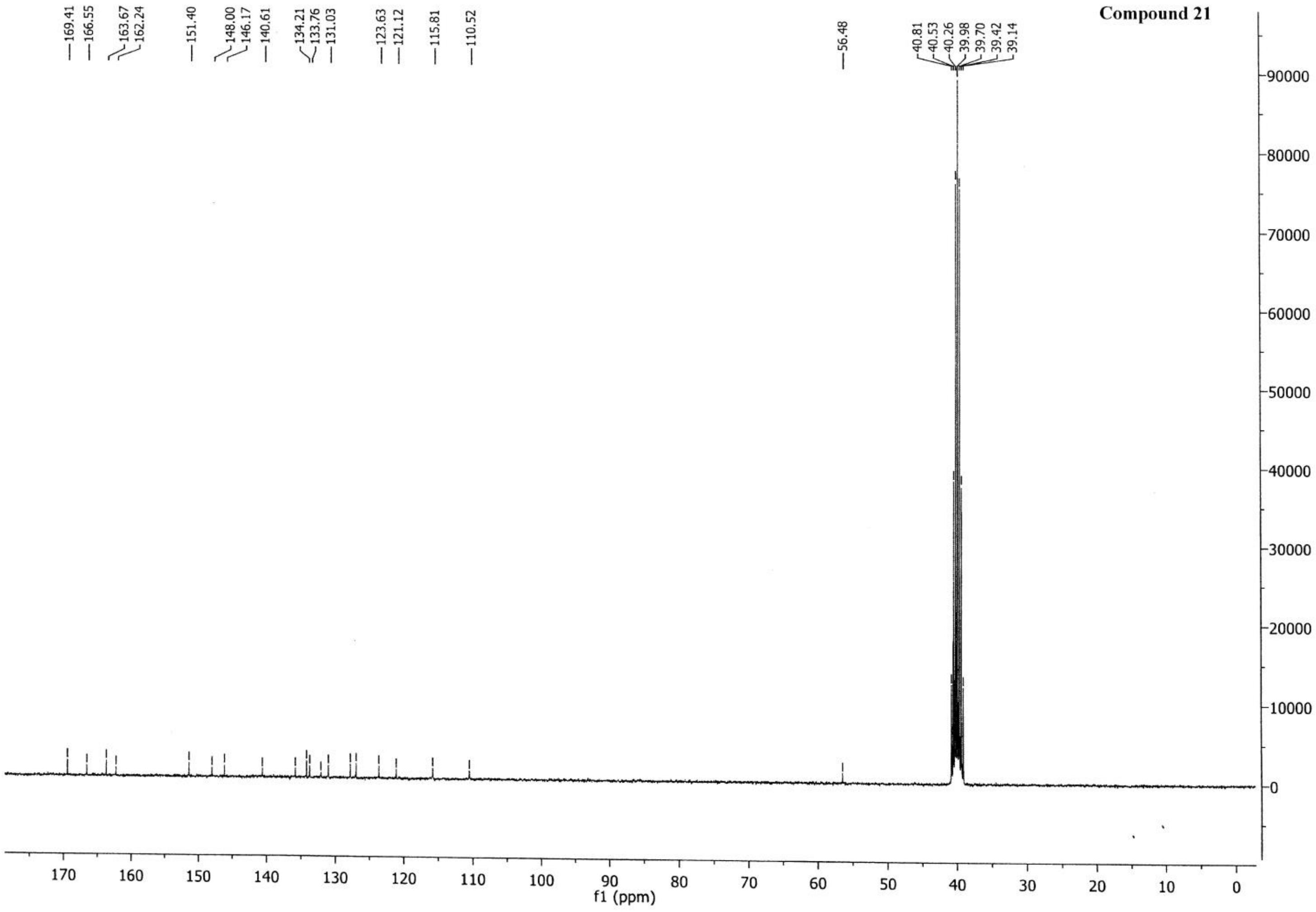
Compound 21
169.41
166.55
163.67
162.24
151.40
148.00
146.17
140.61
134.21
133.76
131.03
123.63
121.12
115.81
110.52
56.48
40.81
40.53
40.26
39.98
39.70
39.42
39.14
f1 (ppm)

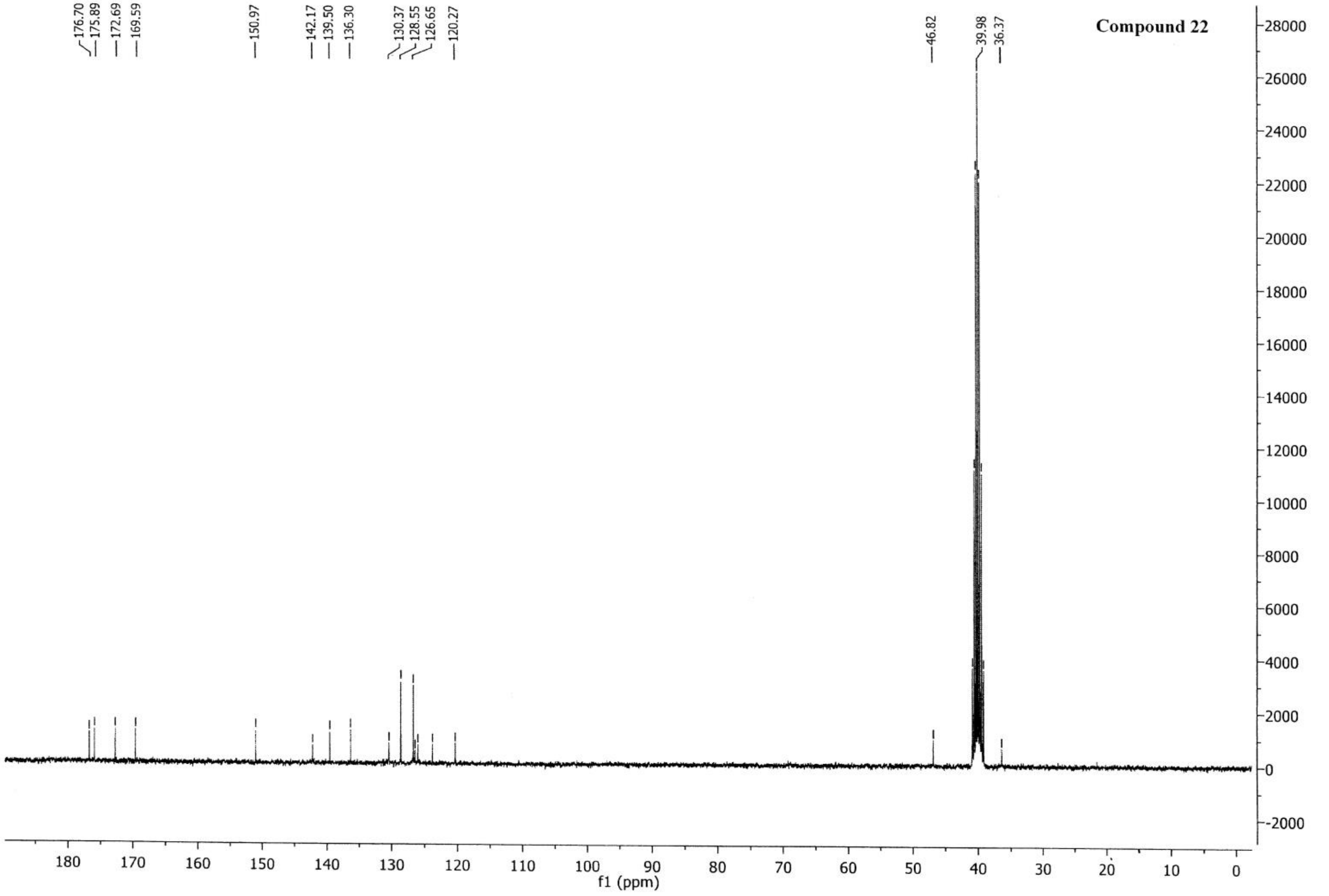

176.70
175.89
172.69
169.59
150.97
142.17
139.50
136.30
130.37
128.55
126.65
120.27
46.82
39.98
36.37
Compound 22
28000
26000
24000
22000
20000
18000
16000
14000
12000
10000
8000
6000
4000
2000
0
-2000
180 170 160 150 140 130 120 110 100 90 80 70 60 50 40 30 20 10 0
f1 (ppm)

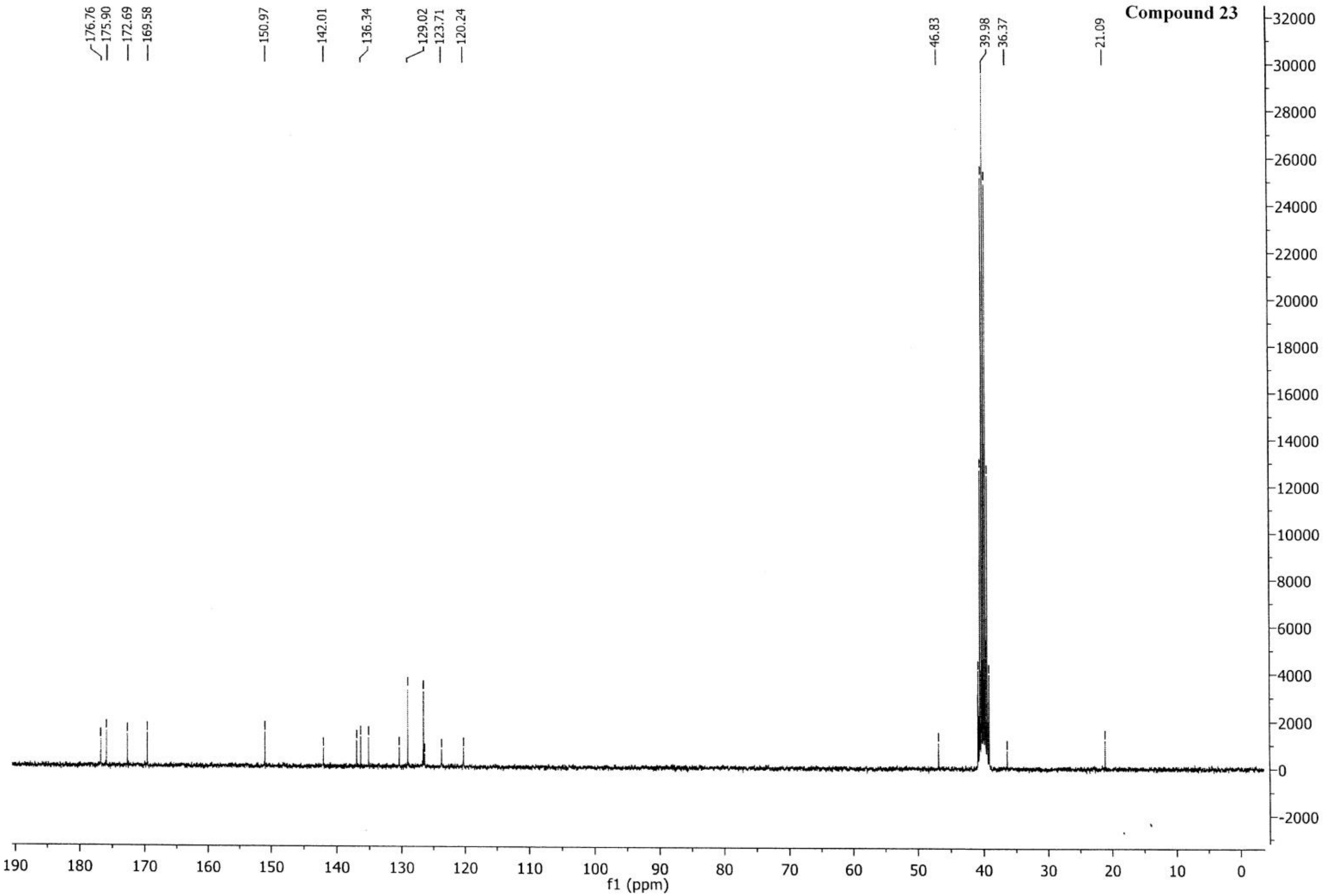

Compound 23
176.76
175.90
172.69
169.58
150.97
142.01
136.34
129.02
123.71
120.24
46.83
39.98
36.37
21.09
f1 (ppm)

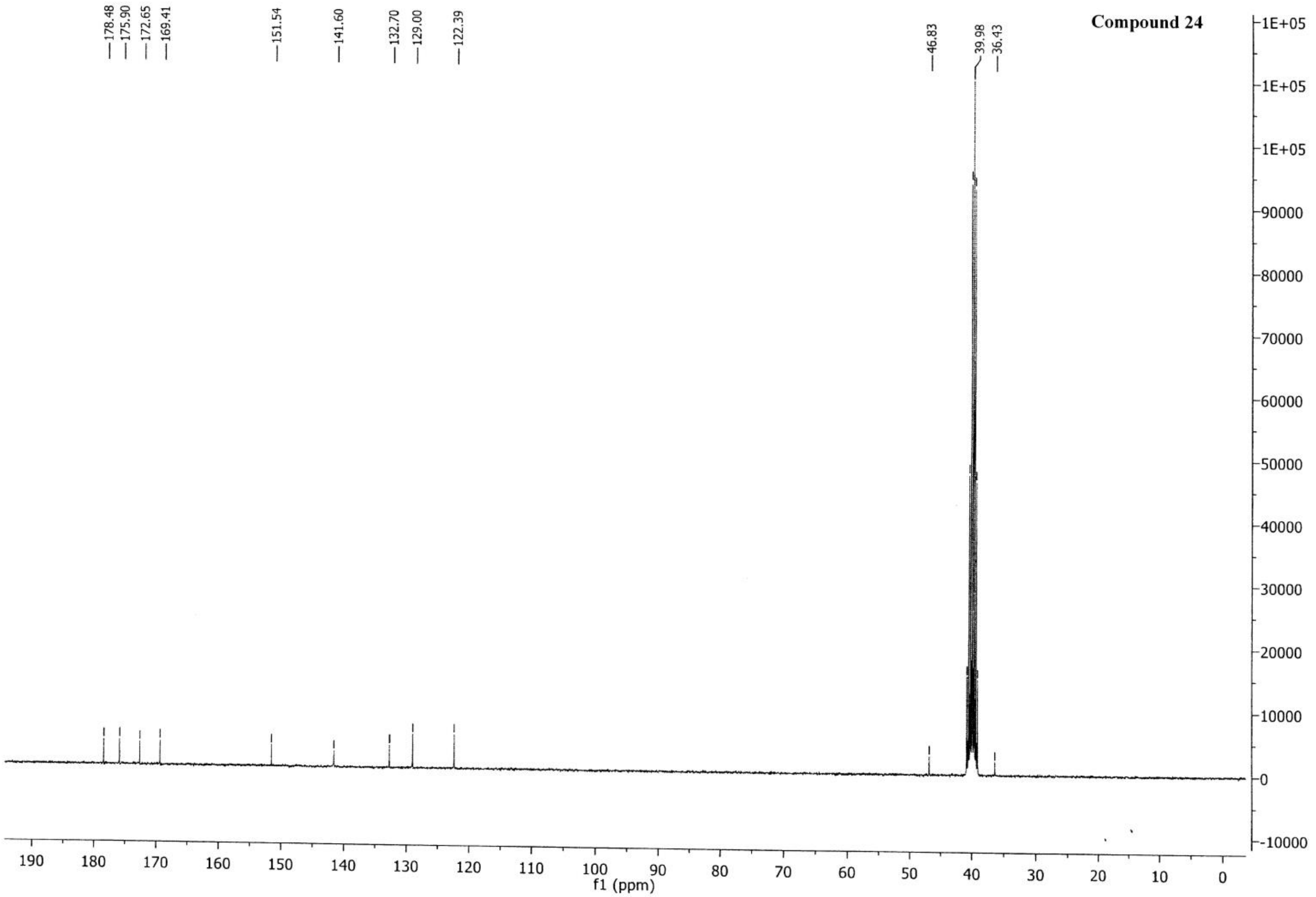

178.48
175.90
172.65
169.41
151.54
141.60
132.70
129.00
122.39
46.83
39.98
36.43
Compound 24
1E+05
1E+05
1E+05
90000
80000
70000
60000
50000
40000
30000
20000
10000
0
-10000
190
180
170
160
150
140
130
120
110
100
90
80
70
60
50
40
30
20
10
0
f1 (ppm)

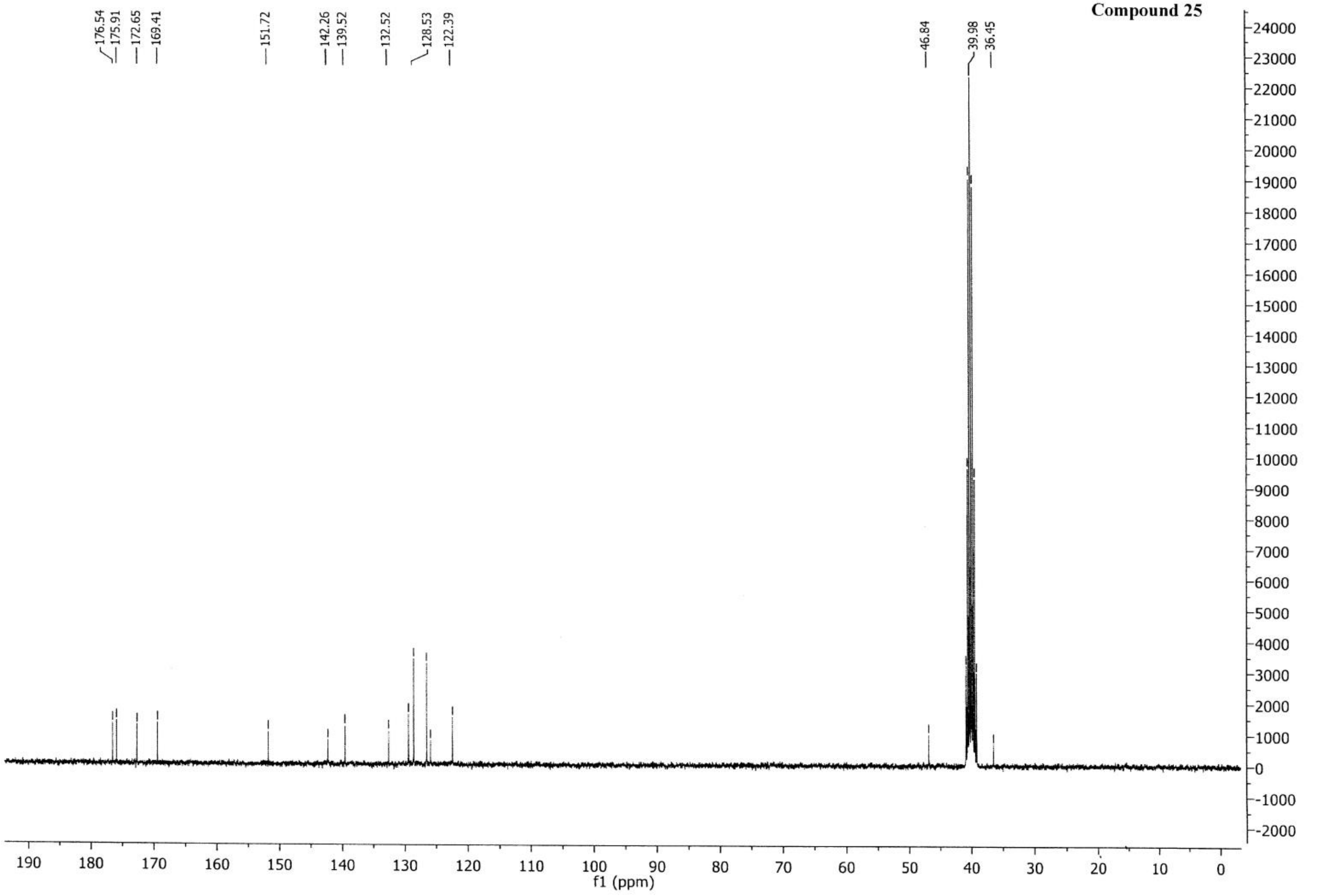

Compound 25
176.54
175.91
172.65
169.41
151.72
142.26
139.52
132.52
128.53
122.39
46.84
39.98
36.45
f1 (ppm)

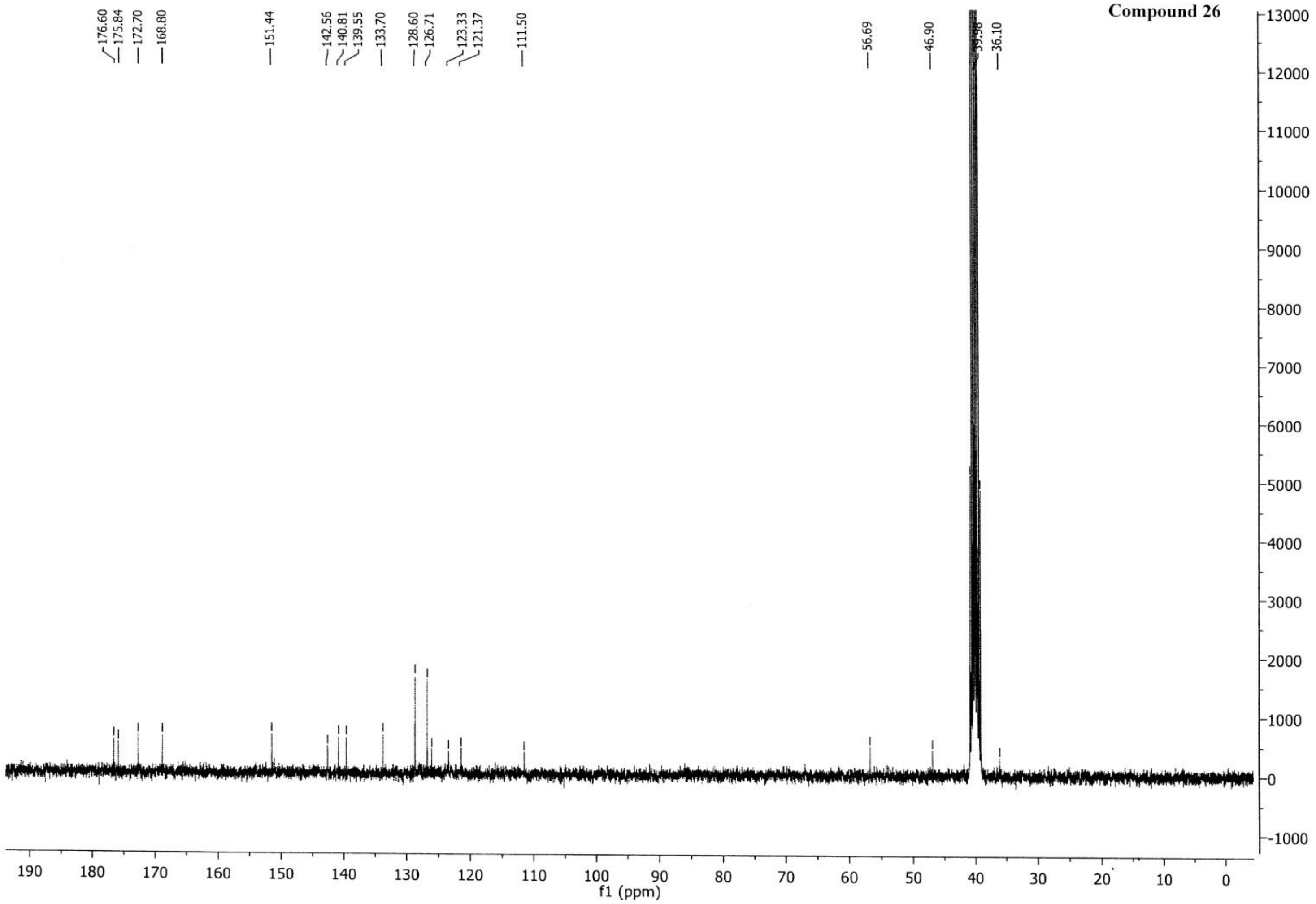

Compound 26
176.60
175.84
172.70
168.80
151.44
142.56
140.81
139.55
133.70
128.60
126.71
123.33
121.37
111.50
56.69
46.90
36.10
13000
12000
11000
10000
9000
8000
7000
6000
5000
4000
3000
2000
1000
0
-1000
190
180
170
160
150
140
130
120
110
100
90
80
70
60
50
40
30
20
10
0
f1 (ppm)

Compound 27

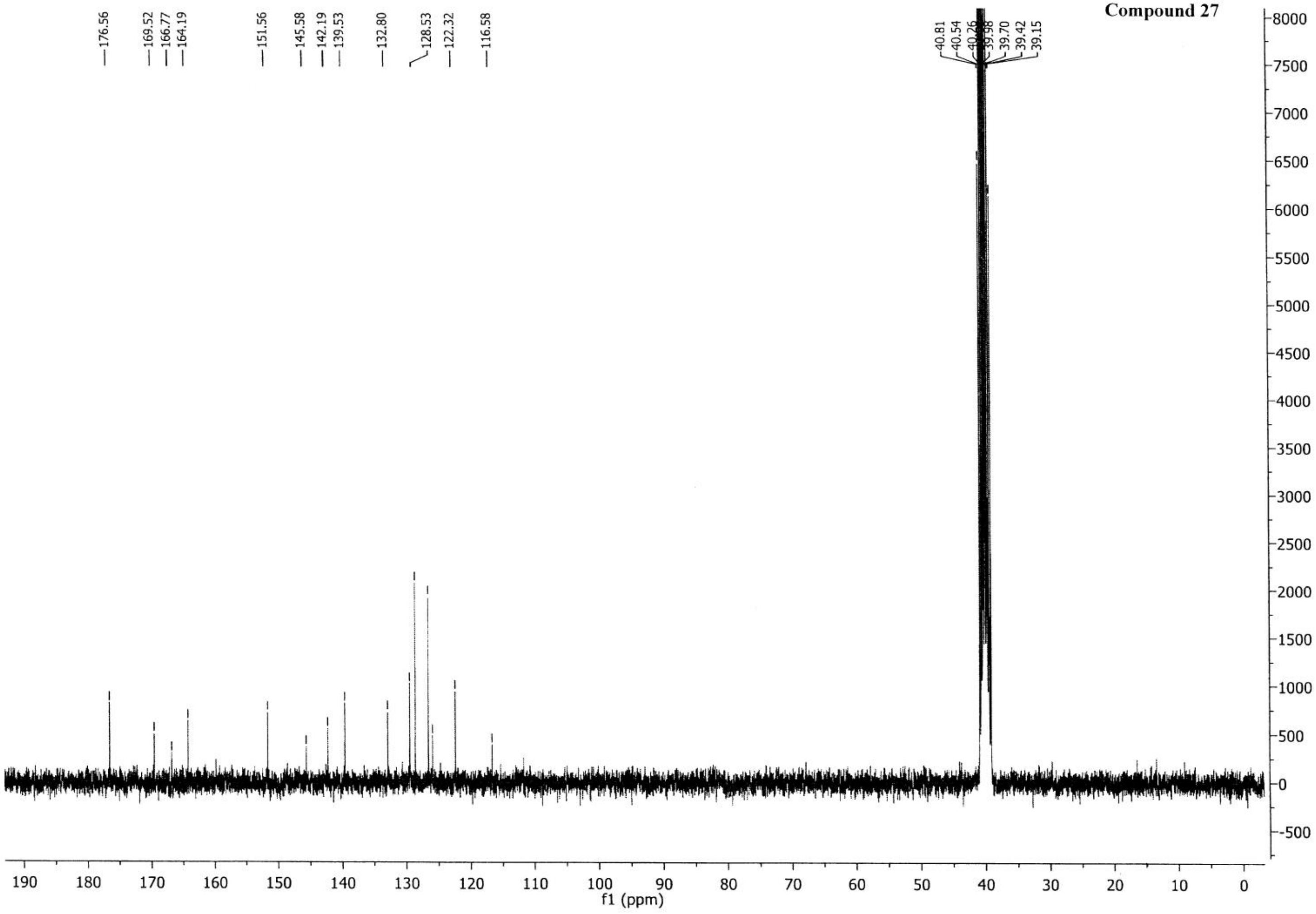

176.56
169.52
166.77
164.19
151.56
145.58
142.19
139.53
132.80
128.53
122.32
116.58
40.81
40.54
40.26
39.98
39.70
39.42
39.15
f1 (ppm)

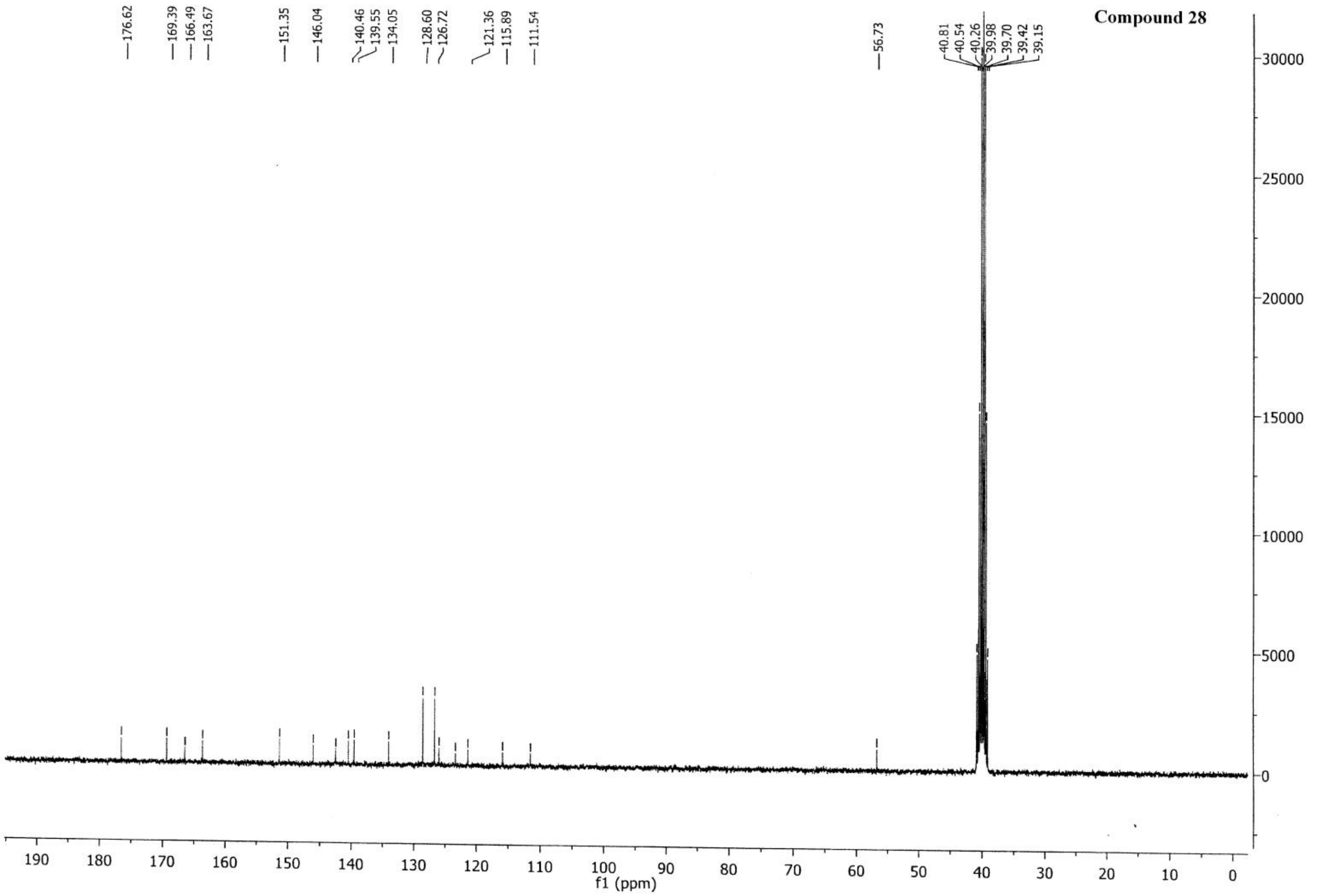

Compound 28
176.62
169.39
166.49
163.67
151.35
146.04
140.46
139.55
134.05
128.60
126.72
121.36
115.89
111.54
56.73
40.81
40.54
40.26
39.98
39.70
39.42
39.15
30000
25000
20000
15000
10000
5000
0
190
180
170
160
150
140
130
120
110
100
90
80
70
60
50
40
30
20
10
0
f1 (ppm)

MS spectra for compounds (12-28)

Window Display Report

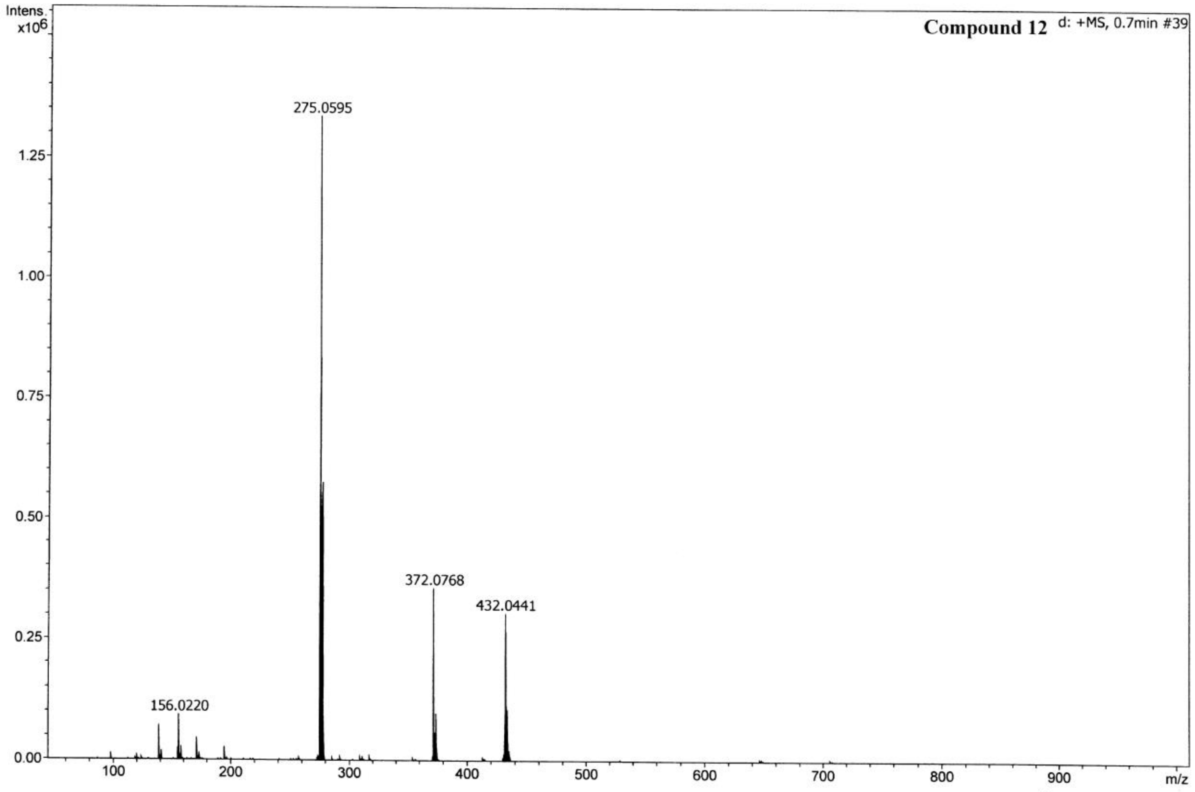

Bruker Compass DataAnalysis 4.1 printed: 4/6/2016 12:20:16 PM by: user Page 1 of 1

Window Display Report

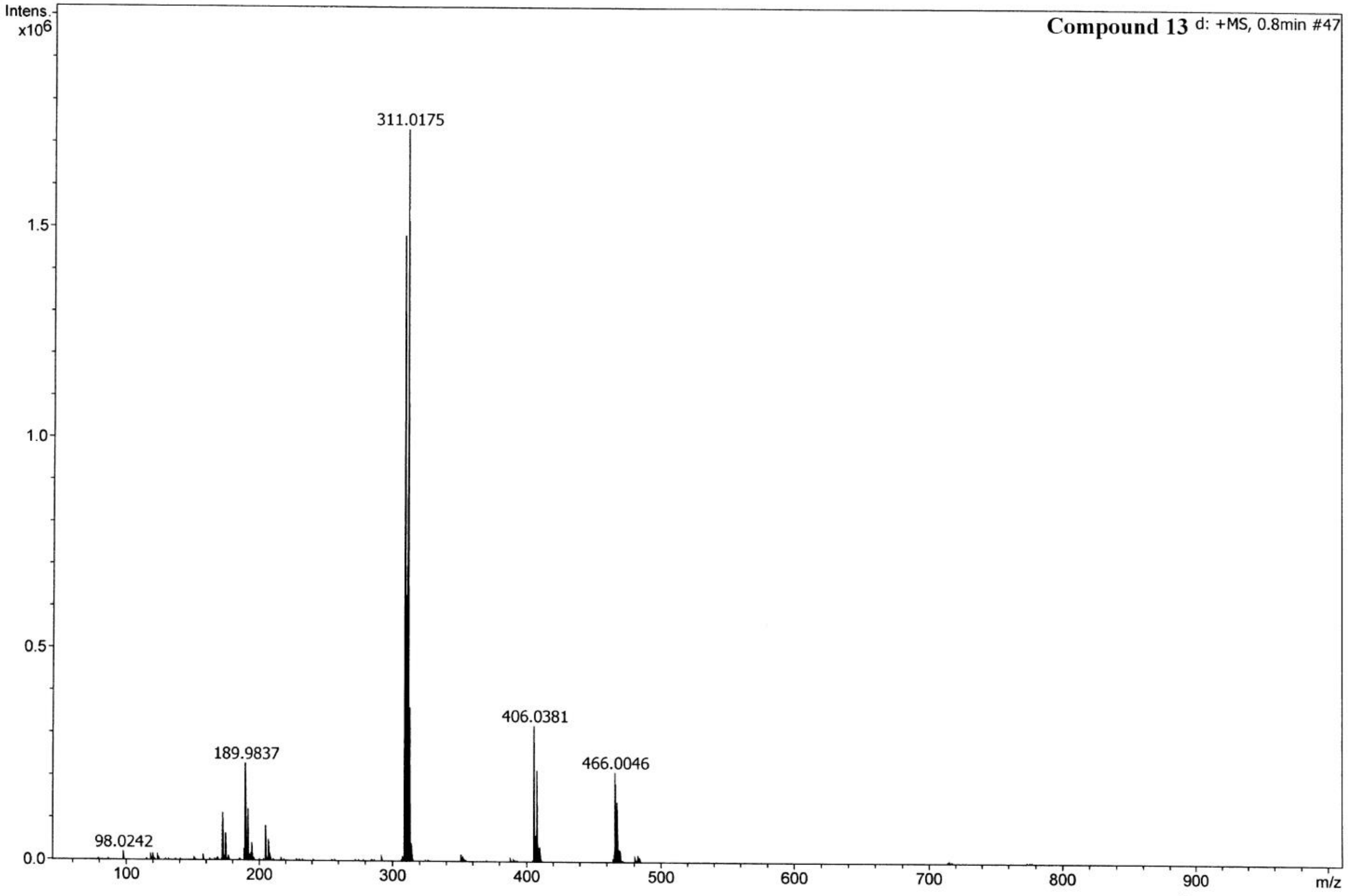

Window Display Report

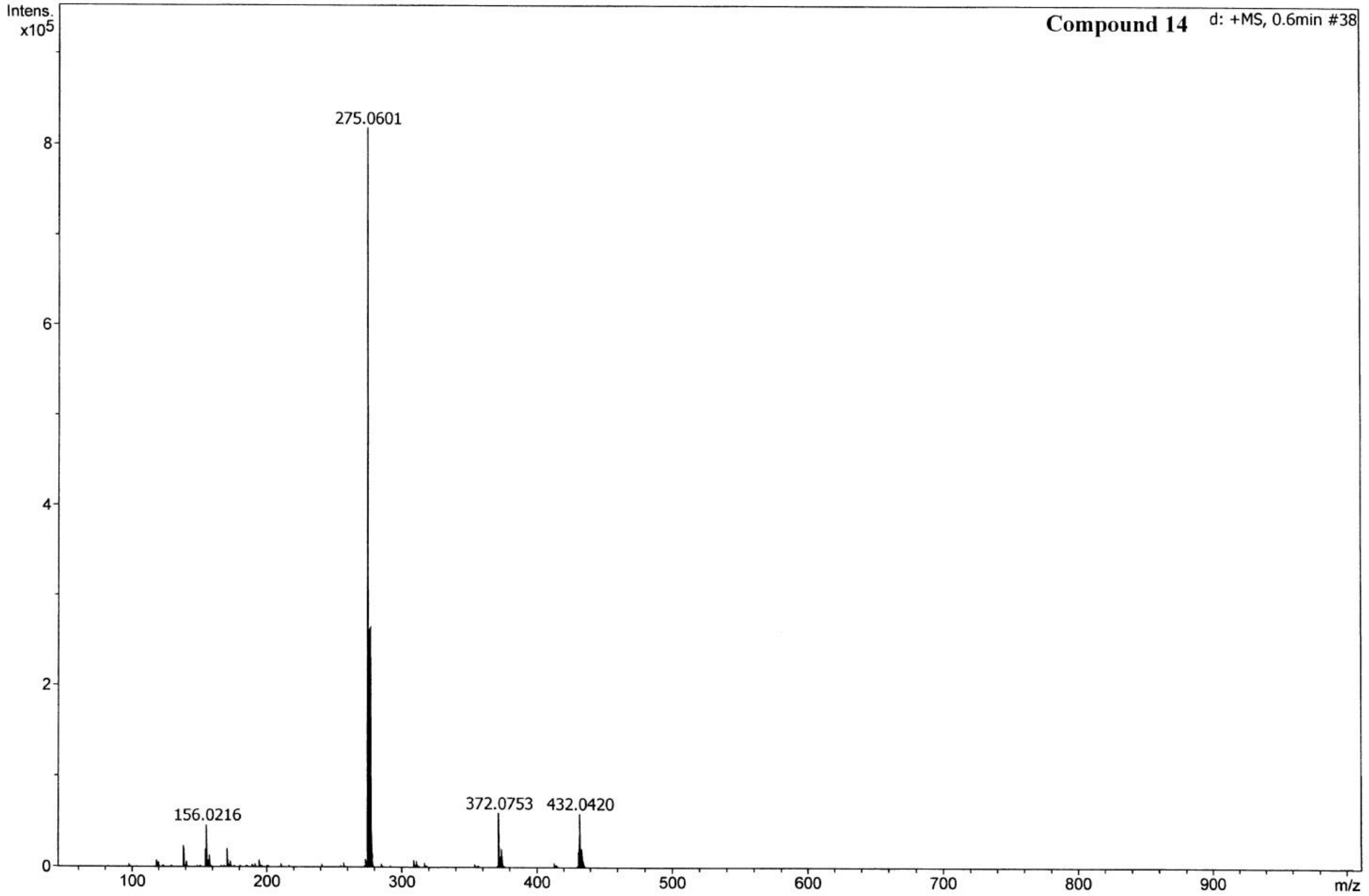

Window Display Report

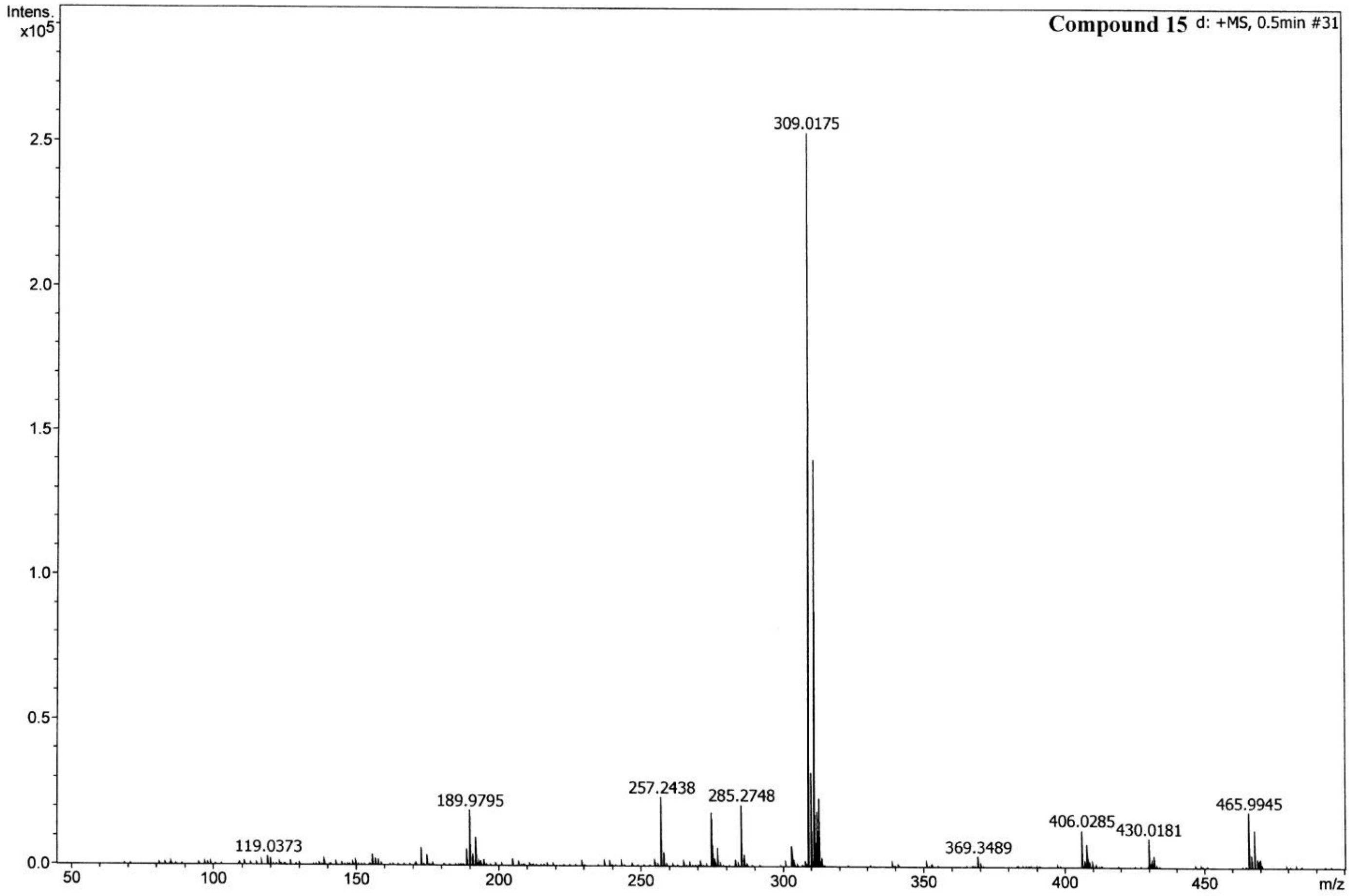

Window Display Report

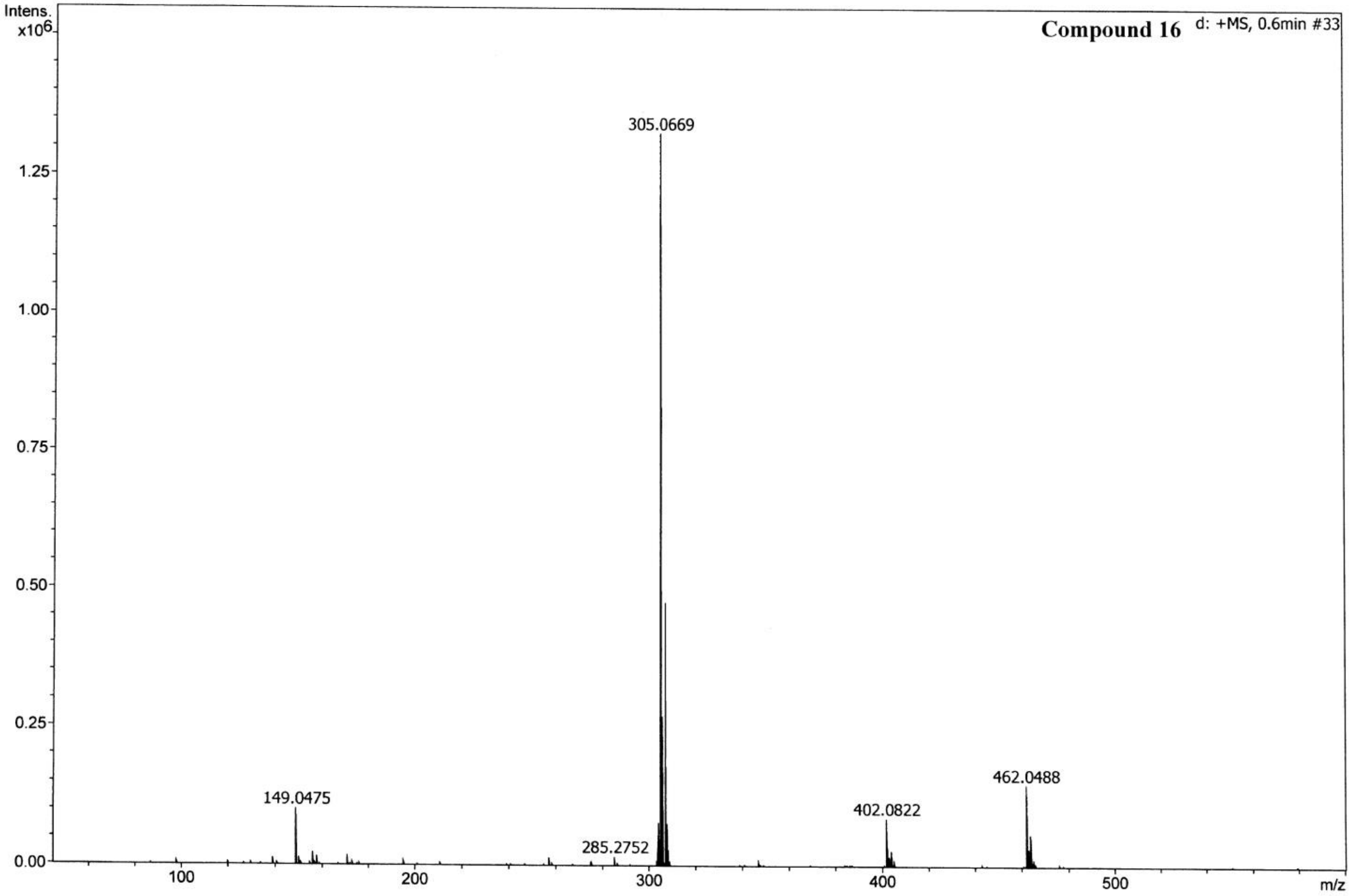

Window Display Report

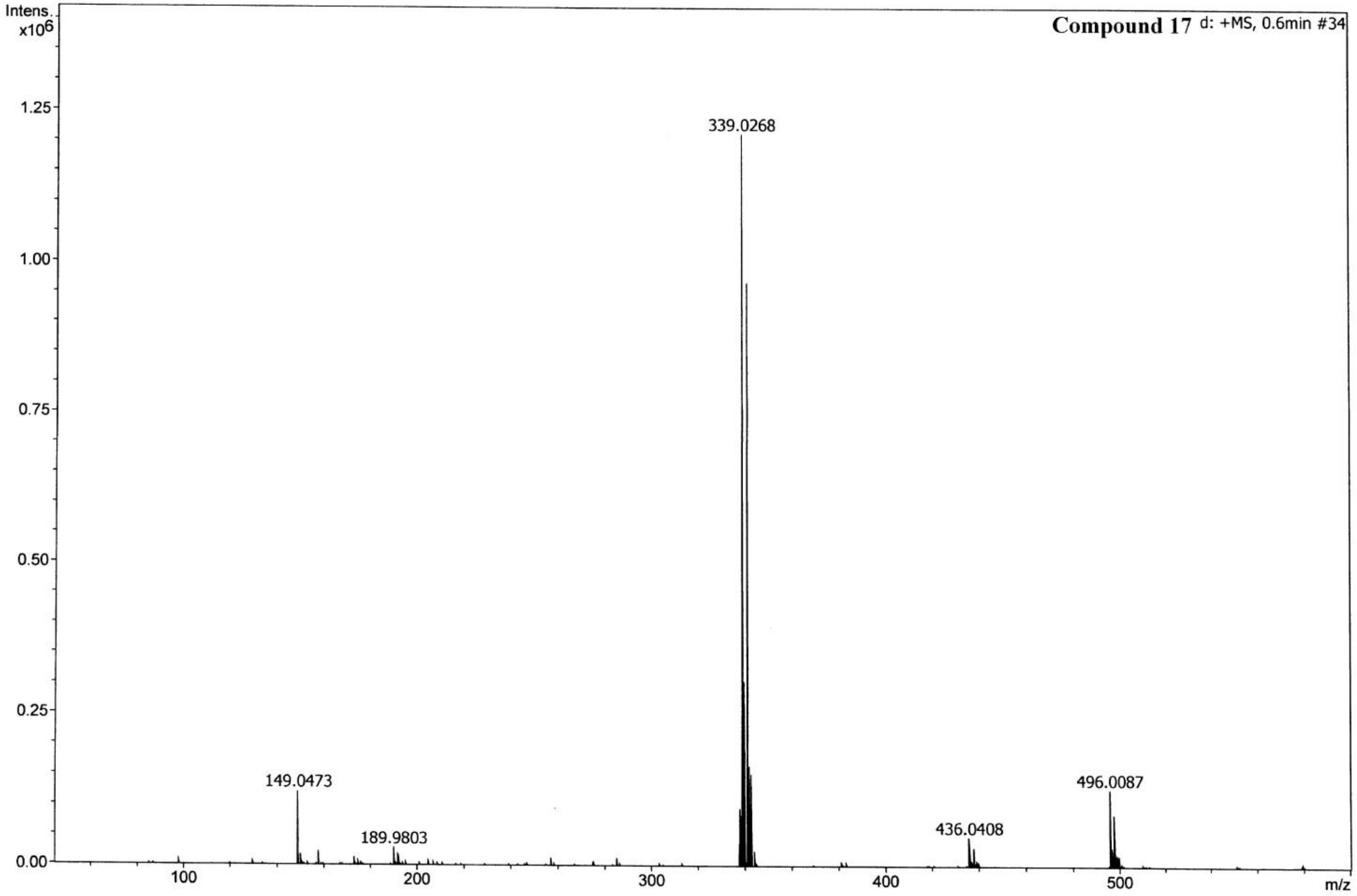

Window Display Report

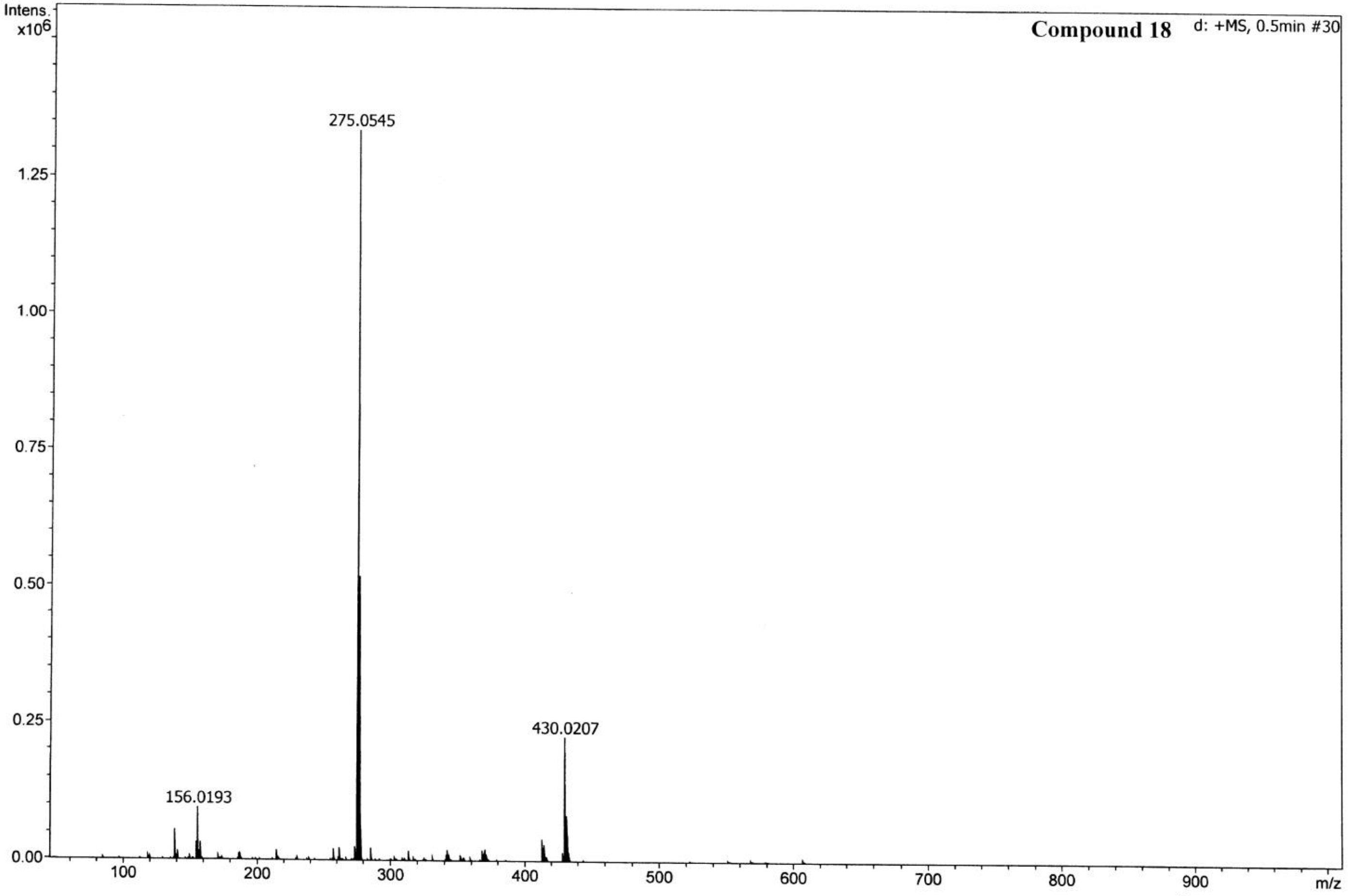

Window Display Report

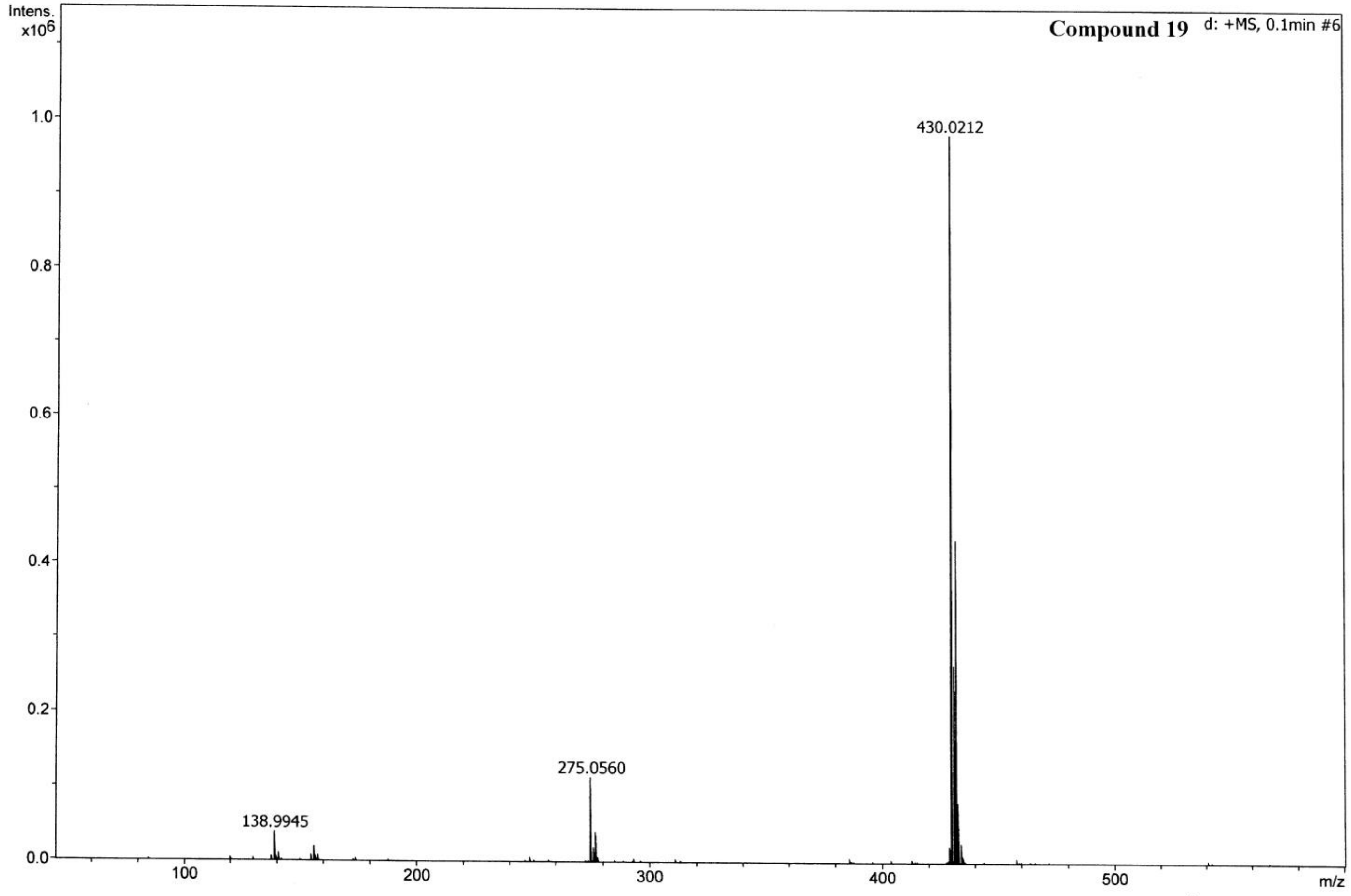

Window Display Report

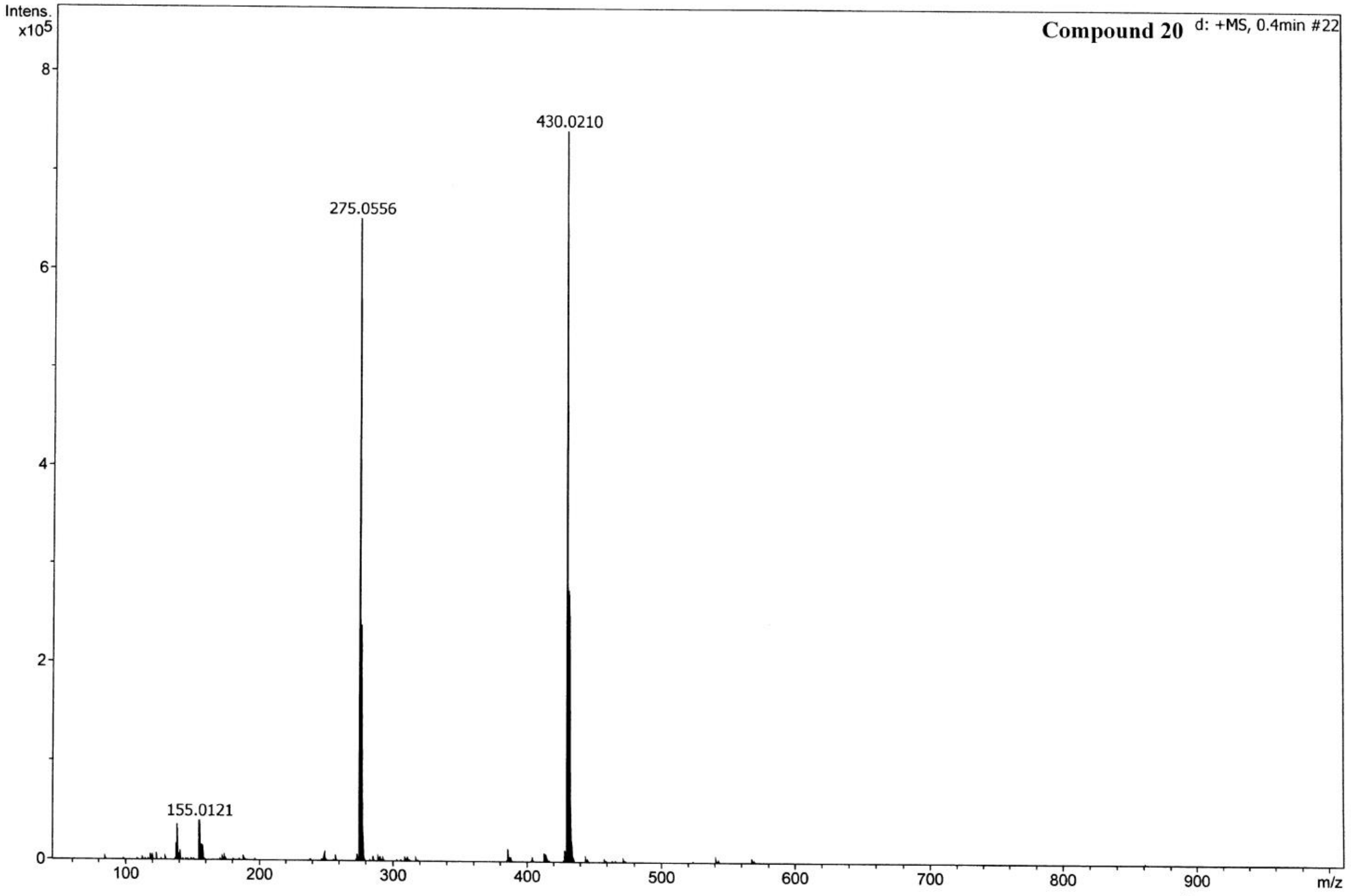

Window Display Report

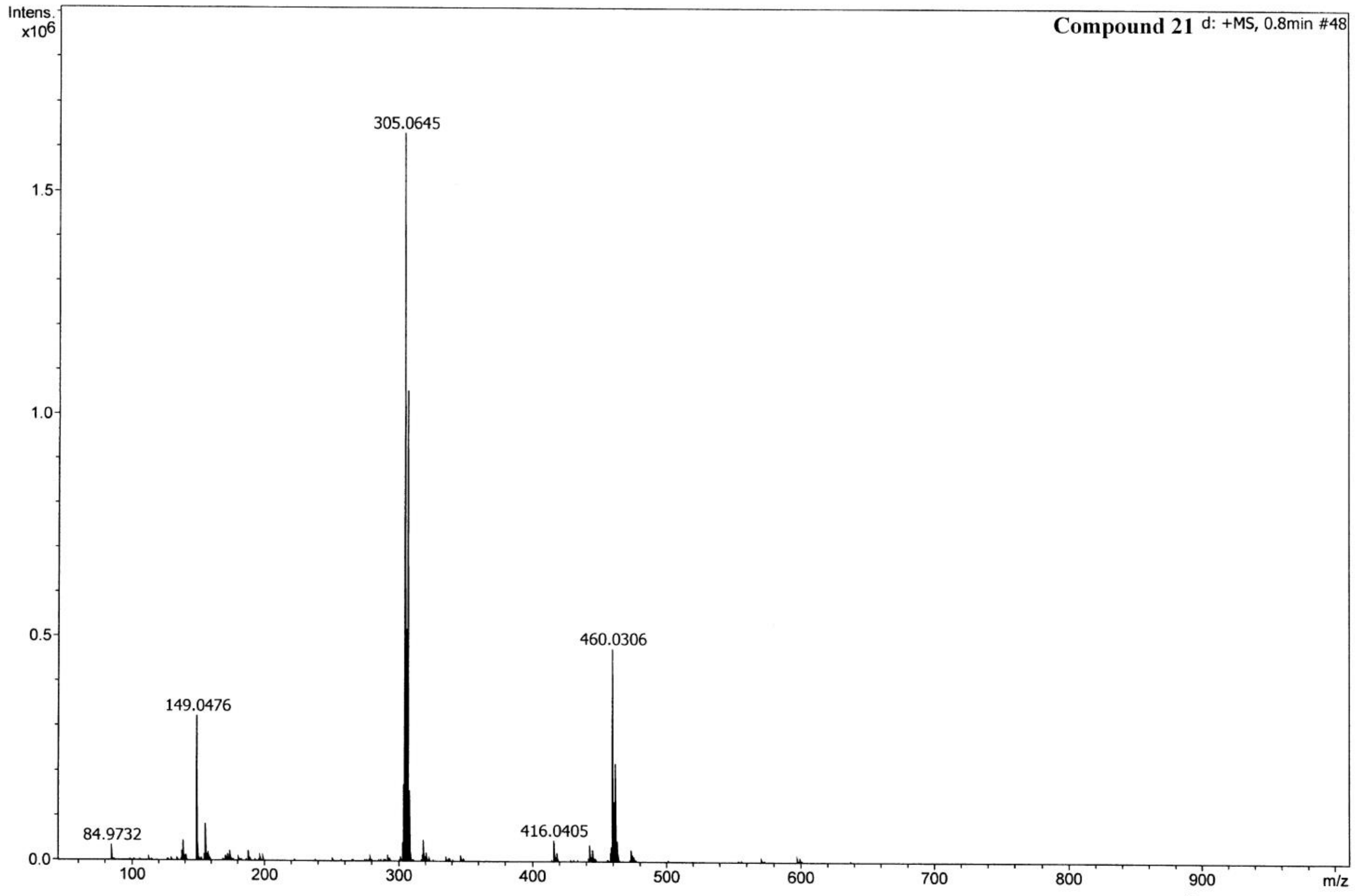

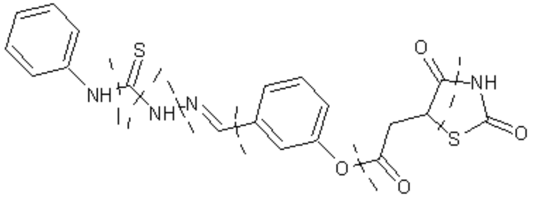
S
NH
NH
N
O
O
NH
S
O
O

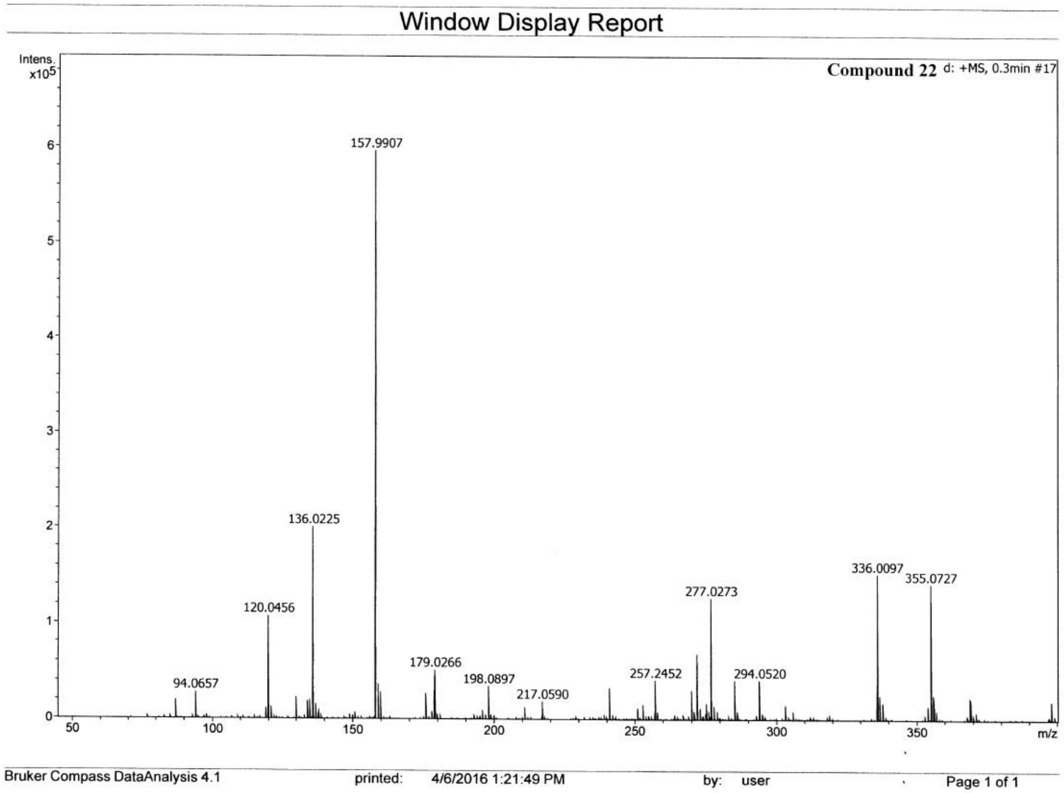
Window Display Report
Intens.
x10^5
Compound 22 d: +MS, 0.3min #17
157.9907
136.0225
120.0456
94.0657
179.0266
198.0897
217.0590
257.2452
277.0273
294.0520
336.0097
355.0727
50
100
150
200
250
300
350
m/z
Bruker Compass DataAnalysis 4.1
printed: 4/6/2016 1:21:49 PM
by: user
Page 1 of 1

Window Display Report

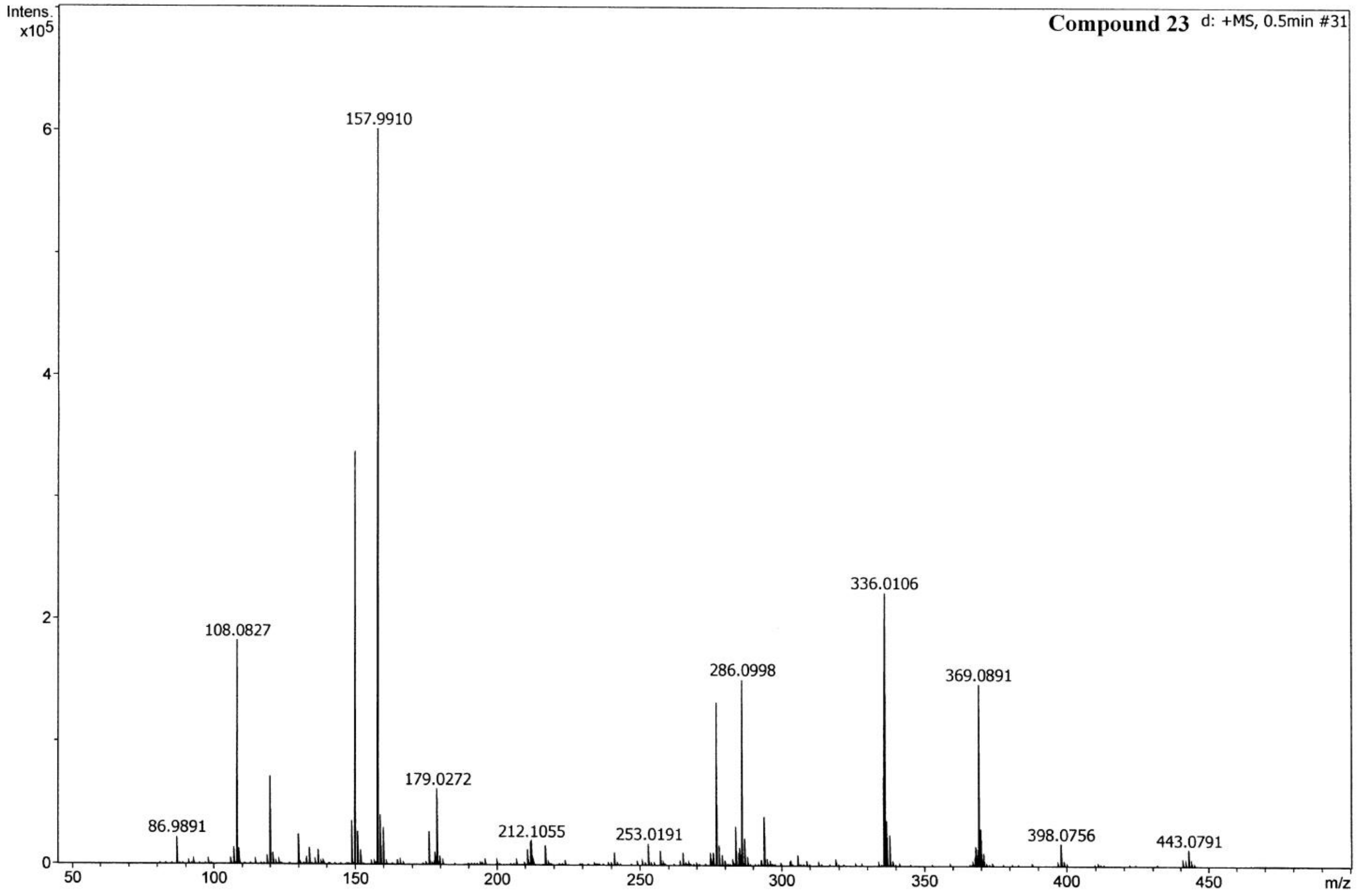

Window Display Report

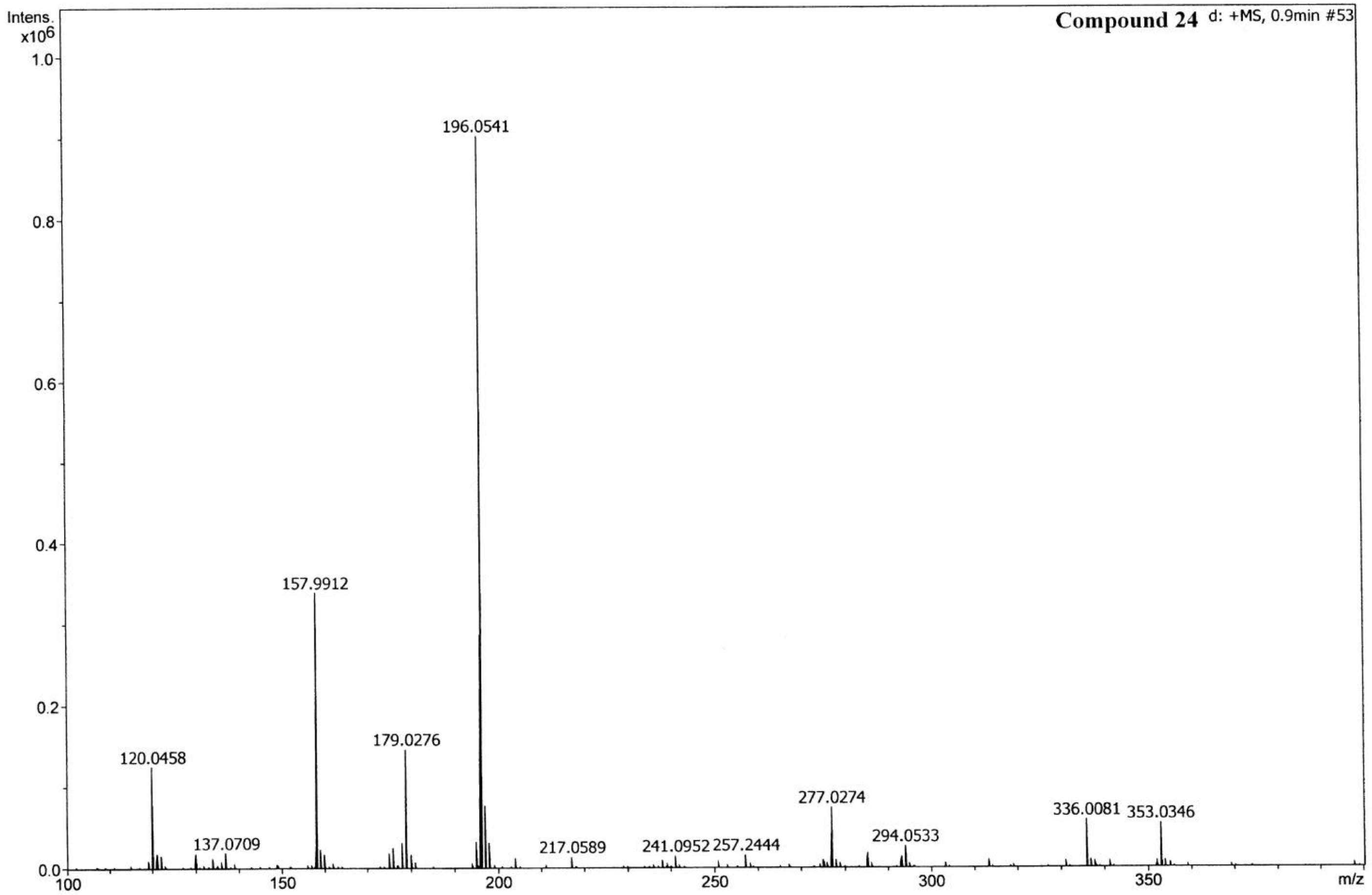

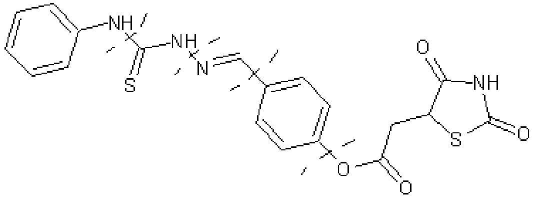

NH
NH
N
S
O
NH
S
O
O
O

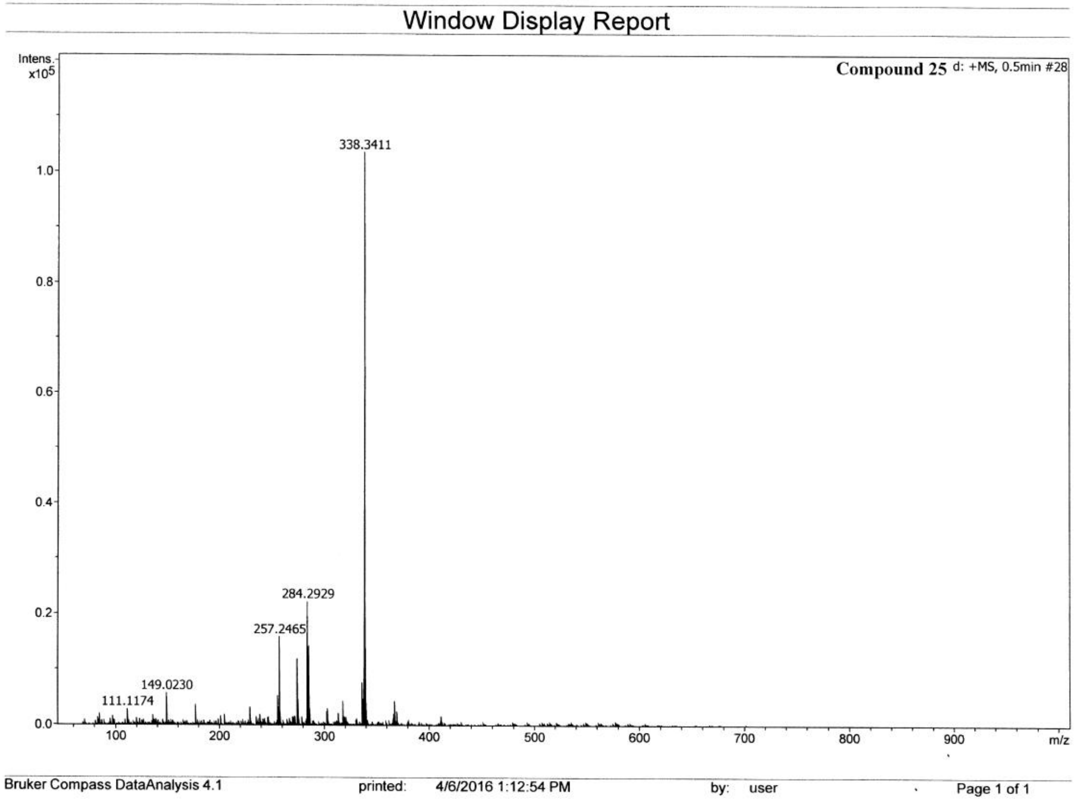

Window Display Report
Intens.
x10^5
Compound 25 d: +MS, 0.5min #28
338.3411
284.2929
257.2465
149.0230
111.1174
m/z
Bruker Compass DataAnalysis 4.1
printed: 4/6/2016 1:12:54 PM
by: user
Page 1 of 1

Window Display Report

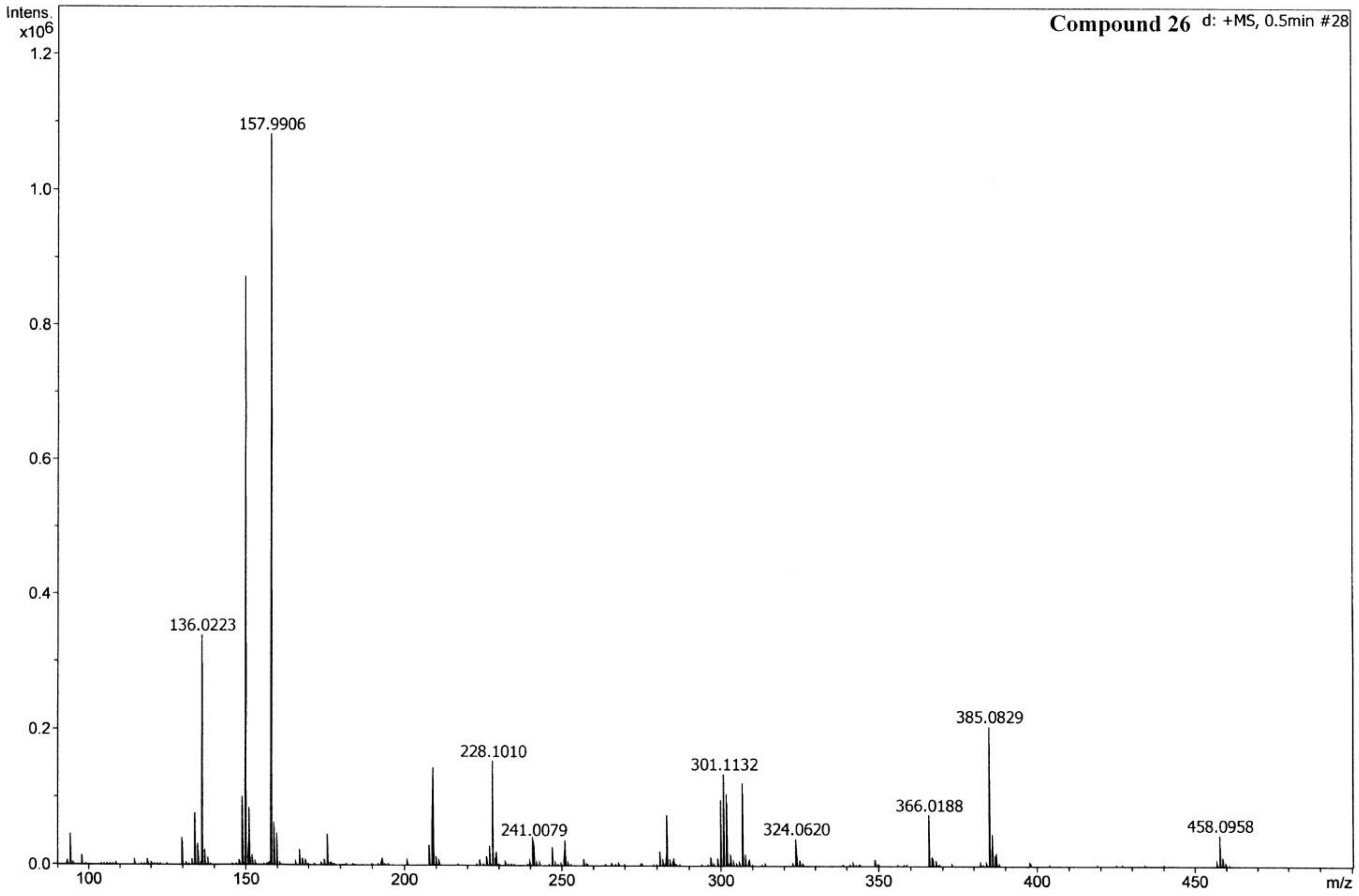

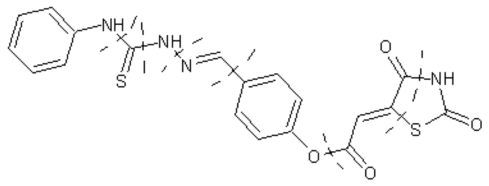
NH
NH
N
S
O
NH
O
S
O
O

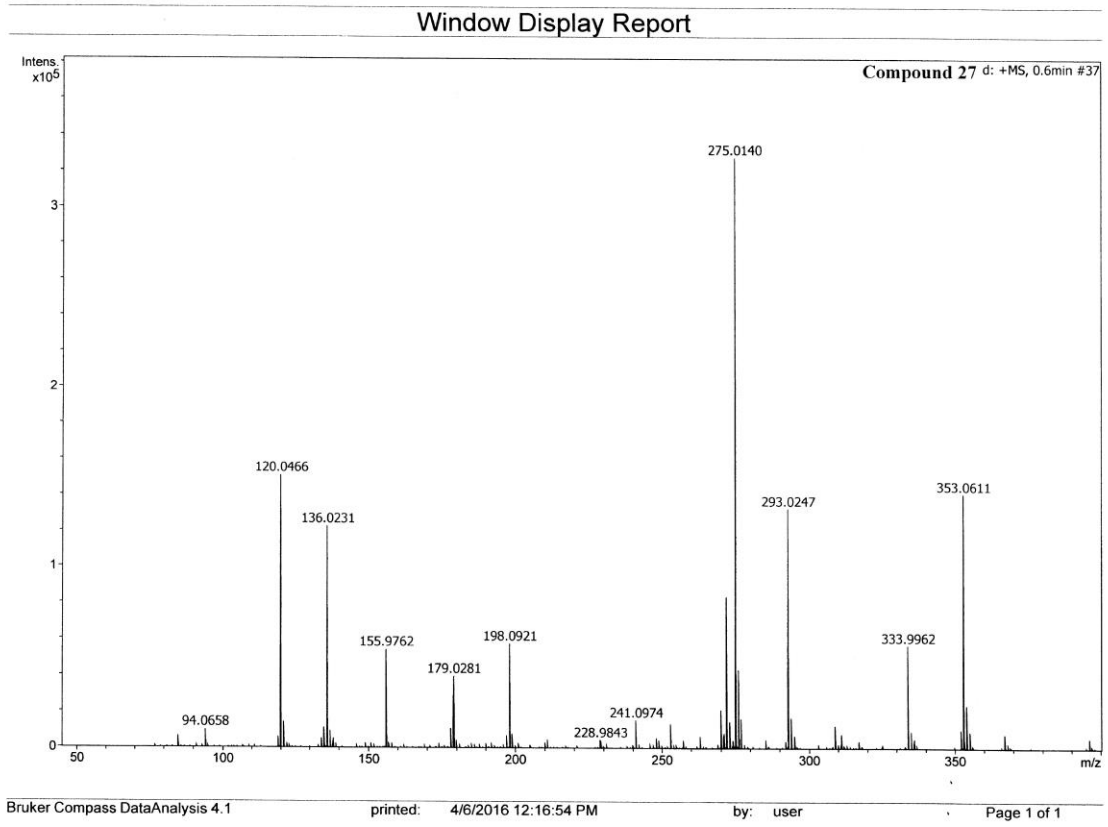
Window Display Report
Intens.
x10^5
Compound 27 d: +MS, 0.6min #37
275.0140
120.0466
136.0231
155.9762
179.0281
198.0921
94.0658
228.9843
241.0974
293.0247
333.9962
353.0611
m/z
Bruker Compass DataAnalysis 4.1
printed: 4/6/2016 12:16:54 PM
by: user
Page 1 of 1

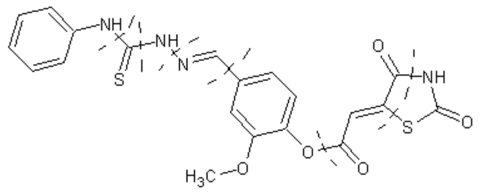

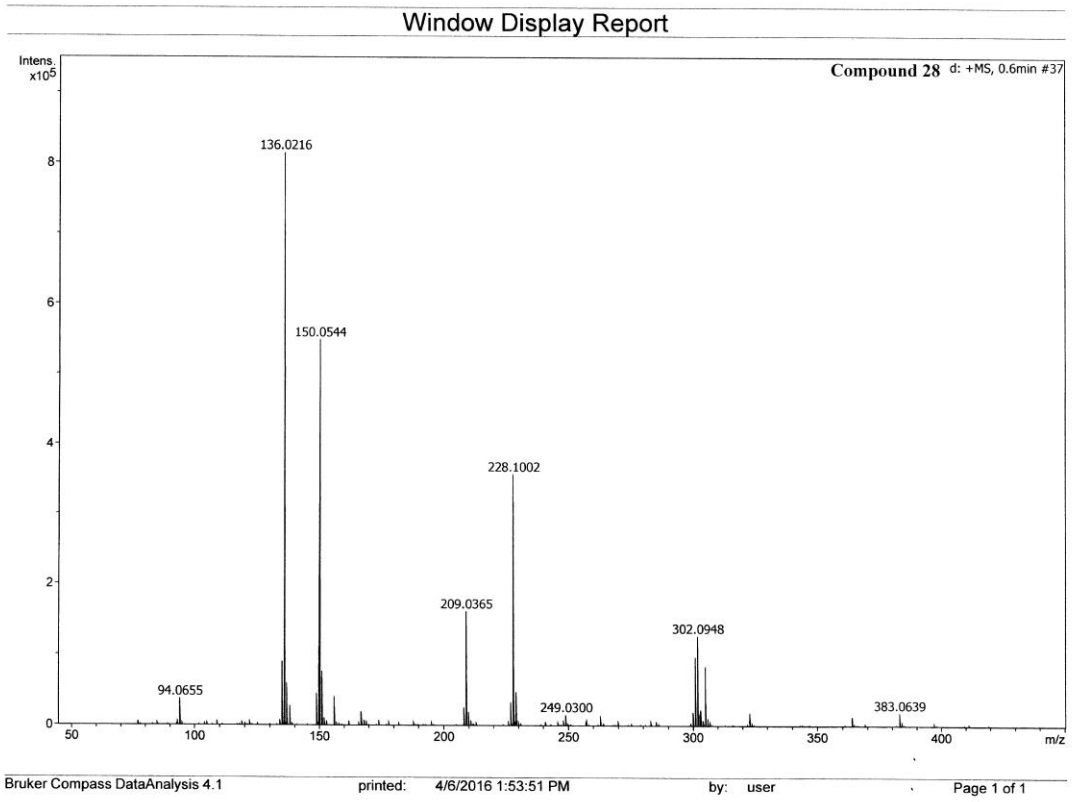
Window Display Report
Intens.
x10^5
Compound 28 d: +MS, 0.6min #37
136.0216
150.0544
228.1002
209.0365
302.0948
94.0655
249.0300
383.0639
m/z
Bruker Compass DataAnalysis 4.1
printed: 4/6/2016 1:53:51 PM
by: user
Page 1 of 1
